# Electrophilic trifluoromethylselenolation of terminal alkynes with *Se*-(trifluoromethyl) 4-methylbenzenesulfonoselenoate

**DOI:** 10.3762/bjoc.13.260

**Published:** 2017-12-07

**Authors:** Clément Ghiazza, Anis Tlili, Thierry Billard

**Affiliations:** 1 Institute of Chemistry and Biochemistry, Univ Lyon, Université Lyon 1, CNRS, 43 Bd du 11 novembre 1918, F-69622 Villeurbanne, France; 2 CERMEP-In vivo Imaging, Groupement Hospitalier Est, 59 Bd Pinel, F-69003 Lyon, France

**Keywords:** alkynes, nucleophilic addition, perfluoroalkylselenolation, *Se*-(trifluoromethyl) 4-methylbenzenesulfonoselenoate, trifluoromethylselenolation

## Abstract

Herein the nucleophilic addition of *Se*-(trifluoromethyl) 4-methylbenzenesulfonoselenoate, a stable and easy-to-handle reagent, to alkynes is described. This reaction provides trifluoromethylselenylated vinyl sulfones with good results and the method was extended also to higher fluorinated homologs. The obtained compounds are valuable building blocks for further syntheses of fluoroalkylselenolated molecules.

## Introduction

Over the last decades, fluorinated compounds have been the subject of growing interest [1–2]. The specific properties introduced by fluorinated groups have contributed to the “success story” of fluorinated molecules. Nowadays, fluorinated compounds find applications in various fields, from life sciences to materials [3–15]. In the objective to design new molecules with specific properties, novel fluorinated substituents have been developed, such as diverse trifluoromethylchalcogeno groups, due to their particular electronic properties [16] and, more especially, to their high lipophilicity [17]. Whereas the CF_3_O and CF_3_S substituents have been largely studied [18–23], the CF_3_Se group, albeit known, has gained only little attention until recently. However, selenylated derivatives present pertinent properties and have found some interest in materials [24], life sciences [25–33] and drug design [34–37]. Furthermore, very recently, the Hansch lipophilicity parameter of CF_3_Se has been determined (π_R_ = 1.29) – a high value lying between that of CF_3_O and CF_3_S [38]. Consequently, trifluoromethylselenolated molecules could represent interesting alternatives in the modulation of properties for various applications.

Despite such potential interest for CF_3_Se compounds, methods to their syntheses remain still limited [39]. Direct trifluoromethylselenolation reactions have recently gained renewed interest and mainly follow two strategies. The nucleophilic approach is based on the use of the CF_3_Se^−^ anion which must be prepared from stoichiometric amounts of metallic selenium [40–53]. Concerning the electrophilic approach, two reagents, that are easy to obtain, have been described: CF_3_SeCl [38,54–67] and CF_3_SeTs [68].

## Results and Discussion

Recently, we have described the electrophilic addition of CF_3_SeCl to alkenes to access α-chloro-β-trifluoromethylselenolated molecules [65]. These products are particularly interesting because the presence of the chlorine substituent opens the way to post-functionalization and thereby to syntheses of more elaborated compounds. However, the similar reaction with alkynes has failed and only a complex mixture was observed which is basically due to the high reactivity of CF_3_SeCl.

To overcome this issue, we have developed another easier-to-use reagent with a more controlled reactivity to perform electrophilic trifluoromethylselenolations, namely *Se*-(trifluoromethyl) 4-methylbenzenesulfonoselenoate (**1a**). This reagent is easily obtained by reacting sodium toluenesulfinate with in situ formed CF_3_SeCl [68]. With this reagent at hand we envisaged the trifluoromethylselenolation of alkynes.

**Scheme 1:**
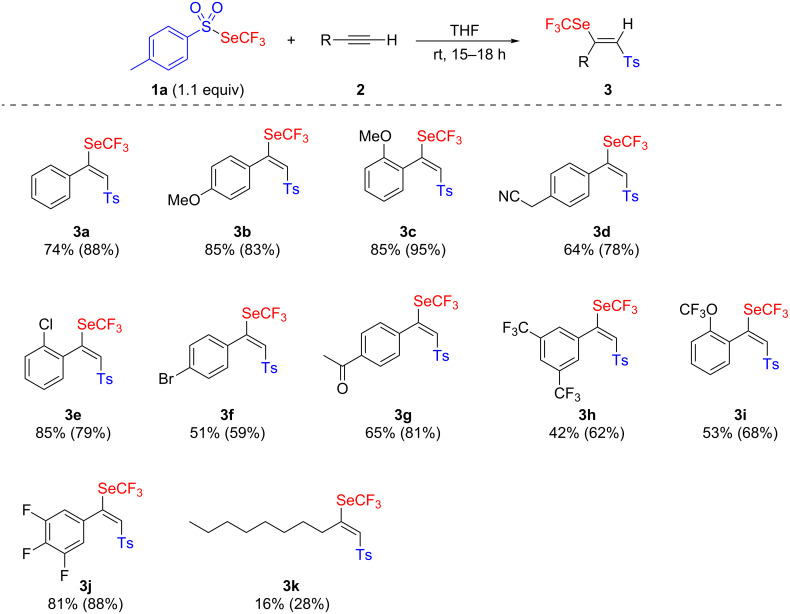
Electrophilic addition of **1a** to alkynes. Yields shown are those of isolated products; yields determined by ^19^F NMR spectroscopy with PhOCF_3_ as an internal standard are shown in parentheses.

To our delight, the addition of **1a** to phenylacetylene (**2a**) at room temperature, without any other practical precautions, lead to the expected addition product **3a** with good yield (Scheme 1).

Subsequently, the reaction was extended to various arylalkynes and afforded, in general, good yields. Satisfactory to excellent results were observed whatever the electronic character (donor or acceptor) of the substituents on the phenyl moiety were. Even highly electron-withdrawing groups led to satisfactory yields of the products **3h**–**j**. The reaction is also not too sensitive to steric hindrance because good results were obtained also with *ortho*-substituted substrates **3c**,**e**,**i**. Nevertheless, the reaction with aliphatic alkyne **2k**, resulted in a low yield (28%).

**Figure 1:**
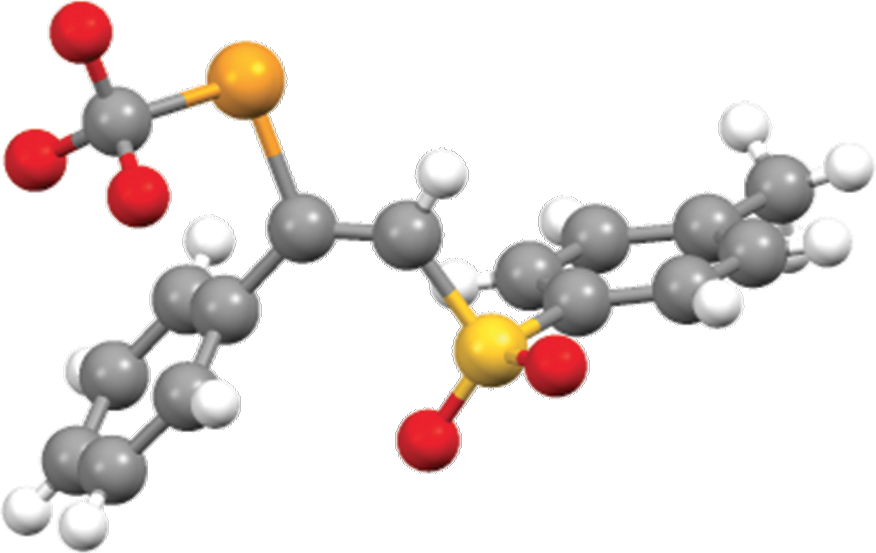
Single-crystal X-ray structure of **3a**.

The reaction is stereoselective with the exclusive formation of the *trans-*isomers. Further, a high regioselectivity is observed but, surprisingly, the *anti*-Markovnikov regioisomers were obtained. The stereochemistry and regiochemistry were confirmed thanks to the X-ray structure of compound **3a** (Figure 1).

**Scheme 2:**
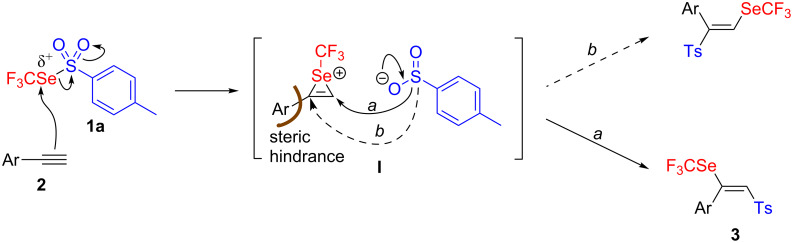
Mechanism proposal.

From a mechanistic point of view, the reaction starts certainly with the intermediate formation of the trifluoromethylselenonium ion **I**. This is in accordance with the observed *trans-*selectivity due to the *anti* opening of **I** by the toluenesulfinate anion. The regioselectivity could be rationalized by the steric hindrance between the aromatic moiety and the large nucleophile (toluenesulfinate anion) which favors the attack on the less hindered side (Scheme 2, pathway a). Such sensitivity to steric hindrance of **I** was confirmed by the lack of reactions observed with internal alkynes.

**Scheme 3:**
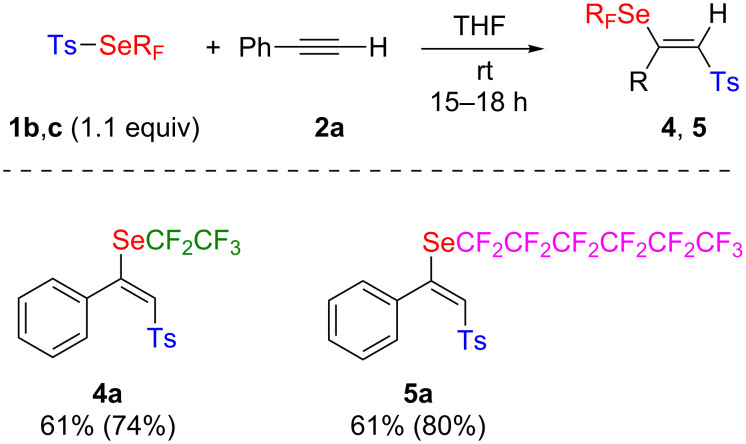
Perfluoroalkylselenolation of alkynes. Yields shown are those of isolated products; yields determined by ^19^F NMR spectroscopy with PhOCF_3_ as an internal standard are shown in parentheses.

Higher homologs of reagent **1a** are also available, the reaction was briefly performed with **1b** and **1c** (Scheme 3). The expected products were obtained with good yields. The tridecafluorohexylselenyl group in product **5a** makes this compound interesting because it opens the way to applications in the design of fluorinated polymers or surfactants.

## Conclusion

To conclude, *Se*-(trifluoromethyl) 4-methylbenzenesulfonoselenoate and *Se*-(perfluoroalkyl) 4-methylbenzenesulfonoselenoate have been confirmed as valuable bench-stable reagents to perform perfluoroalkylselenolations. Nucleophilic additions with alkynes to provide perfluoroalkylselenolated vinyl sulfones can easily be carried out. *Se*-(Trifluoromethyl) 4-methylbenzenesulfonoselenoate and *Se*-(perfluoroalkyl) 4-methylbenzenesulfonoselenoate constitute interesting building blocks for various applications.

## Experimental

**Typical procedure:** To a flask equipped with a magnetic stir bar were added **1** (0.25 mmol, 1.1 equiv), alkyne **2** (0.23 mmol, 1.0 equiv), and anhydrous THF (1 mL). The reaction was stirred at 25 °C for 15–18 hours (conversion was checked by ^19^F NMR with PhOCF_3_ as internal standard). The crude residue was purified by chromatography to afford the desired products **3**–**5**.

## Supporting Information

File 1 Additional experimental and analytical data and NMR spectra.
