# Supplementary material for: Electrophilic trifluoromethylselenolation of terminal alkynes with Se-(trifluoromethyl) 4-methylbenzenesulfonoselenoate
Source: Beilstein J Org Chem. 2017 Dec 7;13:2626–30. doi: 10.3762/bjoc.13.260 (PMC5727788; doi:10.3762/bjoc.13.260)

Supporting Information

for

Electrophilic trifluoromethylselenolation of terminal alkynes with *Se*-(trifluoromethyl) 4-methylbenzene-sulfonoselenoate

Clément Ghiazza[1], Anis Tlili*[1] and Thierry Billard*[1,2]

Address: [1]Institute of Chemistry and Biochemistry, Univ Lyon, Université Lyon 1, CNRS, 43 Bd du 11 novembre 1918, F-69622 Villeurbanne, France and [2]CERMEP-In vivo Imaging, Groupement Hospitalier Est, 59 Bd Pinel, F-69003 Lyon, France

Email: Anis Tlili - anis.tlili@univ-lyon1.fr; Thierry Billard - Thierry.billard@univ-lyon1.fr

* Corresponding author

Additional experimental and analytical data and NMR spectra

General information S3

Typical procedure for the addition to alkynes S3

Synthesis of 1-methyl-4-[(*E*)-2-phenyl-2-[(trifluoromethyl)selanyl]ethenesulfonyl]benzene (3a) S3

Synthesis of 1-[(*E*)-2-(4-methoxyphenyl)-2-[(trifluoromethyl)selanyl]ethenesulfonyl]-4-methylbenzene (3b) S4

Synthesis of 1-[(*E*)-2-(2-methoxyphenyl)-2-[(trifluoromethyl)selanyl]ethenesulfonyl]-4-methylbenzene (3c) S4

Synthesis of 2-{4-[(*E*)-2-(4-methylphenylsulfonyl)-1-[(trifluoromethyl)selanyl]ethenyl]phenyl}acetonitrile (3d) S5

Synthesis of 1-[(*E*)-2-(2-chlorophenyl)-2-[(trifluoromethyl)selanyl]ethenesulfonyl]-4-methylbenzene (3e) S5

Synthesis of 1-[(*E*)-2-(4-bromophenyl)-2-[(trifluoromethyl)selanyl]ethenesulfonyl]-4-methylbenzene (3f) S6

Synthesis of 1-{4-[(*E*)-2-(4-methylphenylsulfonyl)-1-[(trifluoromethyl)selanyl]ethenyl]phenyl}ethan-1-one (3g) S6

Synthesis of 1-[(*E*)-2-(4-methylphenylsulfonyl)-1-[(trifluoromethyl)selanyl]ethenyl]-3,5-bis(trifluoromethyl)benzene (3h) S7

Synthesis of 1-methyl-4-[(*E*)-2-[2-(trifluoromethoxy)phenyl]-2-[(trifluoromethyl)selanyl]ethenesulfonyl]benzene (3i) S7

Synthesis of 1,2,3-trifluoro-5-[(*E*)-2-(4-methylphenylsulfonyl)-1-[(trifluoromethyl)selanyl]ethenyl]benzene (3j) S8

Synthesis of 1-methyl-4-[(1*E*)-2-[(trifluoromethyl)selanyl]dec-1-ene-1-sulfonyl]benzene (3k) S8

Synthesis of 1-methyl-4-[(*E*)-2-[(1,1,2,2,2-pentafluoroethyl)selanyl]-2-phenylethenesulfonyl]benzene (4a) S9

Synthesis of 1-methyl-4-[(*E*)-2-phenyl-2-[(1,1,2,2,3,3,4,4,5,5,6,6,6-tridecafluorohexyl)selanyl]ethenesulfonyl]benzene (5a) S9

References S9

X-ray structure of product 3a S10

NMR and GC–MS spectra S14

General Information

Commercial reagents were used as supplied. 1-Methyl-4-{[(perfluoroalkyl)selanyl]sulfonyl}benzene derivatives **1a**, **1b** or **1c** were synthesized following procedures described in the literature.[1] Anhydrous solvents were used as supplied. NMR spectra were recorded on a Bruker AV 400 spectrometer at 400 MHz (^{1}H NMR), 101 MHz (^{13}C NMR), 376 MHz (^{19}F NMR) or on a Bruker AV 300 spectrometer at 300 MHz (^{1}H NMR), 282 MHz (^{19}F NMR). Multiplicities are indicated as follows: s (singlet), d (doublet), t (triplet), q (quartet), p (quintet), sext (sextet), m (multiplet), b (broad). All coupling constants were reported in Hz. Melting points were determined using a Kofler bench apparatus (calibration substances were specified). Gas chromatography–mass analysis was carried out on an Agilent HP-5890 instrument with an Agilent HP-5973 Mass Selective Detector (EI, 70 eV) and HP-5 capillary column (polydimethylsiloxane with 5% phenyl groups, 30 m, 0.25 mm i.d., 0.25 µm film thickness) using helium carrier gas.

Typical procedure for the addition to alkynes

To a flask equipped with a magnetic stir bar are added **1a**, **1b** or **1c** (0.25 mmol, 1.1 equiv), alkyne **2** (0.23 mmol, 1.0 equiv), and anhydrous THF (1 mL). The reaction is stirred at 25 °C for 15–18 hours (conversion is checked by ^{19}F NMR with $PhOCF_3$ as internal standard). The crude residue is purified by chromatography to afford the desired product **3**, **4** or **5**.

Synthesis of 1-methyl-4-[(*E*)-2-phenyl-2-[(trifluoromethyl)selanyl]ethenesulfonyl]benzene (3a)

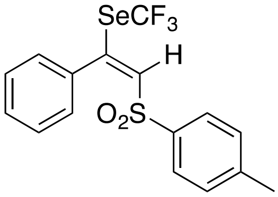

Off white to yellow solid (mp: 58 – 60°C, calibration substance: Azobenzol at 68.0°C)
Eluent for the flash chromatography: Cyclohexane/EtOAc: 95/5 to 90/10
^{1}H NMR (400 MHz, $CDCl_3$) δ = 7.43 (m, 2H), 7.39 (m, 1H), 7.36-7.27 (m, 1H), 7.17 (m, 2H), 7.08 (s, 1H), 2.39 (s, 3H).
^{13}C NMR (101 MHz, $CDCl_3$) δ = 144.7 (q, ^{4}J(C,F) = 1 Hz), 144.7, 137.5, 134.8 (q, ^{3}J(C,F) = 2 Hz), 133.8, 130.5, 129.7, 129.4, 128.2, 127.9, 122.3 (q, ^{1}J(C,F) = 335 Hz), 21.8.
^{19}F NMR (376 MHz, $CDCl_3$) δ = -33.72 (s, 3F).
MS (EI): *m/z* (%), 405.9 (21), 336.9 (26), 257.0 (14), 193.0 (78), 179.9 (17), 154.9 (50), 138.9 (29), 122.9 (10), 105.0 (22), 91.0 (100), 77.0 (6), 65.0 (22).

Synthesis of 1-[(*E*)-2-(4-methoxyphenyl)-2-[(trifluoromethyl)selanyl]ethenesulfonyl]-4-methylbenzene (3b)

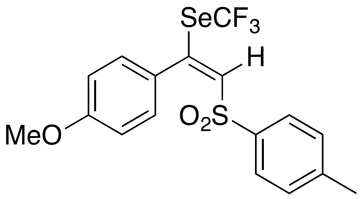

Yellow solid (mp: 108 – 110°C, calibration substance: Acetanilid at 114.5°C)
Eluent for the flash chromatography: Cyclohexane/EtOAc: 90/10 to 80/20
^{1}H NMR (400 MHz, $CDCl_3$) δ = 7.48 (m, 2H), 7.30 (m, 2H), 7.19 (dd, *J* = 8.5, 0.6 Hz, 2H), 7.01 (s, 1H), 6.84 (m, 2H), 3.84 (s, 1H), 2.39 (s, 1H).
^{13}C NMR (101 MHz, $CDCl_3$) δ = 161.6, 144.8, 144.6, 137.7, 133.9 (q, ^{3}J(C,F) = 2 Hz), 131.5, 129.7, 127.9, 125.9 (q, ^{4}J(C,F) = 1 Hz), 122.3 (q, ^{1}J(C,F) = 335 Hz), 113.7, 55.5, 21.8.
^{19}F NMR (376 MHz, $CDCl_3$) δ = -33.86 (s, 3F).
MS (EI): *m/z* (%), 435.9 (12), 287.0 (36), 223.0 (100), 154.9 (29), 131.9 (60), 116.9 (25), 90.9 (83), 64.9 (16).

Synthesis of 1-[(*E*)-2-(2-methoxyphenyl)-2-[(trifluoromethyl)selanyl]ethenesulfonyl]-4-methylbenzene (3c)

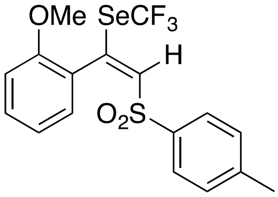

Yellow solid (mp: 66 – 68°C, calibration substance: Azobenzol at 68.0°C)
Eluent for the flash chromatography: Cyclohexane/EtOAc: 80/20
^{1}H NMR (400 MHz, $CDCl_3$) δ = 7.41 (m, 2H), 7.33 (ddd, *J* = 8.4, 7.5, 1.7 Hz, 1H), 7.22 (dd, *J* = 7.5, 1.7 Hz, 1H), 7.15-7.13 (m, 3H), 6.95 (td, *J* = 7.5, 0.9 Hz, 1H), 6.68 (m, 1H), 3.59 (s, 3H), 2.38 (s, 3H).
^{13}C NMR (101 MHz, $CDCl_3$) δ = 155.6, 144.4, 140.3 (q, ^{4}J(C,F) = 1 Hz), 137.7 (q, ^{3}J(C,F) = 2 Hz), 137.1, 131.8, 130.5, 129.3, 128.0, 123.0, 122.5 (q, ^{1}J(C,F) = 334 Hz), 120.2, 110.5, 55.3, 21.7.
^{19}F NMR (376 MHz, $CDCl_3$) δ = -33.82 (s, 3F).
MS (EI): *m/z* (%), 435.9 (26), 287.0 (26), 208.0 (48), 195.0 (10), 154.9 (33), 131.0 (64), 105.0 (49), 91.0 (100), 65.0 (18).

Synthesis of 2-{4-[(*E*)-2-(4-methylphenylsulfonyl)-1-[(trifluoromethyl)selanyl]ethenyl]phenyl}acetonitrile (3d)

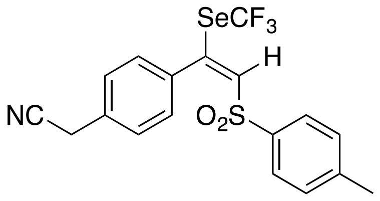

White solid (mp: 118 – 120°C, calibration substance: Acetanilid at 114.5°C)
Eluent for the flash chromatography: Cyclohexane/EtOAc: 85/15
^{1}H NMR (400 MHz, $CDCl_3$) δ = 7.47 (m, 2H), 7.34 (m, 2H), 7.31 (m, 2H), 7.23 (dd, *J* = 8.5, 0.5 Hz, 2H), 7.09 (s, 1H), 3.78 (s, 2H), 2.41 (s, 3H).
^{13}C NMR (101 MHz, $CDCl_3$) δ = 145.1, 143.1 (q, ^{4}J(C,F) = 1 Hz), 137.2, 135.6 (q, ^{3}J(C,F) = 2 Hz), 134.0, 132.5, 130.1, 129.9, 127.9, 127.8, 122.1 (q, ^{1}J(C,F) = 335 Hz), 117.4, 23.6, 21.7.
^{19}F NMR (376 MHz, $CDCl_3$) δ = -33.42 (s, 3F).
MS (EI): *m/z* (%), 444.8 (5), 375.9 (10), 232.0 (42), 206.9 (37), 154.9 (71), 139.9 (40), 90.9 (100), 64.9 (23).

Synthesis of 1-[(*E*)-2-(2-chlorophenyl)-2-[(trifluoromethyl)selanyl]ethenesulfonyl]-4-methylbenzene (3e)

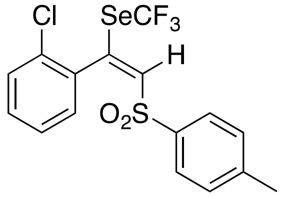

Yellow oil
Eluent for the flash chromatography: Cyclohexane/EtOAc: 90/10
^{1}H NMR (400 MHz, $CDCl_3$) δ = 7.49 (d, *J* = 8.2 Hz, 2H), 7.38-7.29 (m, 4H), 7.22 (d, *J* = 8.2 Hz, 2H), 7.15 (s, 1H), 2.41 (s, 3H).
^{13}C NMR (101 MHz, $CDCl_3$) δ = 145.2, 139.7, 138.3, 136.6, 132.9, 132.2, 131.3, 130.9, 129.9, 129.7, 128.2, 126.7, 122.3 (q, ^{1}J(C,F) = 334 Hz), 21.8.
^{19}F NMR (376 MHz, $CDCl_3$) δ = -33.33 (s, 3F).
MS (EI): *m/z* (%), 404.9 (94), 227.0 (42), 154.9 (62), 135.9 (40), 91.0 (100), 65.0 (22).

Synthesis of 1-[(*E*)-2-(4-bromophenyl)-2-[(trifluoromethyl)selanyl]ethenesulfonyl]-4-methylbenzene (3f)

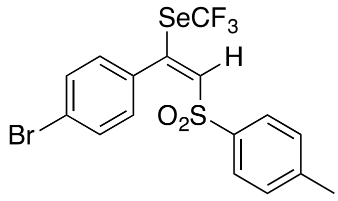

White solid (mp: 110 – 112°C, calibration substance: Acetanilid at 114.5°C)
Eluent for the flash chromatography: Cyclohexane/EtOAc: 90/10
^{1}H NMR (400 MHz, $CDCl_3$) δ = 7.49-7.45 (m, 4H), 7.23 (d, *J* = 8.0 Hz, 1H), 7.18 (m, 2H), 7.09 (s, 1H), 2.42 (s, 3H).
^{13}C NMR (101 MHz, $CDCl_3$) δ = 145.1, 142.8, 137.3, 135.8, 132.9, 131.5, 131.0, 129.9, 128.0, 125.2, 122.1 (q, ^{1}J(C,F) = 335 Hz), 21.8.
^{19}F NMR (376 MHz, $CDCl_3$) δ = -33.40 (s, 3F).
MS (EI): *m/z* (%), 483.9 (11), 414.8 (7), 336.9 (9), 270.9 (43), 179.9 (24), 154.9 (69), 138.9 (24), 91.0 (100), 65.0 (21).

Synthesis of 1-{4-[(*E*)-2-(4-methylphenylsulfonyl)-1-[(trifluoromethyl)selanyl]ethenyl]phenyl}ethan-1-one (3g)

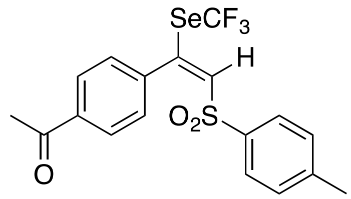

White solid (mp: 122 – 124°C, calibration substance: Acetanilid at 114.5°C)
Eluent for the flash chromatography: Cyclohexane/EtOAc: 85/15 to 80/20
^{1}H NMR (400 MHz, $CDCl_3$) δ = 7.93 (m, 2H), 7.50 (m, 2H), 7.42 (m, 2H), 7.23 (dd, *J* = 8.5, 0.6 Hz, 2H), 7.11 (s, 1H), 2.63 (s, 3H), 2.41 (s, 3H).
^{13}C NMR (101 MHz, $CDCl_3$) δ = 197.2, 145.1, 142.5 (q, ^{4}J(C,F) = 1 Hz), 138.5, 138.1, 137.1, 135.9 (q, ^{3}J(C,F) = 2 Hz), 129.9, 129.5, 128.0, 127.8, 122.0 (q, ^{1}J(C,F) = 335 Hz), 26.7, 21.7.
^{19}F NMR (376 MHz, $CDCl_3$) δ = -33.22 (s, 3F).
MS (EI): *m/z* (%), 447.9 (11), 378.9 (11), 235.0 (37), 208.8 (15), 154.9 (71), 128.9 (40), 90.9 (100), 65.0 (20).

Synthesis of 1-[(*E*)-2-(4-methylphenylsulfonyl)-1-[(trifluoromethyl)selanyl]ethenyl]-3,5-bis(trifluoromethyl)benzene (3h)

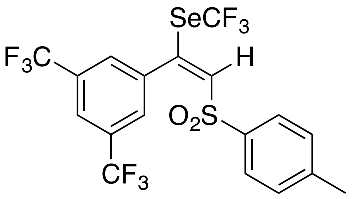

White solid (mp: 94 – 96°C, calibration substance: Benzil at 95.0°C)
Eluent for the flash chromatography: Cyclohexane/EtOAc: 90/10
^{1}H NMR (400 MHz, $CDCl_3$) δ = 7.88 (s, 1H), 7.68 (s, 2H), 7.42 (m, 2H), 7.28 (s, 1H), 7.22 (d, J = 8.3 Hz, 2H), 2.40 (s, 3H).
^{13}C NMR (101 MHz, $CDCl_3$) δ = 145.8, 139.6, 138.8 (q, ^{4}J(C,F) = 2 Hz), 136.6, 136.4, 131.8 (q, ^{2}J(C,F) = 34 Hz), 130.1, 129.4 (q, ^{3}J(C,F) = 3 Hz), 127.9, 123.9 (m), 122.8 (q, ^{1}J(C,F) = 273 Hz), 121.9 (q, ^{1}J(C,F) = 335 Hz), 21.7.
^{19}F NMR (376 MHz, $CDCl_3$) δ = -32.84 (s, 3F), -63.09 (s, 6F).
MS (EI): *m/z* (%), 541.9 (10), 522.9 (8), 472.9 (55), 408.9 (10), 317.9 (47), 237.9 (22), 218.9 (24), 154.9 (65), 138.9 (72), 91.0 (100), 65.0 (27).

Synthesis of 1-methyl-4-[(*E*)-2-[2-(trifluoromethoxy)phenyl]-2-[(trifluoromethyl)selanyl]ethenesulfonyl]benzene (3i)

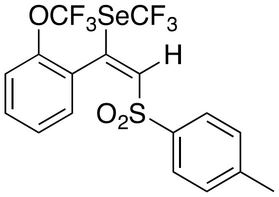

Yellow oil
Eluent for the flash chromatography: Cyclohexane/EtOAc: 80/20
^{1}H NMR (400 MHz, $CDCl_3$) δ = 7.53 (m, 2H), 7.46 (ddd, J = 8.4, 7.6, 1.9 Hz, 1H), 7.37 (dd, J = 7.6, 1.9 Hz, 1H), 7.30 (td, J = 7.6, 1.0 Hz, 1H), 7.25 (m, 2H), 7.20 (m, 1H), 7.12 (s, 1H), 2.42 (s, 2H).
^{13}C NMR (101 MHz, $CDCl_3$) δ = 145.8 (q, ^{4}J(C,F) = 1 Hz), 145.3, 138.0 (q, ^{3}J(C,F) = 2 Hz), 137.1 (q, ^{4}J(C,F) = 1 Hz), 136.8, 131.9, 131.3, 129.9, 128.1, 126.1, 125.8, 122.3 (q, ^{1}J(C,F) = 334 Hz), 120.3 (q, ^{1}J(C,F) = 260 Hz), 118.3 (q, ^{3}J(C,F) = 2 Hz), 21.8.
^{19}F NMR (376 MHz, $CDCl_3$) δ = -33.45 (s, 3F), -56.75 (s, 3F).
MS (EI): *m/z* (%), 490.0 (2), 420.9 (26), 341.0 (18), 265.9 (13), 185.9 (25), 154.9 (67), 138.9 (27), 91.0 (100), 65.0 (20).

Synthesis of 1,2,3-trifluoro-5-[(*E*)-2-(4-methylphenylsulfonyl)-1-[(trifluoromethyl)selanyl]ethenyl]benzene (3j)

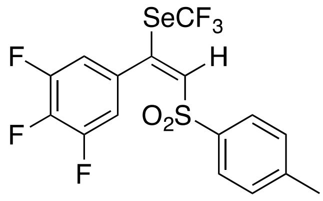

White solid (mp: 110 – 112°C, calibration substance: Acetanilid at 114.5°C)
Eluent for the flash chromatography: Cyclohexane/EtOAc: 90/10
^{1}H NMR (400 MHz, $CDCl_3$) δ = 7.55 (m, 2H), 7.30 (d, J = 8.3 Hz, 2H), 7.12 (s, 1H), 6.97 (dd, ^{5}J(H,F) = 7.5 Hz, ^{3}J(H,F) = 6.4 Hz, 2H), 2.44 (s, 1H).
^{13}C NMR (101 MHz, $CDCl_3$) δ = 150.8 (ddd, ^{1}J(C,F) = 253 Hz, ^{3}J(C,F) = 10 Hz, ^{5}J(C,F) = 4 Hz), 145.7, 140.96 (dt, ^{1}J(C,F) = 257 Hz, ^{3}J(C,F) = 15 Hz), 139.6, 137.6 (d, ^{4}J(C,F) = 1.0 Hz), 136.9, 130.3, 130.1, 128.0, 122.0 (q, ^{1}J(C,F) = 335 Hz), 114.1 (dd, ^{2}J(C,F) = 17 Hz, ^{4}J(C,F) = 6.7 Hz), 21.8.
^{19}F NMR (376 MHz, $CDCl_3$) δ = -33.12 (s, 3F), -132.72 (dd, ^{3}J(F,F) = 20.5 Hz, ^{3}J(F,H) = 7.5 Hz, 2F), -155.99 (tt, ^{3}J(F,F) = 20.5 Hz, ^{5}J(F,H) = 6.4 Hz, 1F).
MS (EI): *m/z* (%), 459.9 (16), 390.9 (44), 235.8 (42), 154.9 (82), 138.9 (50), 91.0 (100), 65.0 (26).

Synthesis of 1-methyl-4-[(1*E*)-2-[(trifluoromethyl)selanyl]dec-1-ene-1-sulfonyl]benzene (3k)

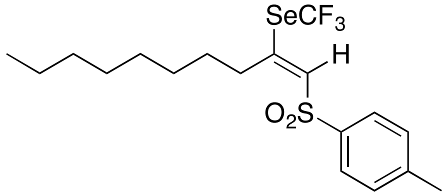

White solid (mp <50, calibration substance: Azobenzol at 68.0°C)
Eluent for the flash chromatography: Cyclohexane/Toluene: 70/30
^{1}H NMR (400 MHz, $CDCl_3$) δ = 7.79 (m, 2H), 7.36 (dd, J = 8.6, 0.6 Hz, 2H), 6.78 (s, 1H), 3.94 (m, 2H), 2.45 (s, 3H), 1.54 (m, 2H), 1.34-1.22 (m, 10H), 0.89 (t, J = 6.9 Hz, 3H).
^{13}C NMR (101 MHz, $CDCl_3$) δ = 148.1 (q, ^{4}J(C,F) = 1 Hz), 145.1, 138.2, 135.6 (q, ^{3}J(C,F) = 2 Hz), 130.2, 127.6, 122.3 (q, ^{1}J(C,F) = 333 Hz), 34.5, 31.9, 29.4, 29.3, 29.2, 29.0, 22.8, 21.8, 14.2.
^{19}F NMR (376 MHz, $CDCl_3$) δ = -33.10 (s, 3F).
MS (EI): *m/z* (%), 373.0 (7), 293.1 (25), 156.9 (100), 138.9 (55), 90.9 (82), 81.0 (32), 64.9 (25), 55.0 (21).

Synthesis of 1-methyl-4-[(*E*)-2-[(1,1,2,2,2-pentafluoroethyl)selanyl]-2-phenylethenesulfonyl]benzene (4a)

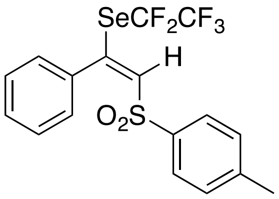

Off white solid (mp: 56 – 58°C, calibration substance: Azobenzol at 68.0°C)
Eluent for the flash chromatography: Cyclohexane/EtOAc: 95/5 to 90/10
^{1}H NMR (400 MHz, $CDCl_3$) δ = 7.44 (m, 2H), 7.39 (m, 1H), 7.35-7.29 (m, 4H), 7.20-7.15 (m, 3H), 2.39 (s, 3H).
^{13}C NMR (101 MHz, $CDCl_3$) δ = 144.8, 142.6 (t, ^{3}J(C,F) = 2 Hz) 137.5, 137.3, 134.6, 130.6, 129.7, 129.7, 128.2, 128.0, 118.5 (qt, ^{1}J(C,F) = 286 Hz, ^{2}J(C,F) = 34 Hz), 116.6 (tq, ^{1}J(C,F) = 308 Hz, ^{2}J(C,F) = 43 Hz), 21.8.
^{19}F NMR (376 MHz, $CDCl_3$) δ = -82.95 (t, ^{3}J(F,F) = 4.0 Hz), -89.65 (q, ^{3}J(F,F) = 4.0 Hz).
MS (EI): *m/z* (%), 455.9 (11), 336.9 (59), 193.0 (73), 154.9 (52), 138.9 (45), 91.0 (100), 65.0 (22).

Synthesis of 1-methyl-4-[(*E*)-2-phenyl-2-[(1,1,2,2,3,3,4,4,5,5,6,6,6-tridecafluorohexyl)selanyl]ethenesulfonyl]benzene (5a)

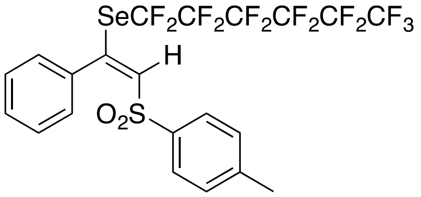

Off white solid (mp: 84 – 86°C, calibration substance: Benzil at 95.0°C)
Eluent for the flash chromatography: Cyclohexane/EtOAc: 95/5
^{1}H NMR (400 MHz, $CDCl_3$) δ = 7.44 (m, 2H), 7.39 (m, 1H), 7.34-7.29 (m, 4H), 7.20-7.16 (m, 3H), 2.39 (s, 3H).
^{13}C NMR (101 MHz, $CDCl_3$) δ = 144.8, 142.8 (b), 137.6 (b), 137.3, 134.7, 130.6, 129.7 (m), 128.2, 128.0, 21.7.
^{19}F NMR (376 MHz, $CDCl_3$) δ = -80.79 (tt, ^{3}J(F,F) = 10.0 Hz, ^{5}J(F,F) = 2.3 Hz, 3F), -84.44 (m, 2F), -117.85 (m, 2F), -121.57 (m, 2F), -122.78 (m, 2F), -126.14 (m, 2F).
MS (EI): *m/z* (%), 336.9 (84), 272.9 (14), 193.0 (88), 177.9 (20), 154.9 (65), 138.9 (70), 122.9 (12), 91.0 (100), 65.0 (21).

References

[1] Q. Glenadel, C. Ghiazza, A. Tlili, T. Billard, *Adv. Synth. Catal.*, doi: 10.1002/adsc.201700904.

X-ray structure of product 3a

All data for compound **3a** has been deposited with the Cambridge Crystallographic Database
with CCDC number 1571412.

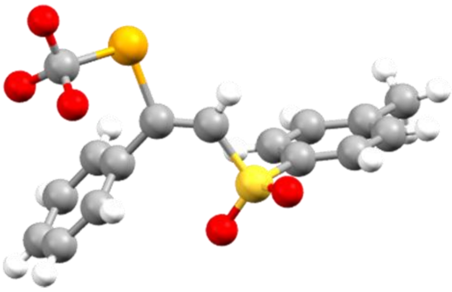

Crystal data

$C_{16}H_{13}O_5SSe$	*F*(000) = 1592
M_r = 396.28	D_x = 1.385 Mg m^{-3}
Orthorhombic, *Aba*2	Mo *K*α radiation, λ = 0.71073 Å
Hall symbol: A 2 -2ac	Cell parameters from 6241 reflections
a = 26.229 (2) Å	θ = 3.9–28.9°
b = 26.135 (3) Å	μ = 2.10 mm^{-1}
c = 5.5458 (6) Å	*T* = 150 K
V = 3801.6 (7) Å^3	Needle, colorless
Z = 8	0.51 × 0.18 × 0.08 mm

Data collection

Xcalibur, Atlas, Gemini ultra diffractometer	4803 independent reflections
Radiation source: fine-focus sealed X-ray tube, Enhance (Mo) X-ray Source	4136 reflections with $I > 2.0\sigma(I)$
Graphite monochromator	R_{int} = 0.073
Detector resolution: 10.4685 pixels mm^{-1}	θ_{max} = 29.5°, θ_{min} = 2.8°
□ scans	*h* = -35→36
Absorption correction: analytical *CrysAlis PRO* 1.171.38.43 (Rigaku Oxford Diffraction, 2015) Analytical numeric absorption correction using a multifaceted crystal model based on expressions derived by R.C. Clark & J.S. Reid. (Clark, R. C. & Reid, J. S. (1995). Acta Cryst. A51, 887-897) Empirical absorption correction using spherical harmonics, implemented in SCALE3 ABSPACK scaling algorithm.	*k* = -34→33
T_{min} = 0.535, T_{max} = 0.845	*l* = -7→6
27560 measured reflections	

Refinement

Refinement on F^2	Hydrogen site location: difference Fourier map
Least-squares matrix: full	H-atom parameters constrained
$R[F^2 > 2□(F^2)] = 0.049$	Method, part 1, Chebychev polynomial, (Watkin, 1994, *P*rince, 1982) [*w*eight] = $1.0/[A_0*T_0(x) + A_1*T_1(x) \cdots + A_{n-1}]*T_{n-1}(x)]$ where A_i are the Chebychev coefficients listed below*w* and x = *F* /*F*max Method = Robust Weighting (*P*rince, 1982) W = [*w*eight] * $[1-(\text{delta}F/6*\text{sigma}F)^2]^2$ A_i are: 632. 963. 481. 156.
$wR(F^2) = 0.107$	$(\Delta/\sigma)_{max} = 0.001$
$S = 0.95$	$\Delta\rangle_{max} = 0.79$ e Å^{-3}
4792 reflections	$\Delta\rangle_{min} = -0.80$ e Å^{-3}
209 parameters	Absolute structure: Flack (1983), 2366 Friedel-pairs
1 restraint	Absolute structure parameter: 0.052 (15)
Primary atom site location: structure-invariant direct methods	

Fractional atomic coordinates and isotropic or equivalent isotropic displacement parameters (Å^2)

	x	*y*	*z*	$U_{iso}*/U_{eq}$
Se1	0.694287 (17)	0.186207 (17)	1.0553 (2)	0.0260
C2	0.65663 (17)	0.22013 (17)	0.7970 (8)	0.0192
C3	0.60253 (17)	0.20462 (18)	0.7643 (8)	0.0208
C4	0.58794 (16)	0.17714 (18)	0.5595 (14)	0.0292
C5	0.53775 (18)	0.16110 (19)	0.5344 (12)	0.0316
C6	0.50156 (19)	0.1742 (2)	0.7069 (9)	0.0302
C7	0.51593 (18)	0.2018 (2)	0.9076 (9)	0.0286
C8	0.56647 (18)	0.2164 (2)	0.9374 (9)	0.0252
H81	0.5762	0.2338	1.0756	0.0306*
H71	0.4907	0.2108	1.0205	0.0337*
H61	0.4677	0.1641	0.6883	0.0359*
H51	0.5281	0.1404	0.4002	0.0378*
H41	0.6123	0.1700	0.4411	0.0353*
C9	0.68396 (17)	0.25565 (18)	0.6905 (8)	0.0222
S10	0.66131 (4)	0.29945 (4)	0.4717 (3)	0.0221
O11	0.69889 (14)	0.30075 (14)	0.2842 (7)	0.0285
O12	0.60898 (14)	0.28916 (14)	0.4106 (7)	0.0292

C13	0.66372 (18)	0.35794 (18)	0.6310 (9)	0.0251
C14	0.6318 (2)	0.3639 (2)	0.8303 (10)	0.0328
C15	0.6328 (2)	0.4095 (2)	0.9544 (12)	0.0380
C16	0.6643 (3)	0.4495 (2)	0.8845 (10)	0.0377
C17	0.6648 (3)	0.4995 (2)	1.0201 (16)	0.0584
H171	0.6948	0.5183	0.9770	0.0880*
H172	0.6648	0.4924	1.1919	0.0881*
H173	0.6343	0.5183	0.9773	0.0880*
C18	0.6963 (2)	0.4424 (2)	0.6886 (10)	0.0365
C19	0.69623 (19)	0.39603 (17)	0.5612 (15)	0.0308
H191	0.7186	0.3911	0.4333	0.0371*
H181	0.7188	0.4681	0.6448	0.0440*
H151	0.6115	0.4138	1.0878	0.0462*
H141	0.6100	0.3380	0.8759	0.0395*
H91	0.7190	0.2584	0.7314	0.0263*
C20	0.6795 (2)	0.1153 (2)	0.9592 (14)	0.0487
O21	0.6830 (2)	0.10830 (15)	0.7288 (9)	0.0501
O22	0.71215 (16)	0.08499 (13)	1.0718 (13)	0.0532
O23	0.63220 (14)	0.10082 (14)	1.0226 (11)	0.0491

Atomic displacement parameters (Å^2)

	U^{11}	U^{22}	U^{33}	U^{12}	U^{13}	U^{23}
Se1	0.02328 (19)	0.0263 (2)	0.0283 (2)	-0.00093 (18)	-0.0022 (3)	0.0019 (3)
C2	0.020 (2)	0.018 (2)	0.019 (2)	0.0023 (16)	-0.0001 (17)	0.0005 (16)
C3	0.018 (2)	0.023 (2)	0.021 (2)	-0.0007 (17)	-0.0011 (17)	-0.0002 (17)
C4	0.026 (2)	0.036 (2)	0.026 (2)	-0.0075 (18)	0.003 (3)	-0.006 (3)
C5	0.031 (2)	0.034 (2)	0.030 (3)	-0.0101 (19)	-0.003 (2)	-0.004 (2)
C6	0.021 (2)	0.034 (3)	0.035 (3)	-0.008 (2)	-0.005 (2)	0.003 (2)
C7	0.019 (2)	0.033 (3)	0.034 (3)	-0.001 (2)	0.0030 (19)	0.000 (2)
C8	0.022 (2)	0.031 (3)	0.022 (2)	-0.0001 (18)	0.0012 (19)	-0.005 (2)
C9	0.020 (2)	0.022 (2)	0.025 (2)	0.0029 (17)	-0.0003 (18)	-0.0019 (18)
S10	0.0185 (5)	0.0229 (5)	0.0248 (5)	-0.0005 (4)	-0.0014 (4)	-0.0008 (4)
O11	0.0311 (18)	0.0310 (19)	0.0233 (17)	0.0030 (15)	0.0018 (15)	-0.0022 (14)
O12	0.0254 (17)	0.0273 (19)	0.035 (2)	-0.0023 (14)	-0.0061 (15)	0.0017 (15)

C13	0.024 (2)	0.021 (2)	0.031 (3)	0.0038 (18)	-0.0037 (19)	-0.0047 (18)
C14	0.037 (3)	0.026 (3)	0.036 (3)	0.001 (2)	0.010 (2)	-0.002 (2)
C15	0.045 (3)	0.030 (3)	0.039 (3)	0.009 (2)	0.007 (3)	-0.004 (2)
C16	0.057 (4)	0.021 (3)	0.035 (3)	0.008 (3)	-0.010 (3)	-0.003 (2)
C17	0.085 (5)	0.027 (3)	0.064 (5)	0.004 (3)	-0.011 (4)	-0.011 (3)
C18	0.045 (3)	0.024 (3)	0.040 (3)	-0.008 (2)	-0.002 (3)	0.002 (2)
C19	0.032 (2)	0.031 (2)	0.030 (2)	-0.0048 (19)	0.000 (3)	0.000 (3)
C20	0.043 (3)	0.021 (3)	0.082 (5)	0.000 (2)	0.000 (3)	0.003 (3)
O21	0.069 (3)	0.024 (2)	0.057 (3)	0.008 (2)	-0.009 (2)	-0.025 (2)
O22	0.055 (2)	0.0177 (16)	0.087 (3)	0.0075 (15)	-0.016 (3)	0.011 (3)
O23	0.036 (2)	0.0284 (18)	0.083 (4)	-0.0171 (15)	-0.006 (3)	0.009 (2)

Geometric parameters (Å, º) for (I)

Se1—C2	1.953 (5)	S10—C13	1.767 (5)
Se1—C20	1.966 (6)	C13—C14	1.396 (7)
C2—C3	1.487 (6)	C13—C19	1.367 (7)
C2—C9	1.313 (6)	C14—C15	1.378 (7)
C3—C4	1.397 (8)	C14—H141	0.920
C3—C8	1.382 (6)	C15—C16	1.388 (9)
C4—C5	1.389 (6)	C15—H151	0.933
C4—H41	0.936	C16—C17	1.508 (8)
C5—C6	1.390 (8)	C16—C18	1.385 (8)
C5—H51	0.953	C17—H171	0.959
C6—C7	1.379 (7)	C17—H172	0.970
C6—H61	0.934	C17—H173	0.968
C7—C8	1.389 (7)	C18—C19	1.402 (8)
C7—H71	0.940	C18—H181	0.927
C8—H81	0.927	C19—H191	0.929
C9—S10	1.771 (5)	C20—O21	1.294 (9)
C9—H91	0.948	C20—O22	1.324 (8)
S10—O11	1.433 (4)	C20—O23	1.344 (7)
S10—O12	1.439 (4)		
C2—Se1—C20	97.4 (3)	S10—C13—C14	118.1 (4)
Se1—C2—C3	116.6 (3)	S10—C13—C19	120.7 (4)
Se1—C2—C9	112.0 (4)	C14—C13—C19	121.2 (5)
C3—C2—C9	131.2 (4)	C13—C14—C15	118.7 (5)
C2—C3—C4	120.0 (4)	C13—C14—H141	120.5

C2—C3—C8	120.5 (4)	C15—C14—H141	120.8
C4—C3—C8	119.4 (4)	C14—C15—C16	121.5 (6)
C3—C4—C5	119.7 (6)	C14—C15—H151	119.2
C3—C4—H41	119.0	C16—C15—H151	119.3
C5—C4—H41	121.2	C15—C16—C17	121.1 (6)
C4—C5—C6	120.3 (6)	C15—C16—C18	118.7 (5)
C4—C5—H51	120.0	C17—C16—C18	120.2 (6)
C6—C5—H51	119.7	C16—C17—H171	109.0
C5—C6—C7	119.8 (5)	C16—C17—H172	109.0
C5—C6—H61	120.3	H171—C17—H172	110.0
C7—C6—H61	119.9	C16—C17—H173	108.2
C6—C7—C8	120.0 (5)	H171—C17—H173	110.8
C6—C7—H71	118.4	H172—C17—H173	109.7
C8—C7—H71	121.6	C16—C18—C19	120.6 (6)
C7—C8—C3	120.7 (5)	C16—C18—H181	119.6
C7—C8—H81	119.7	C19—C18—H181	119.7
C3—C8—H81	119.7	C18—C19—C13	119.2 (6)
C2—C9—S10	125.6 (4)	C18—C19—H191	120.2
C2—C9—H91	118.4	C13—C19—H191	120.6
S10—C9—H91	116.1	Se1—C20—O21	112.8 (4)
C9—S10—O11	106.4 (2)	Se1—C20—O22	108.0 (5)
C9—S10—O12	111.1 (2)	O21—C20—O22	109.6 (6)
O11—S10—O12	119.3 (2)	Se1—C20—O23	112.2 (4)
C9—S10—C13	101.8 (2)	O21—C20—O23	106.5 (6)
O11—S10—C13	108.5 (2)	O22—C20—O23	107.7 (6)
O12—S10—C13	108.3 (2)		

Hydrogen-bond geometry (Å, º) for (I)

D—H···*A*	*D*—H	H···*A*	*D*···*A*	*D*—H···*A*
C8—H81···O12^{i}	0.93	2.51	3.428 (8)	172
C9—H91···O11^{ii}	0.95	2.44	3.332 (8)	157

Symmetry codes: (i) *x*, *y*, *z*+1; (ii) -*x*+3/2, *y*, *z*+1/2.

Document origin: *publCIF* [Westrip, S. P. (2010). *J. Apply. Cryst.*, **43**, 920-925].

NMR and GC–MS spectra

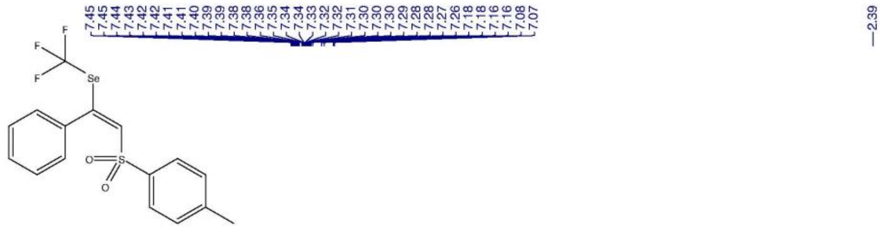

3a

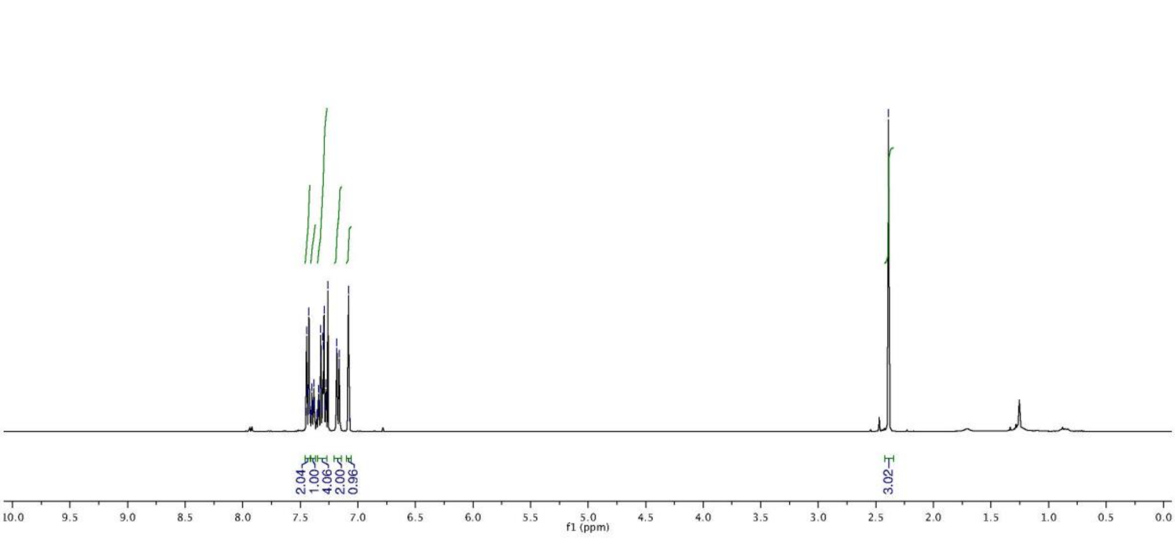
7.45 7.45 7.44 7.43 7.42 7.42 7.41 7.40 7.39 7.39 7.38 7.38 7.36 7.35 7.34 7.34 7.33 7.32 7.32 7.31 7.30 7.30 7.29 7.28 7.28 7.27 7.26 7.18 7.16 7.16 7.08 7.07
2.39
2.04 1.00 4.06 2.00 0.96
3.02
10.0 9.5 9.0 8.5 8.0 7.5 7.0 6.5 6.0 5.5 5.0 4.5 4.0 3.5 3.0 2.5 2.0 1.5 1.0 0.5 0.0
f1 (ppm)

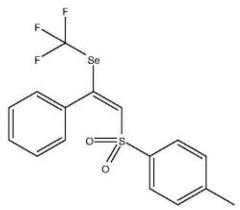

3a

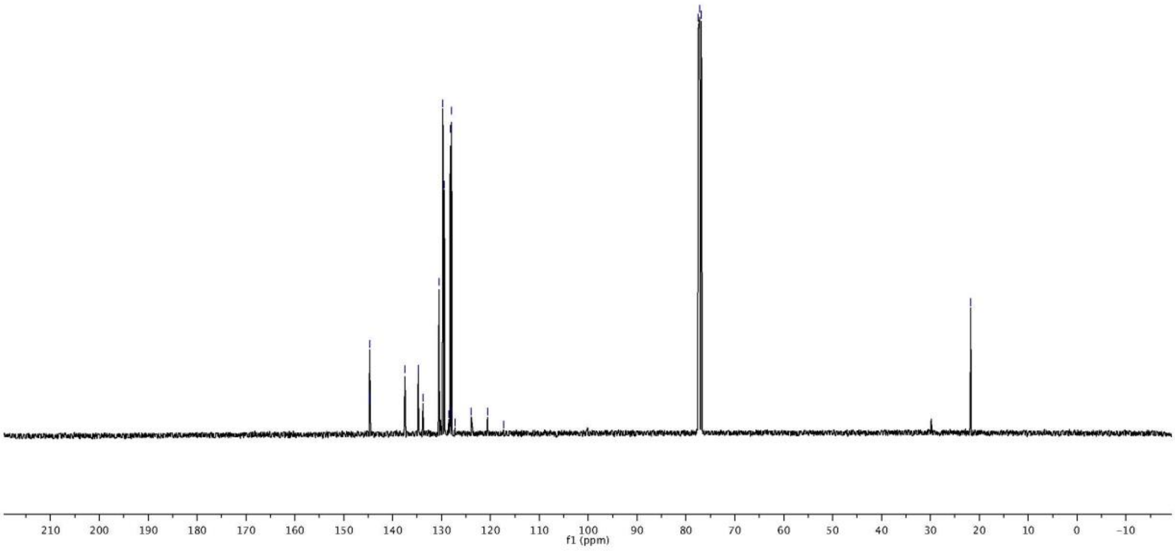
144.73 144.72 144.70 137.48 134.77 134.75 133.79 130.52 129.72 129.42 128.51 128.22 127.93 127.86 127.24 123.92 120.59 117.27
77.48 77.16 76.84
21.75
210 200 190 180 170 160 150 140 130 120 110 100 90 80 70 60 50 40 30 20 10 0 -10
f1 (ppm)

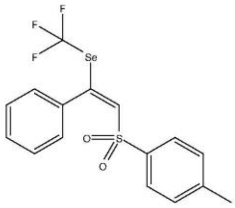
F
F
F
Se
O
S
O

3a

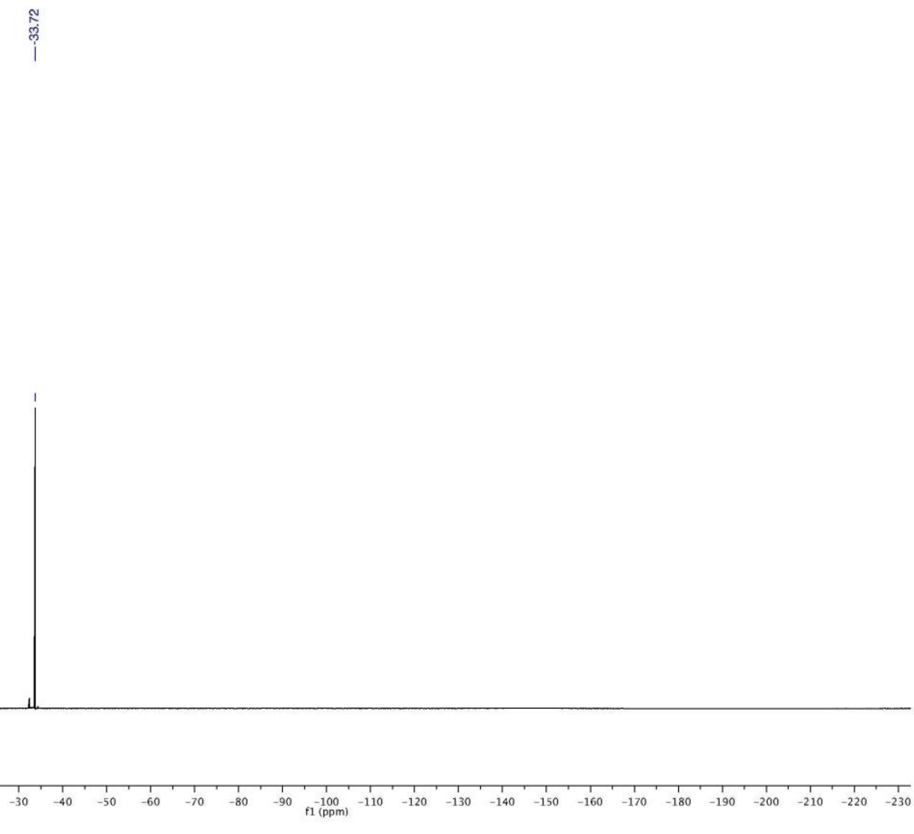
-33.72
30 20 10 0 -10 -20 -30 -40 -50 -60 -70 -80 -90 -100 -110 -120 -130 -140 -150 -160 -170 -180 -190 -200 -210 -220 -230
f1 (ppm)

File   :C:\msdchem\data\anis\CG261CARAC.D
Operator   :
Acquired   : 30 May 2017 15:06   using AcqMethod ANIS.M
Instrument :   GCMS
Sample Name: CG261CARAC
Misc Info   :
Vial Number: 2

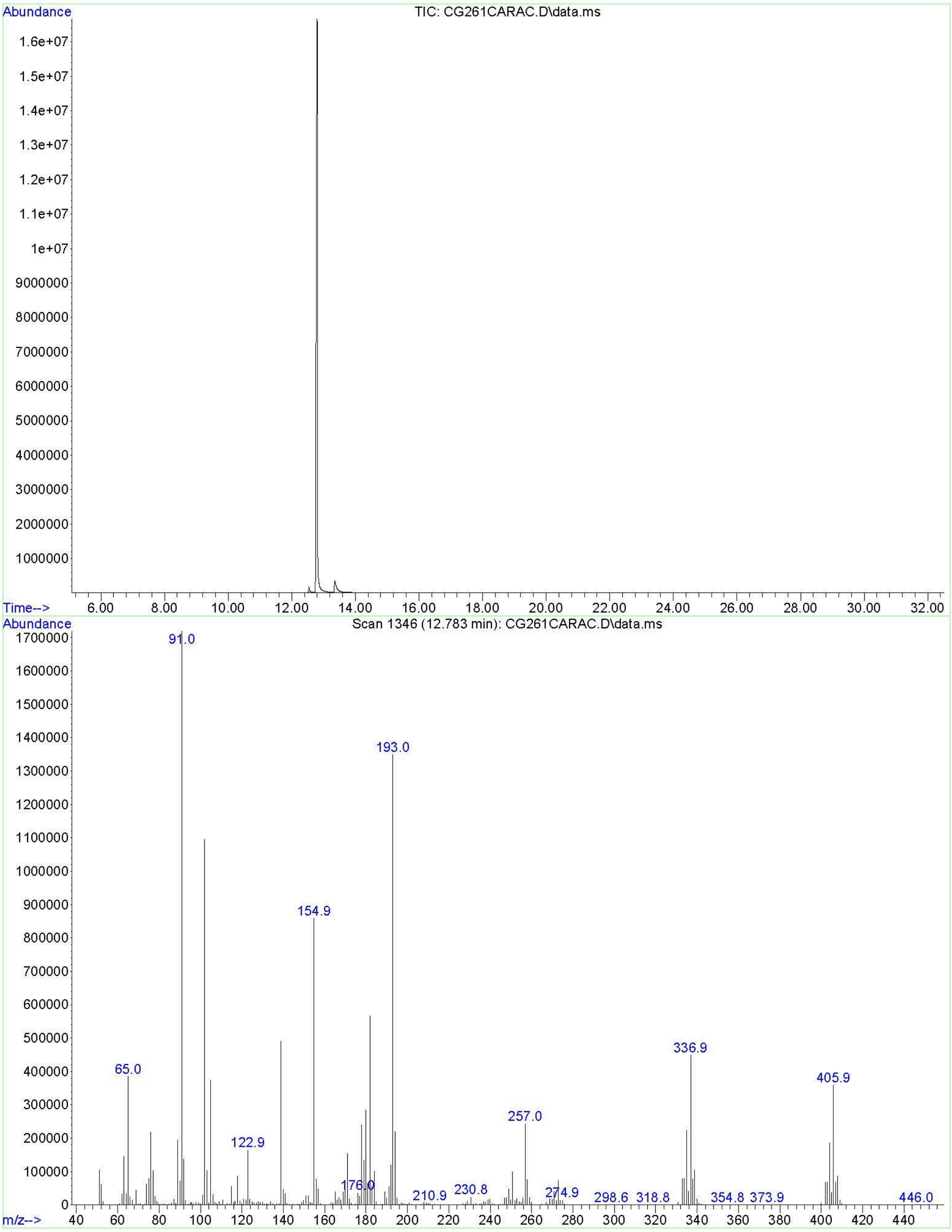

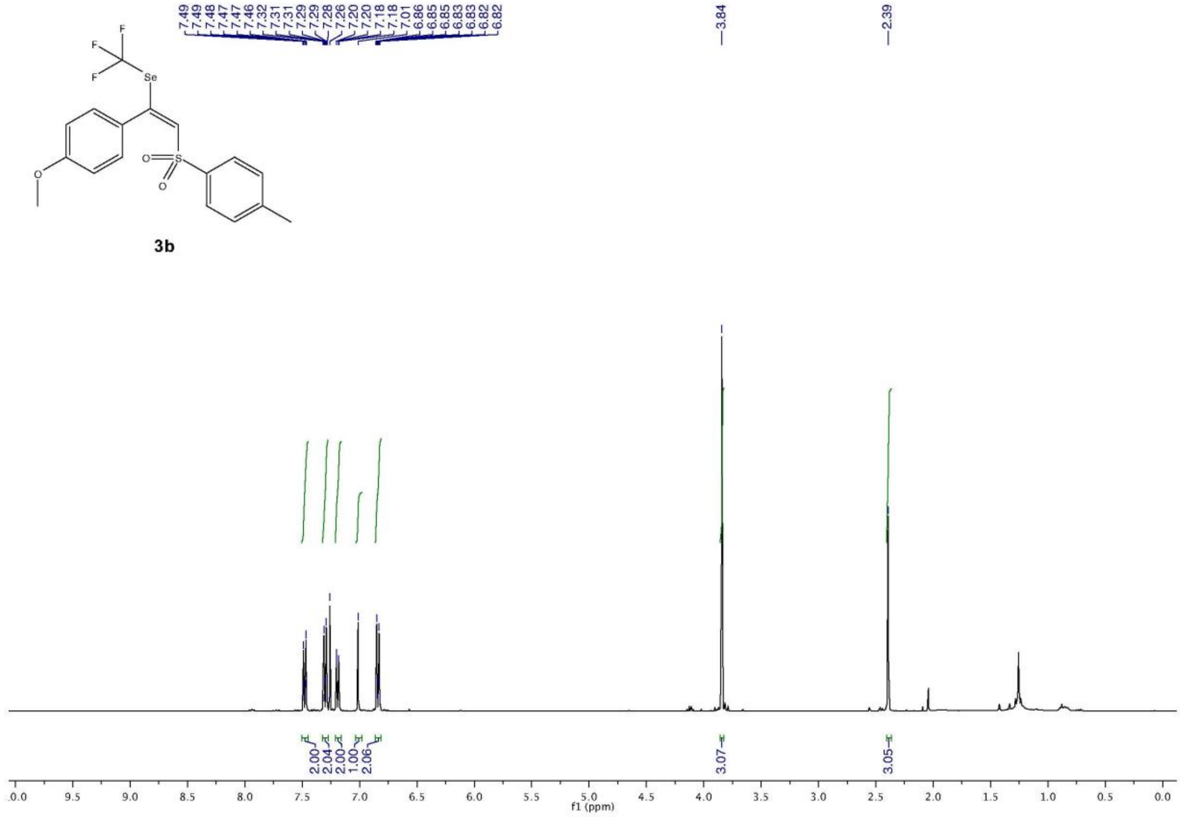

Se
F
O
S
3b
7.49
7.49
7.48
7.47
7.46
7.32
7.31
7.29
7.29
7.28
7.26
7.20
7.20
7.18
7.01
6.86
6.85
6.83
6.83
6.82
3.84
2.39
2.00
2.04
2.00
1.00
2.06
3.07
3.05
f1 (ppm)

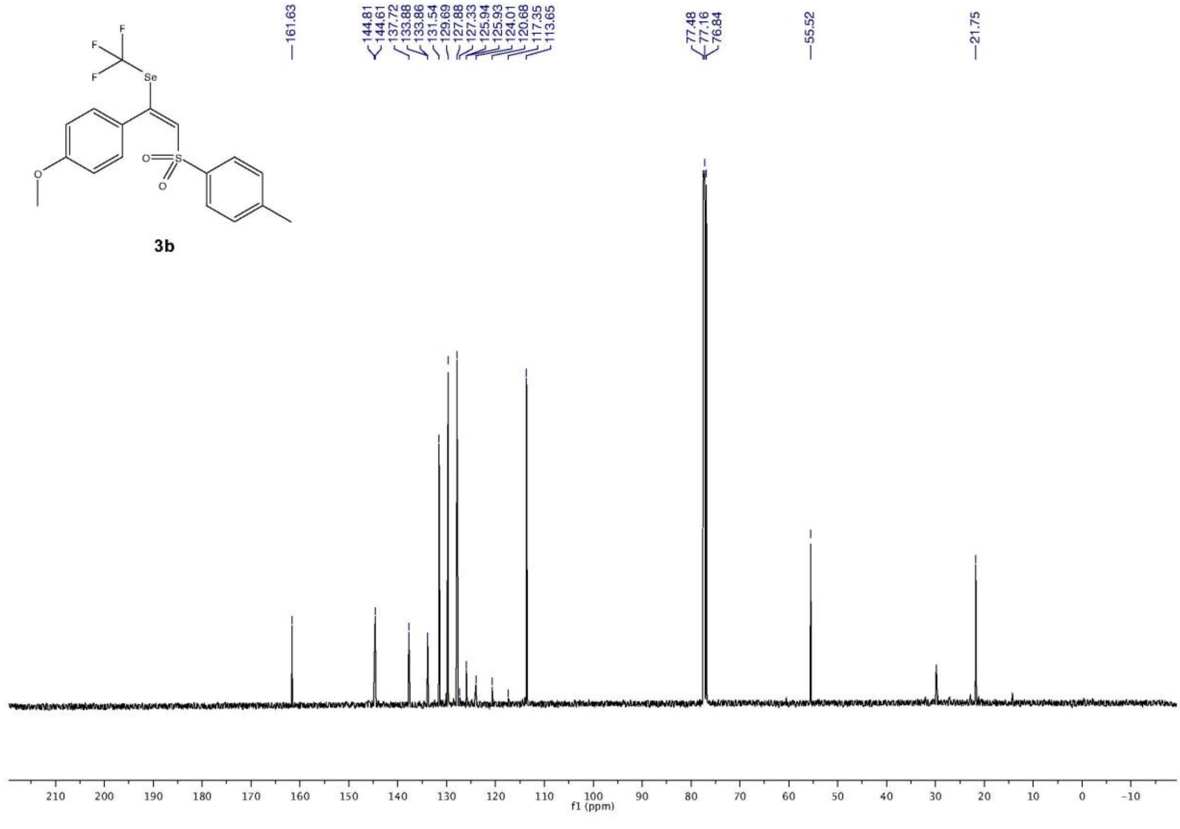

Se
F
O
S
3b
161.63
144.81
144.61
137.72
133.88
133.86
131.54
129.69
127.88
127.33
125.94
125.93
124.01
120.68
117.35
113.65
77.48
77.16
76.84
55.52
21.75
f1 (ppm)

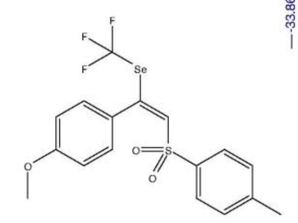

3b

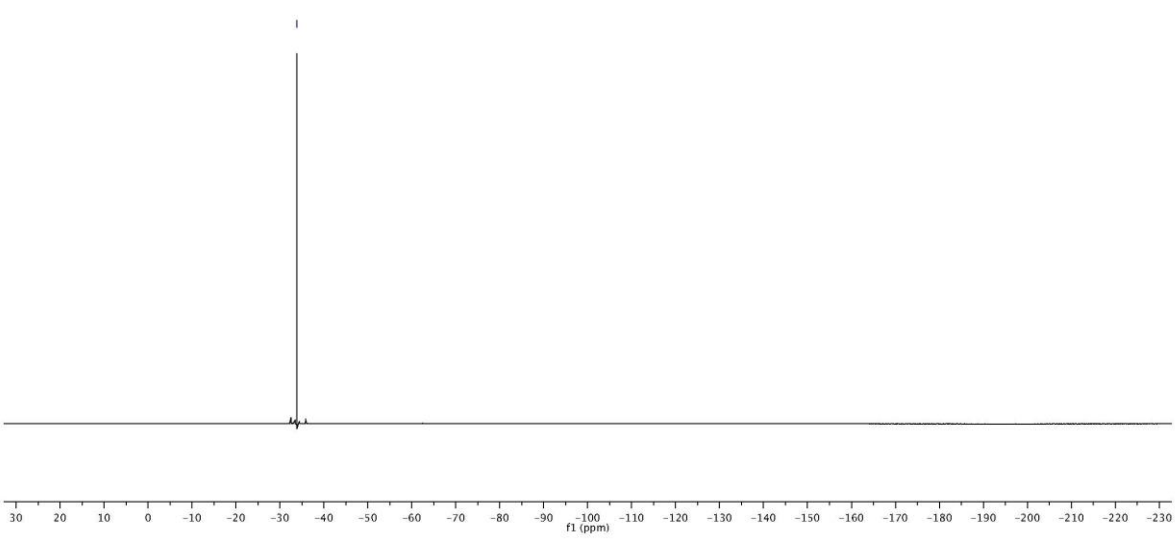

-33.86
30
20
10
0
−10
−20
−30
−40
−50
−60
−70
−80
−90
−100
−110
−120
−130
−140
−150
−160
−170
−180
−190
−200
−210
−220
−230
f1 (ppm)

```
File        :C:\msdchem\data\anis\TD80CARAC.D
Operator    :
Acquired    : 30 May 2017  15:47      using AcqMethod ANIS.M
Instrument  :    GCMS
Sample Name: TD80CARAC
Misc Info   :
Vial Number: 2
```

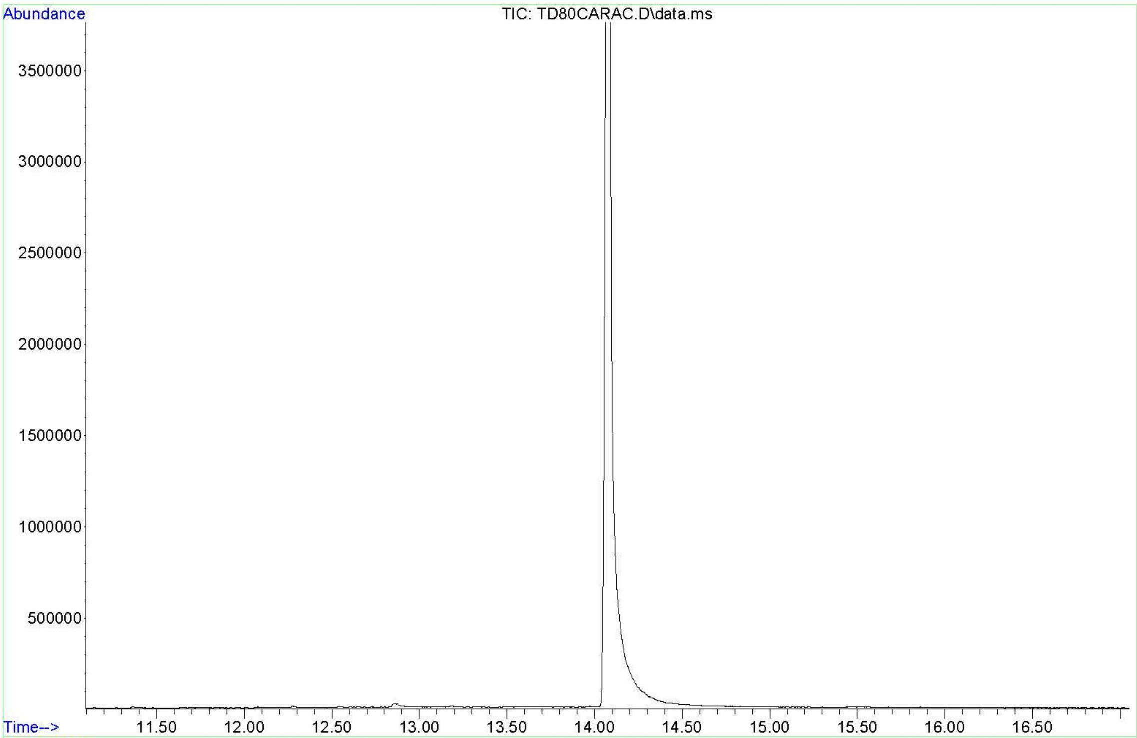

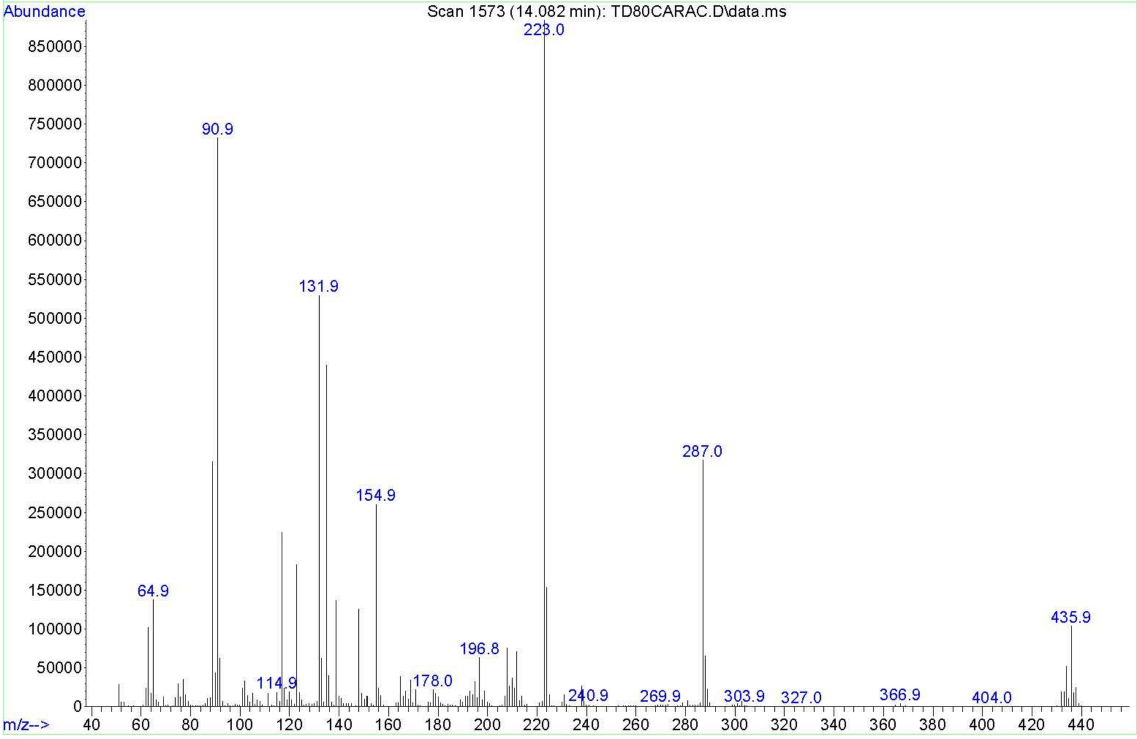

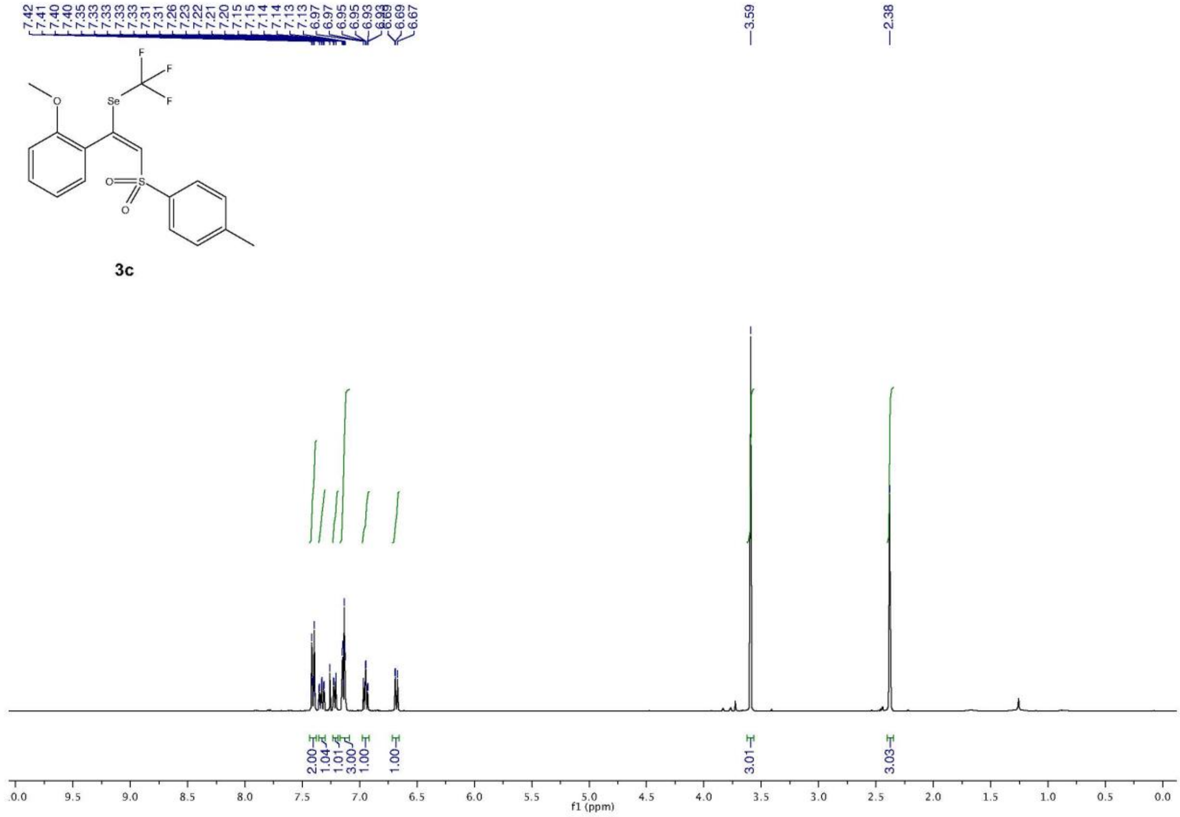
7.42
7.41
7.40
7.40
7.35
7.33
7.33
7.33
7.33
7.31
7.31
7.26
7.23
7.22
7.21
7.20
7.15
7.15
7.14
7.14
7.13
7.13
6.97
6.97
6.95
6.95
6.93
6.69
6.67
3.59
2.38
Se
F
F
F
O
O
S
O
3c
2.00
1.04
1.01
3.00
1.00
1.00
3.01
3.03
f1 (ppm)

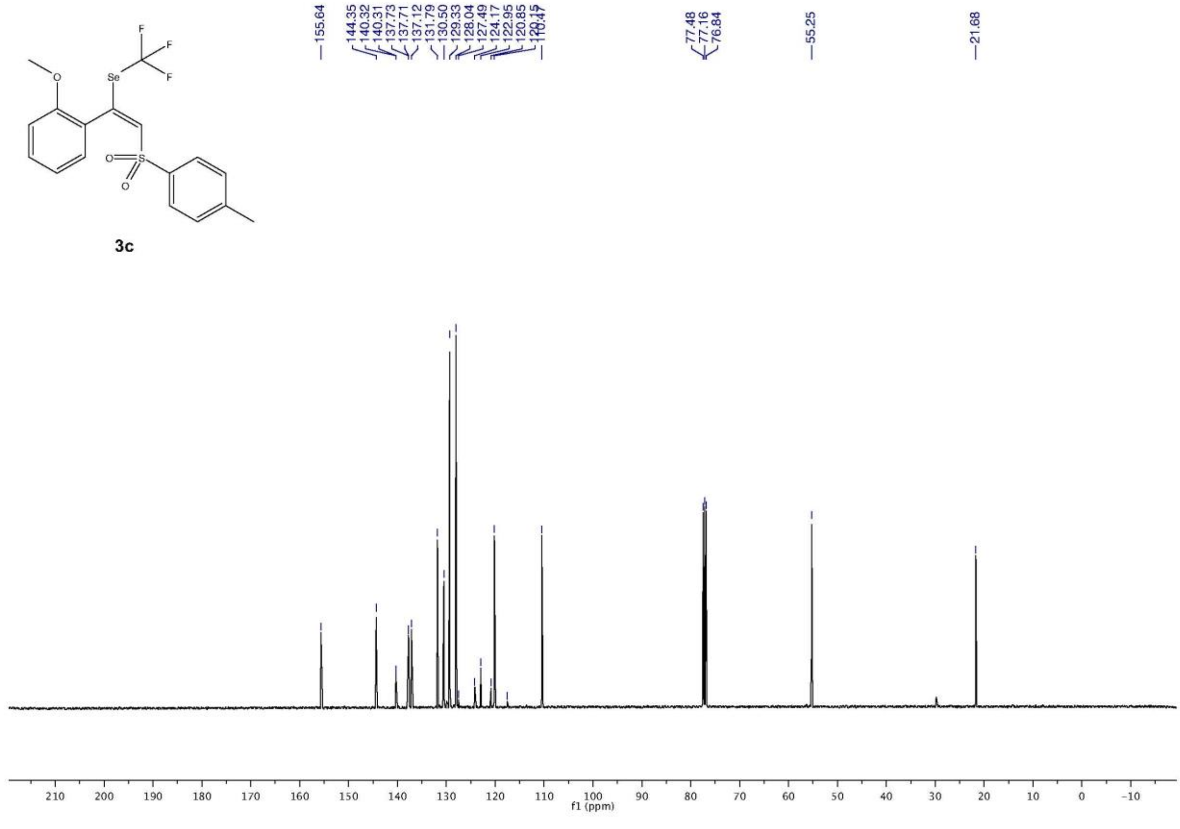
155.64
144.35
140.32
140.31
137.73
137.71
137.12
131.79
130.50
129.33
128.04
127.49
124.17
122.95
120.85
110.47
77.48
77.16
76.84
55.25
21.68
Se
F
F
F
O
O
S
O
3c
f1 (ppm)

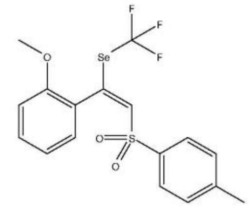

3c

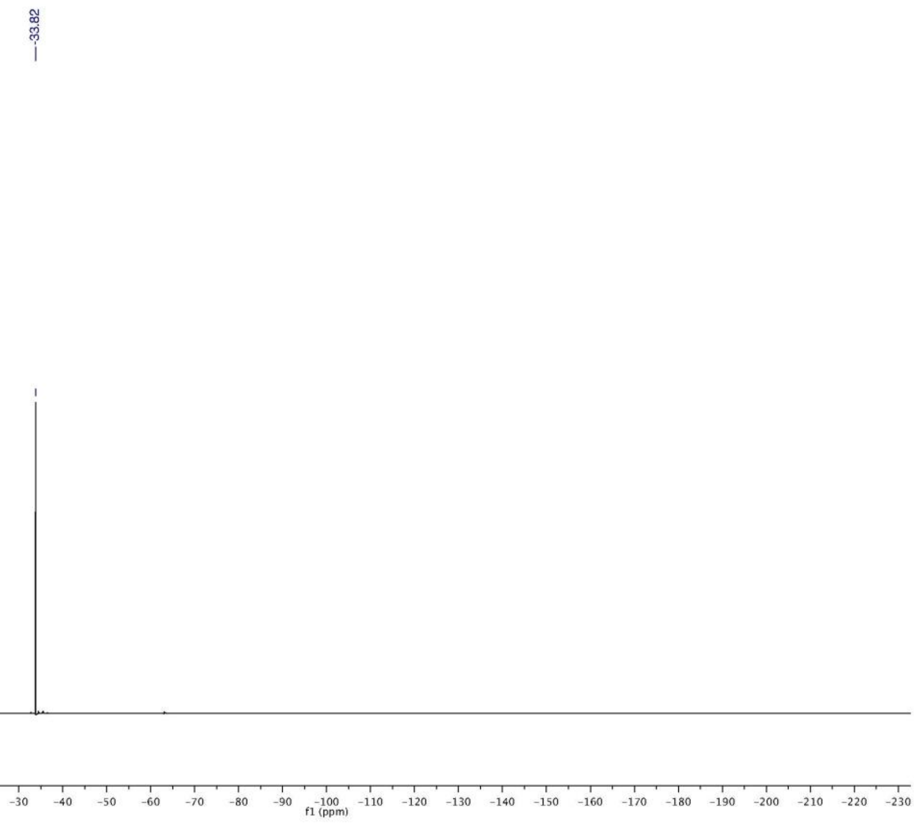

-33.82
30
20
10
0
-10
-20
-30
-40
-50
-60
-70
-80
-90
-100
-110
-120
-130
-140
-150
-160
-170
-180
-190
-200
-210
-220
-230
f1 (ppm)

```
File        :C:\msdchem\data\anis\CG319CARAC.D
Operator    :
Acquired    : 31 May 2017  17:25     using AcqMethod ANIS.M
Instrument  :    GCMS
Sample Name: CG319CARAC
Misc Info   :
Vial Number: 2
```

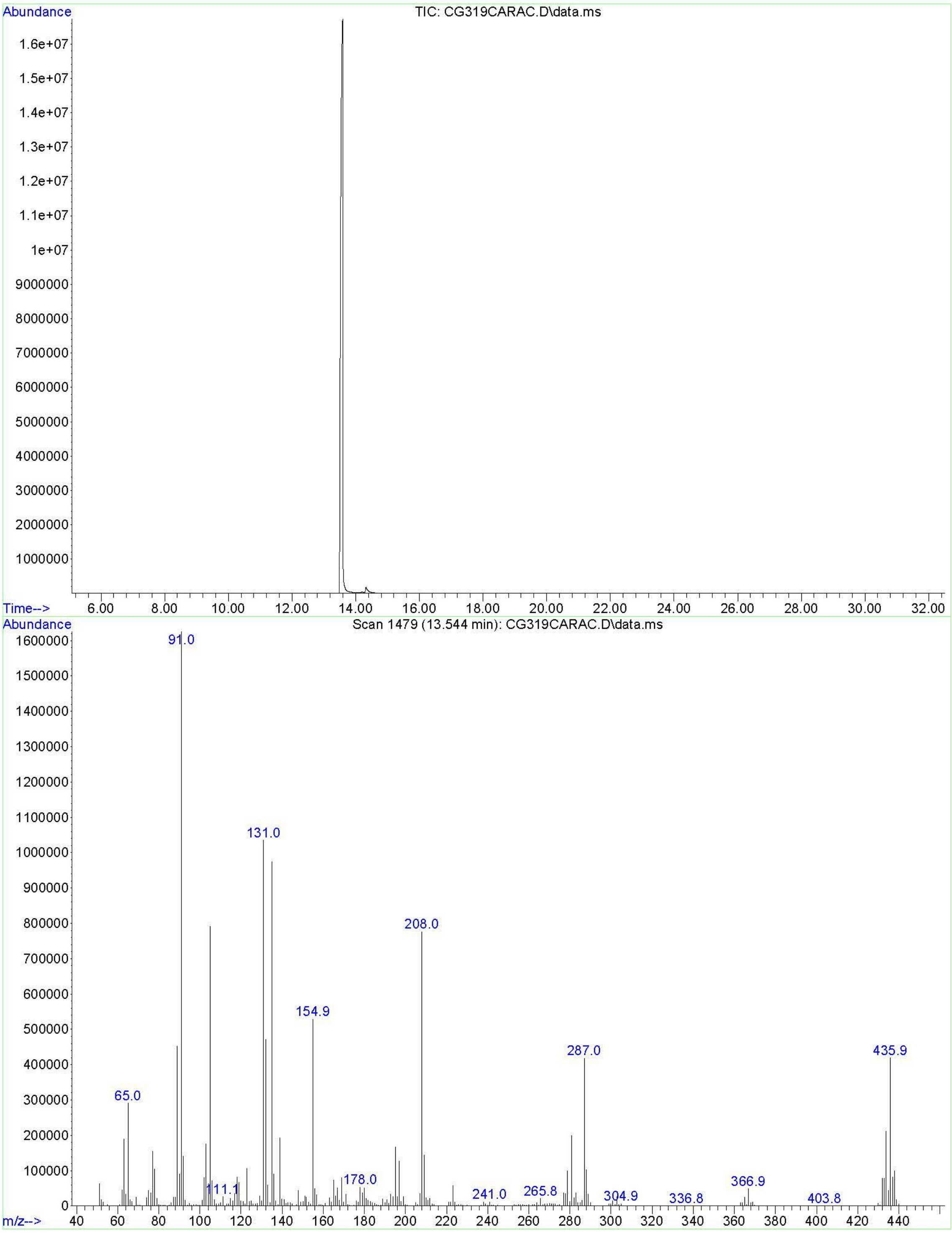

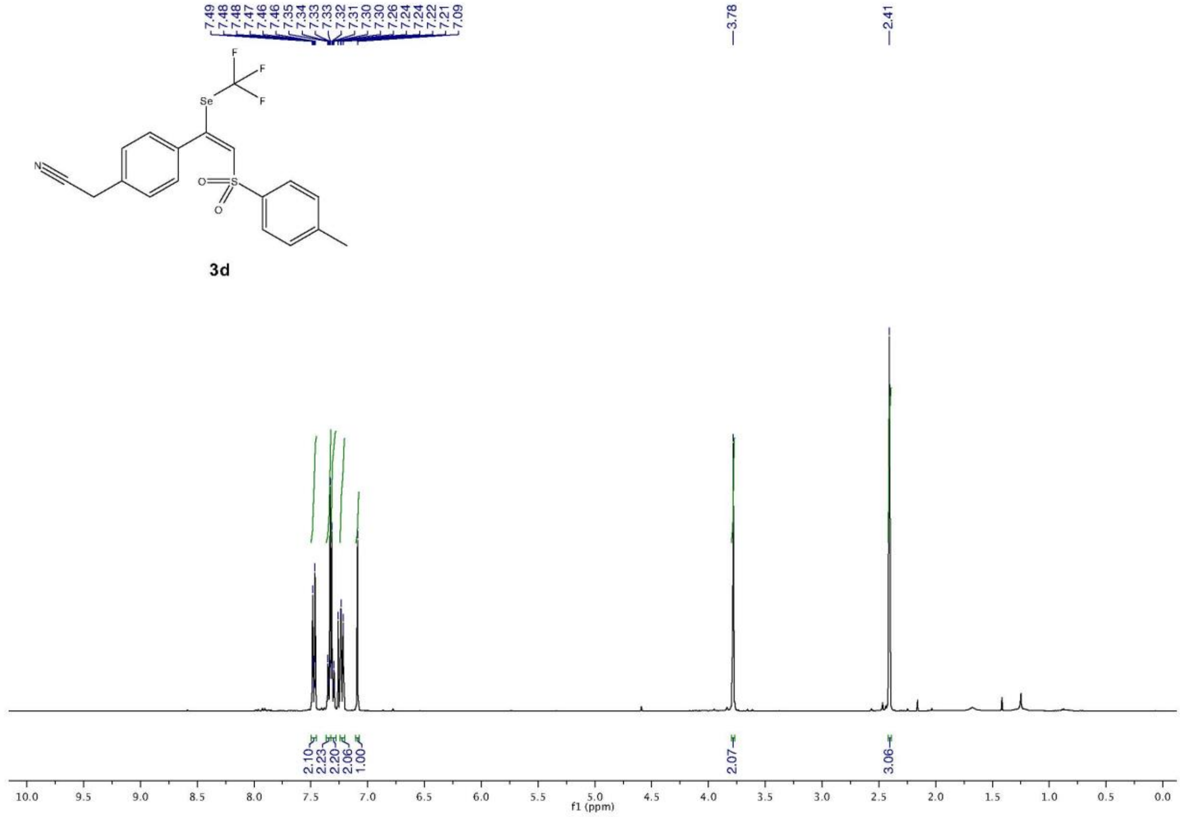
7.49 7.48 7.48 7.47 7.46 7.46 7.35 7.34 7.33 7.33 7.32 7.31 7.30 7.30 7.26 7.24 7.24 7.22 7.21 7.09
3.78
2.41
3d
2.10 2.23 2.20 2.06 1.00
2.07
3.06
f1 (ppm)

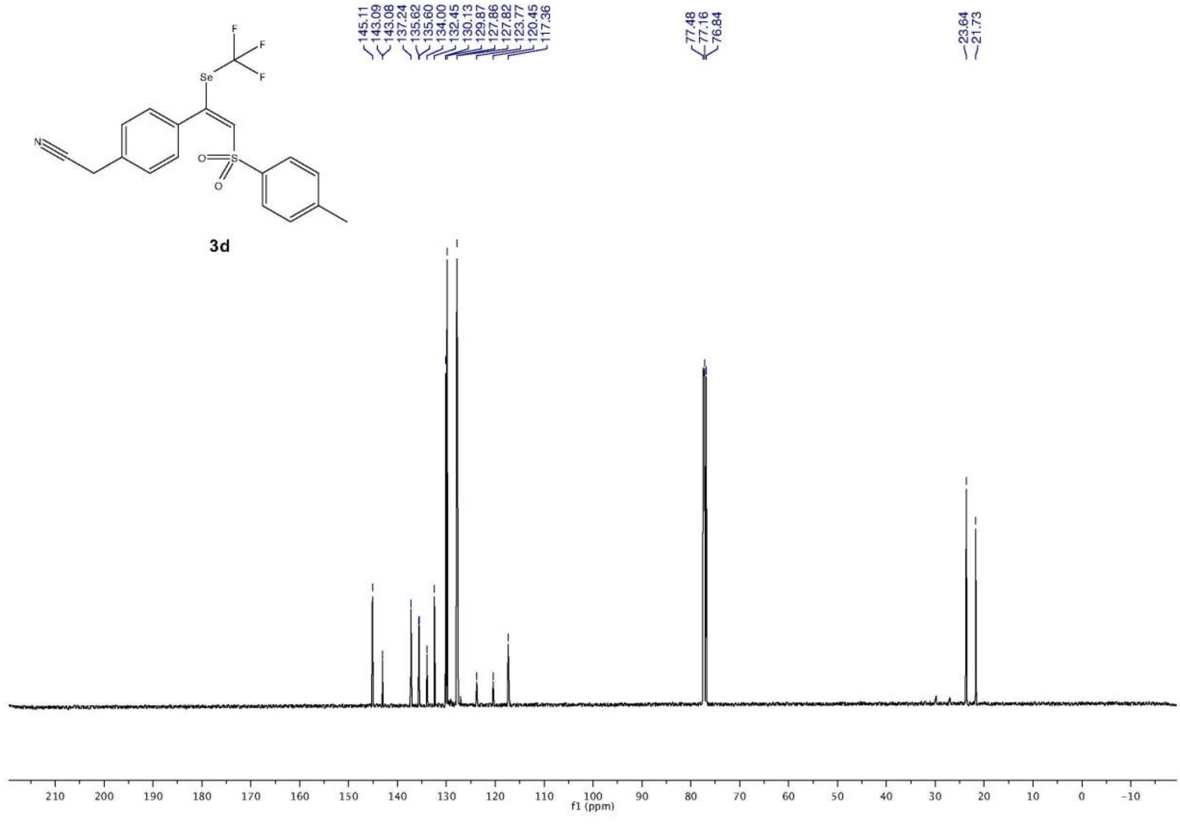
145.11 143.09 143.08 137.24 135.62 135.60 134.00 132.45 130.13 129.87 127.86 127.82 123.77 120.45 117.36
77.48 77.16 76.84
23.64 21.73
3d
f1 (ppm)

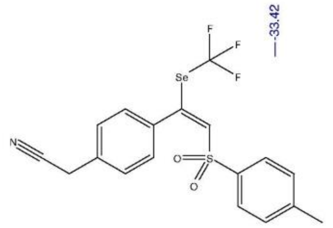
F
F
F
Se
N
O
S
O

3d

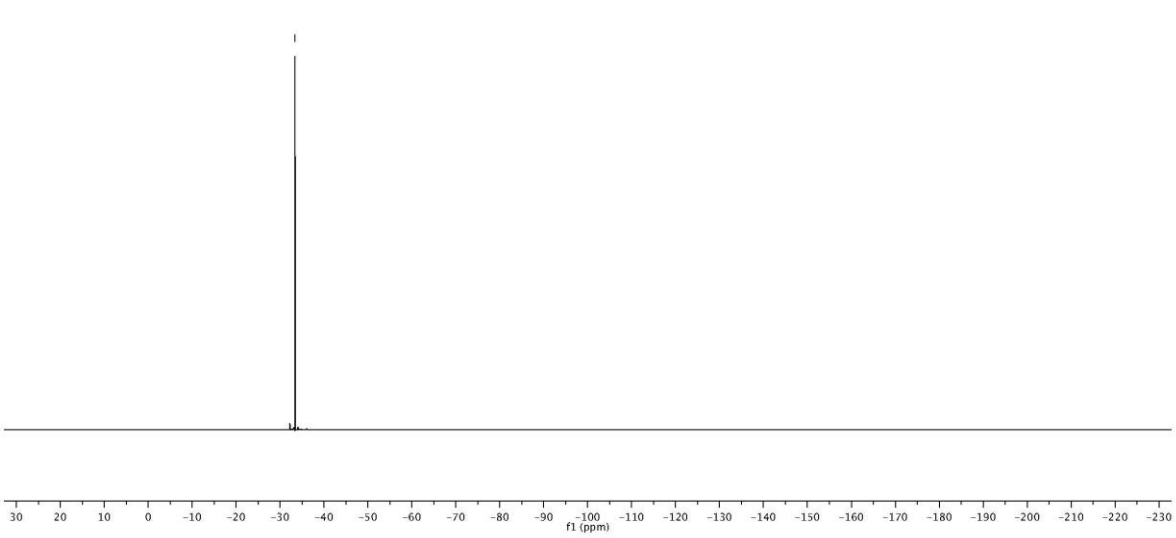
-33.42
30
20
10
0
−10
−20
−30
−40
−50
−60
−70
−80
−90
−100
−110
−120
−130
−140
−150
−160
−170
−180
−190
−200
−210
−220
−230
f1 (ppm)

```
File        :C:\msdchem\data\anis\CGTD94CARAC.D
Operator    :
Acquired    : 21 Jul 2017  14:23      using AcqMethod anis.M
Instrument  :    GCMS
Sample Name: cgtd94carac
Misc Info   :
Vial Number: 2
```

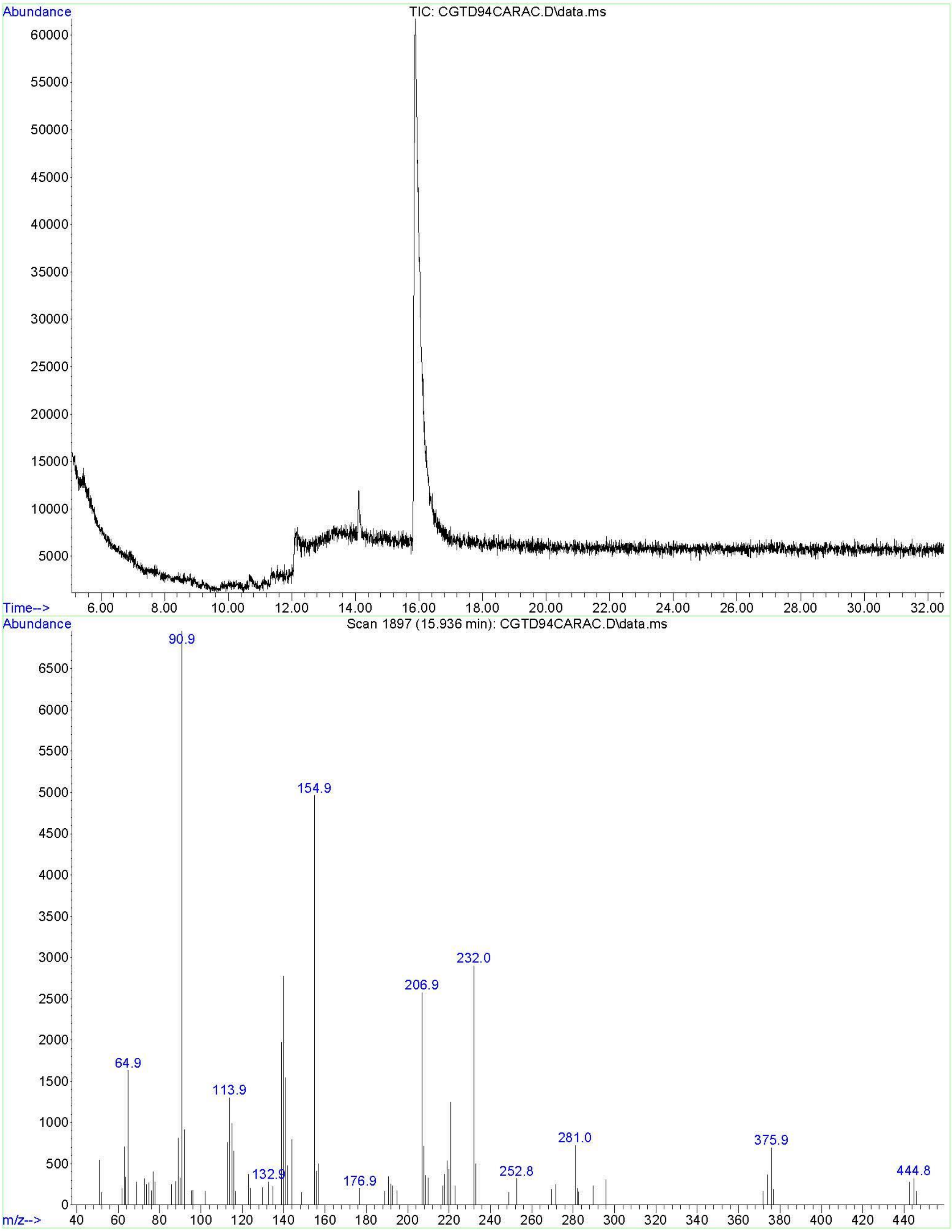

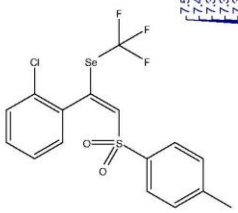
Cl
Se
F
F
F
O
S
O

3e

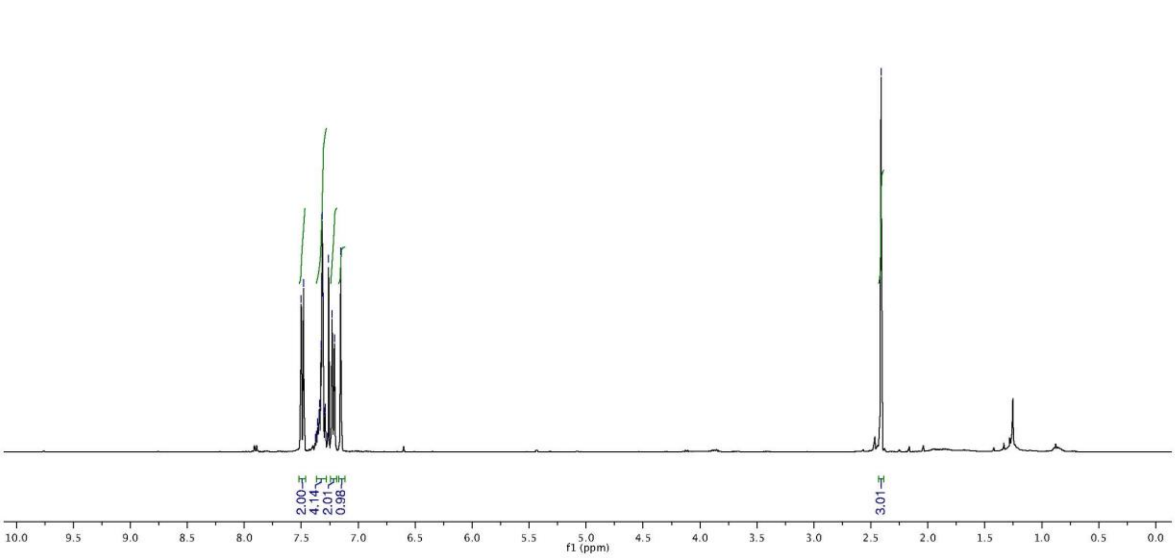
7.50
7.48
7.38
7.37
7.36
7.35
7.34
7.34
7.33
7.32
7.31
7.31
7.29
7.29
7.27
7.26
7.23
7.21
7.15
2.41
2.00
4.14
2.01
0.98
3.01
10.0 9.5 9.0 8.5 8.0 7.5 7.0 6.5 6.0 5.5 5.0 4.5 4.0 3.5 3.0 2.5 2.0 1.5 1.0 0.5 0.0
f1 (ppm)

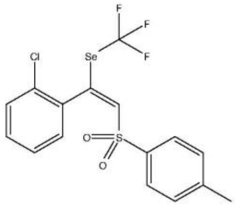
Cl
Se
F
F
F
O
S
O

3e

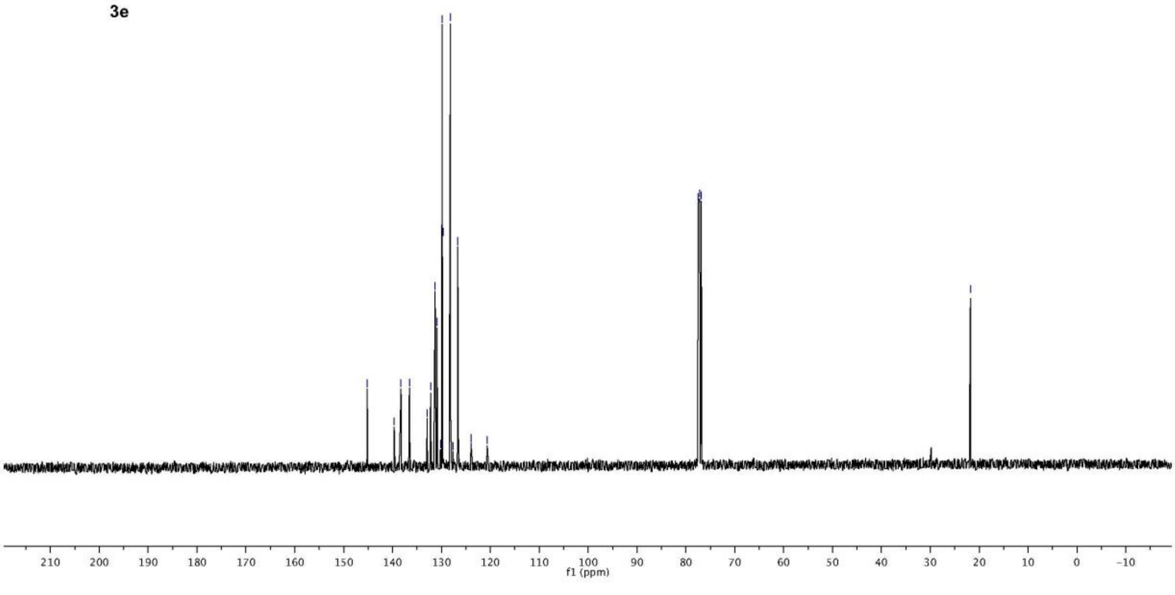
145.17
139.65
138.27
136.56
132.93
132.22
131.30
130.91
130.22
129.86
129.68
128.17
127.62
126.66
123.96
120.64
77.48
77.16
76.84
21.79
210 200 190 180 170 160 150 140 130 120 110 100 90 80 70 60 50 40 30 20 10 0 -10
f1 (ppm)

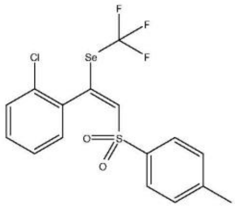

Cl
Se
F
F
F
O
S
O

3e

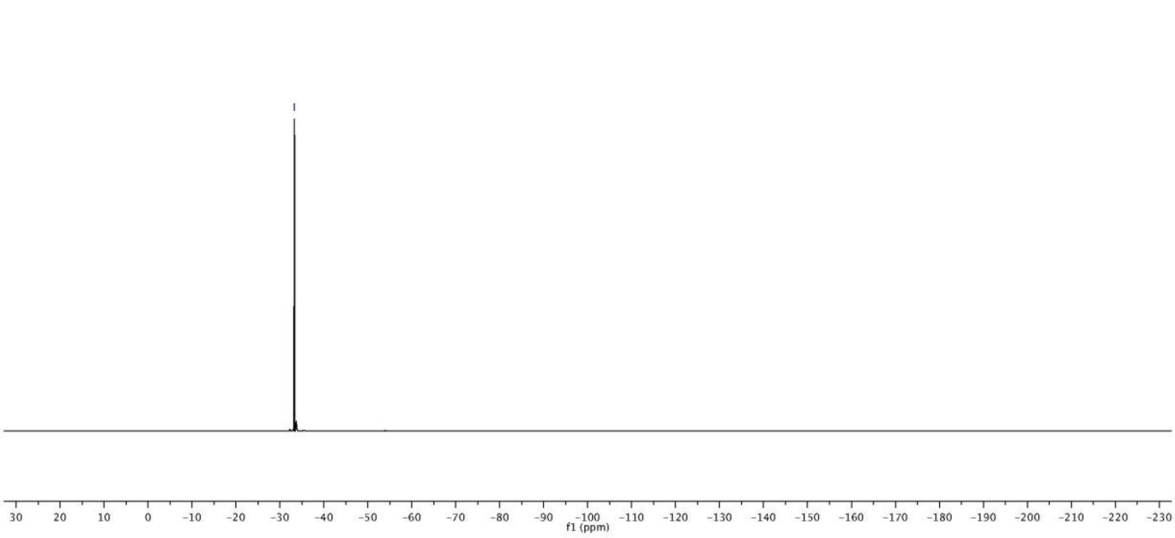

-33.33
30 20 10 0 -10 -20 -30 -40 -50 -60 -70 -80 -90 -100 -110 -120 -130 -140 -150 -160 -170 -180 -190 -200 -210 -220 -230
f1 (ppm)

```
File        :C:\msdchem\data\anis\CGTD114CARAC.D
Operator    :
Acquired    : 24 Jul 2017  10:01     using AcqMethod anis.M
Instrument  :    GCMS
Sample Name: cgtd114carac
Misc Info   :
Vial Number: 2
```

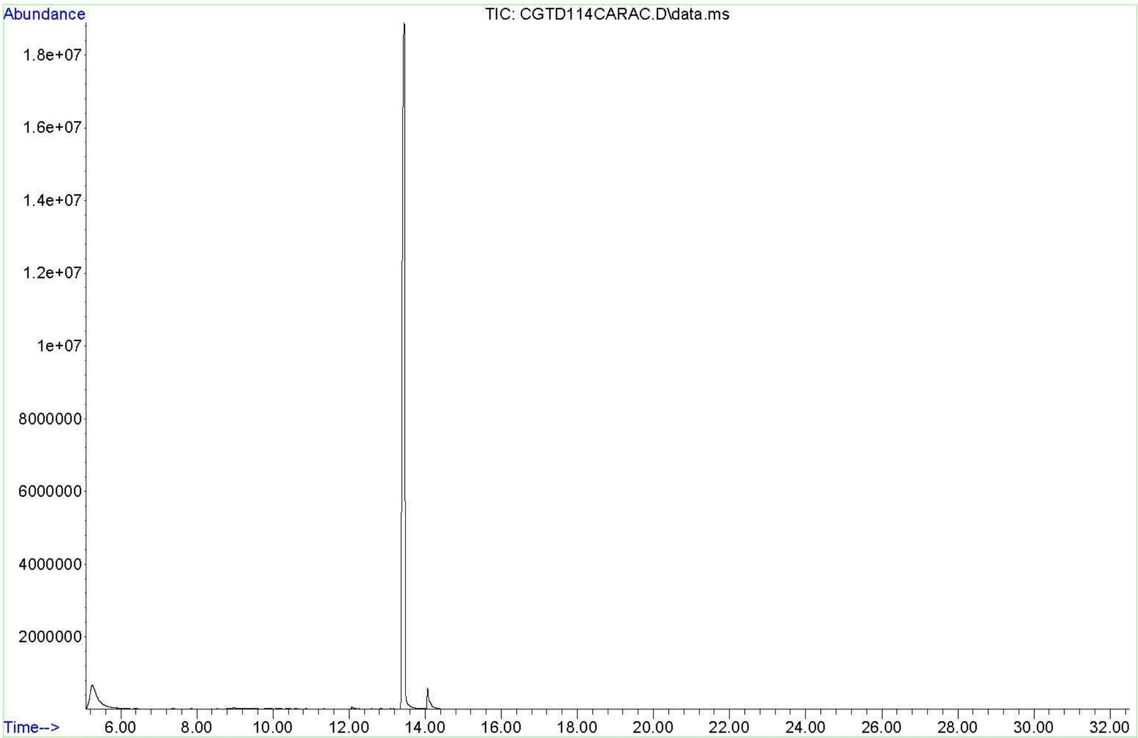

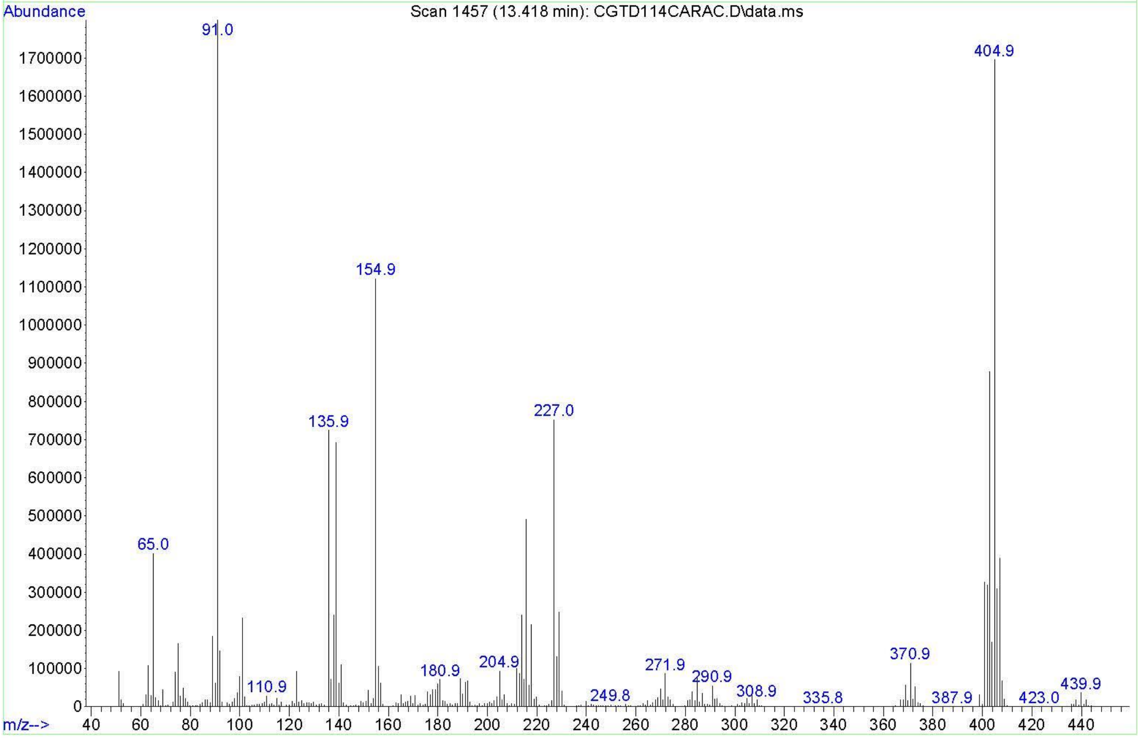

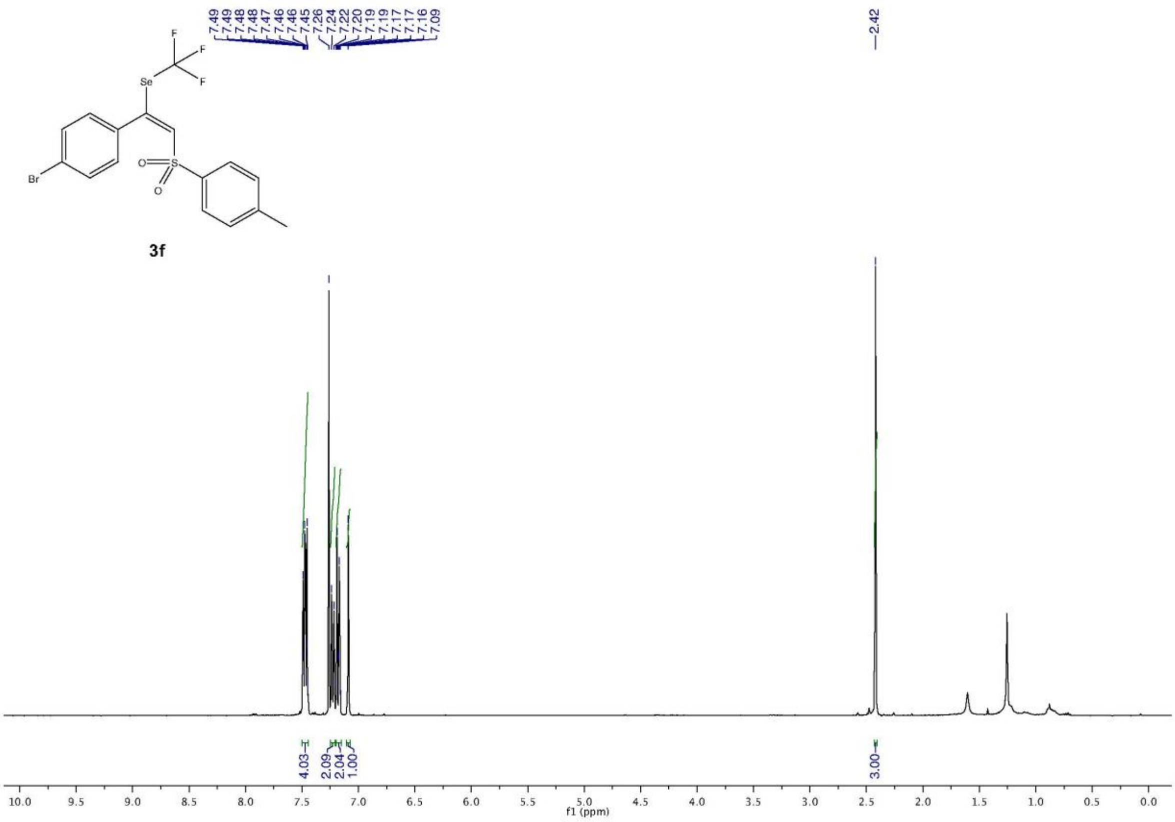
7.49
7.49
7.48
7.48
7.47
7.46
7.46
7.45
7.26
7.24
7.22
7.20
7.19
7.19
7.17
7.16
7.09
2.42
Se
F
F
F
O
S
O
Br
3f
4.03
2.09
2.04
1.00
3.00
f1 (ppm)

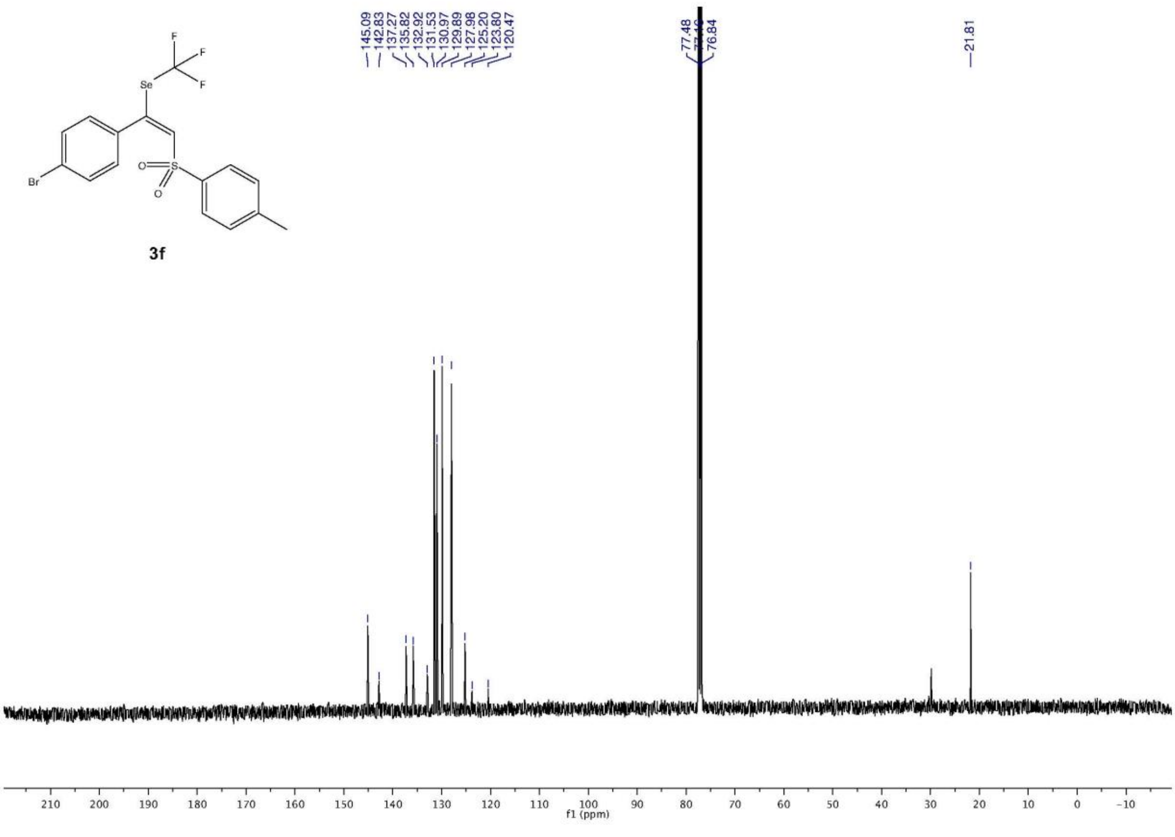
145.09
142.83
137.27
135.82
132.92
131.53
130.97
129.89
127.98
125.20
123.80
120.47
77.48
76.84
21.81
Se
F
F
F
O
S
O
Br
3f
f1 (ppm)

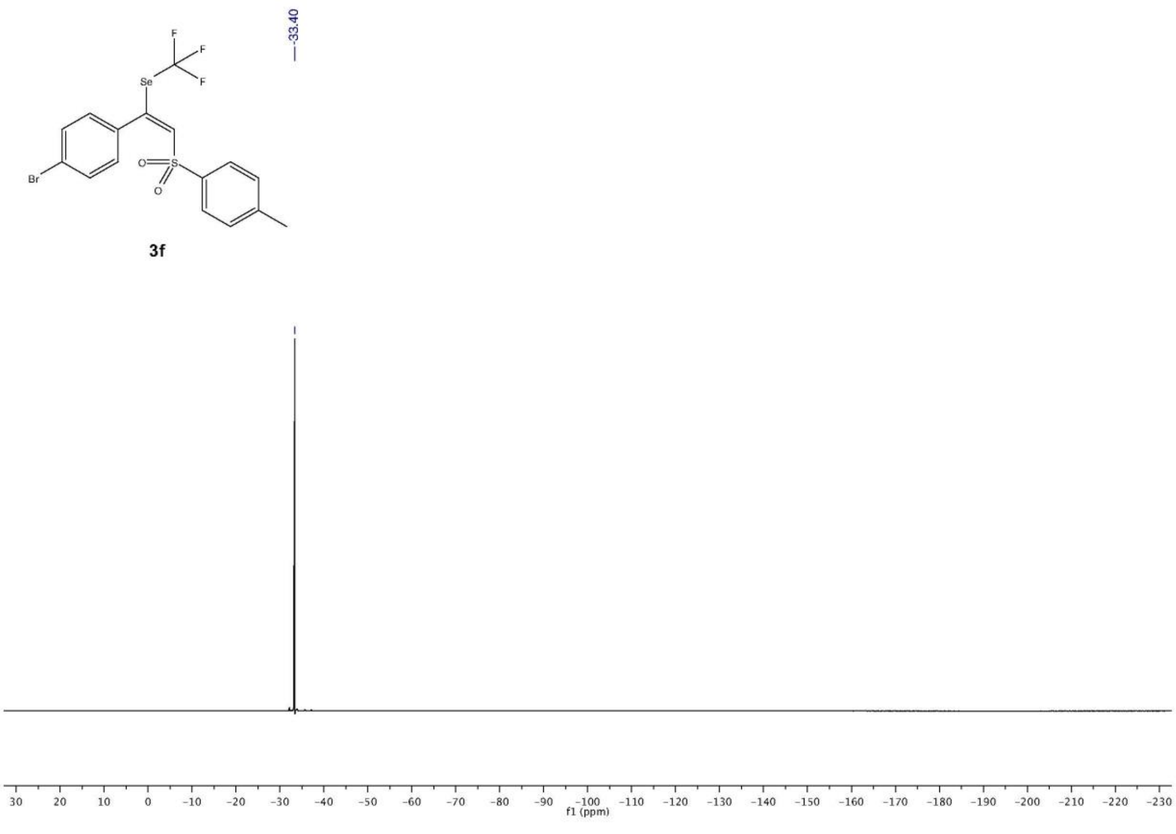
3f
−33.40
f1 (ppm)

```
File        :C:\msdchem\data\anis\CGTD79CARAC.D
Operator    :
Acquired    : 30 May 2017  17:55      using AcqMethod ANIS.M
Instrument  :    GCMS
Sample Name: CGTD79CARAC
Misc Info   :
Vial Number: 2
```

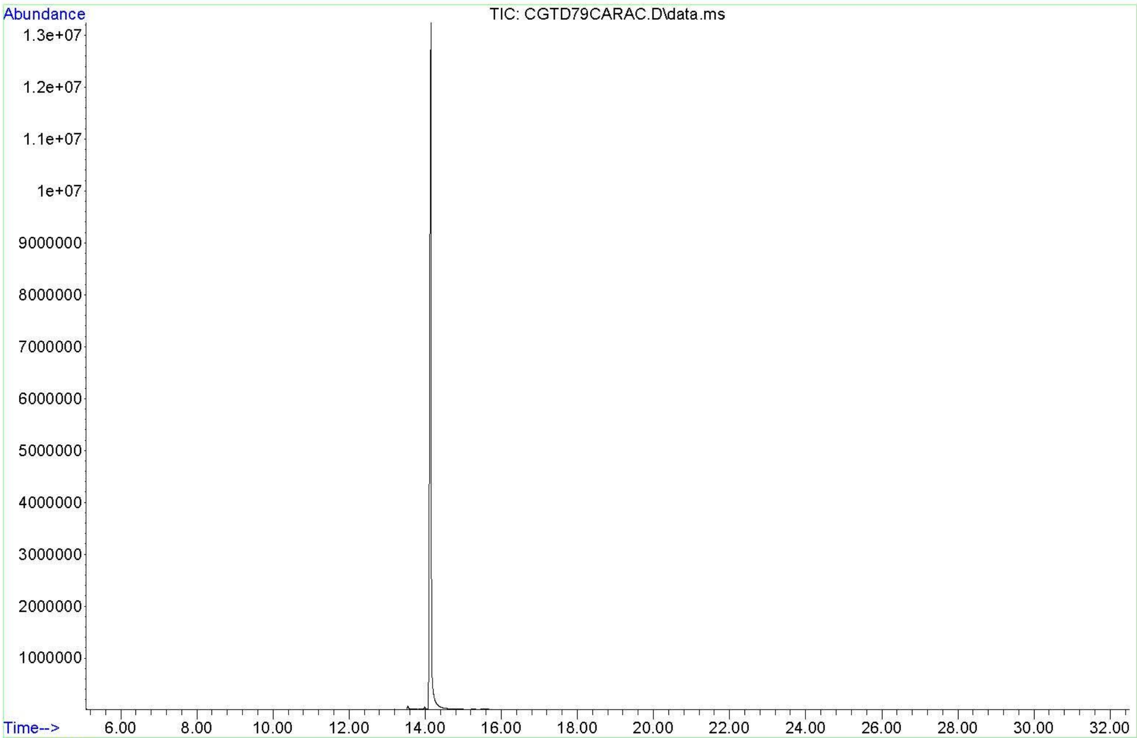

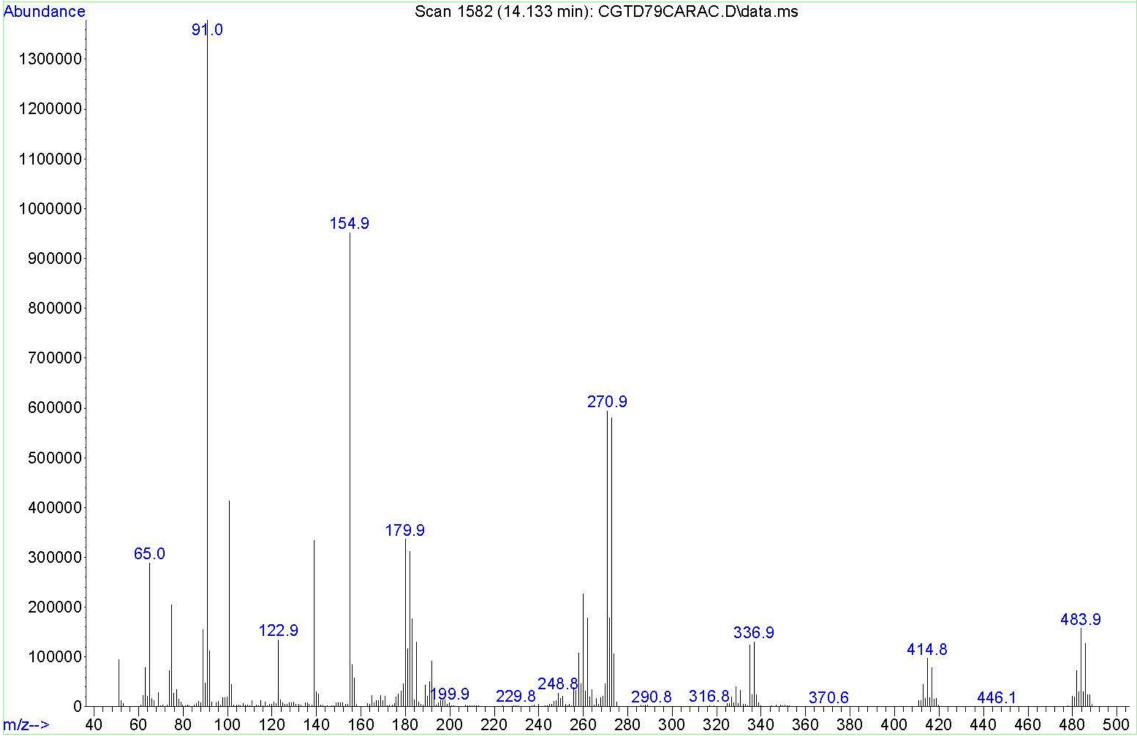

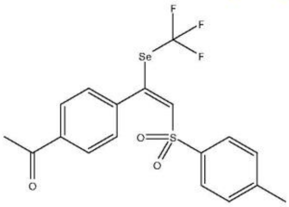

3g

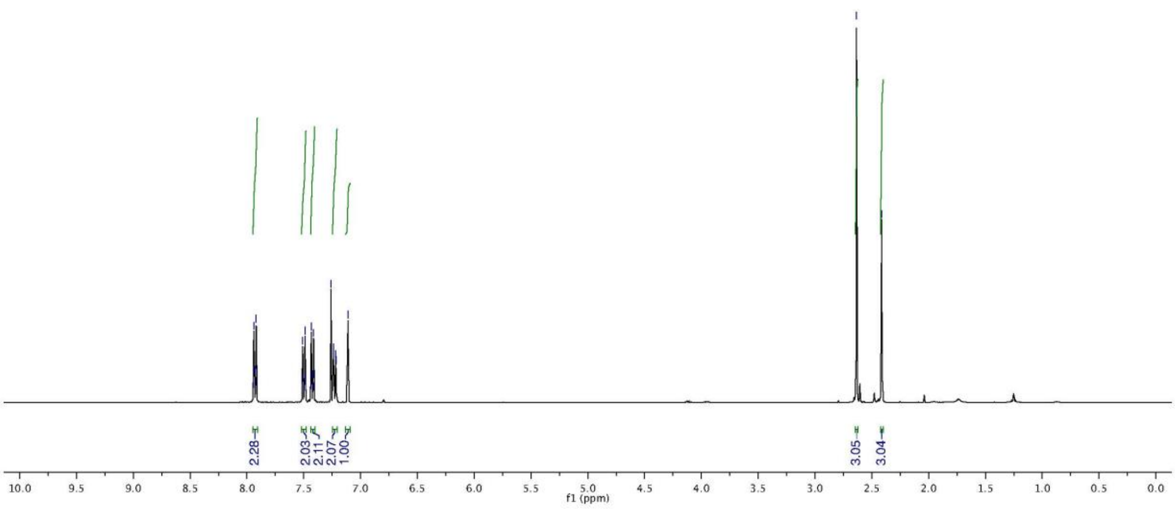
7.94
7.94
7.93
7.92
7.92
7.91
7.51
7.51
7.49
7.49
7.48
7.44
7.43
7.43
7.42
7.41
7.41
7.26
7.24
7.24
7.22
7.22
7.11
2.63
2.41
2.28
2.03
2.11
2.07
1.00
3.05
3.04
f1 (ppm)

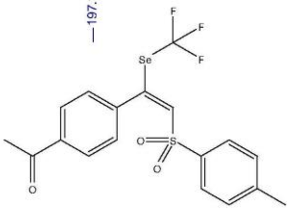

3g

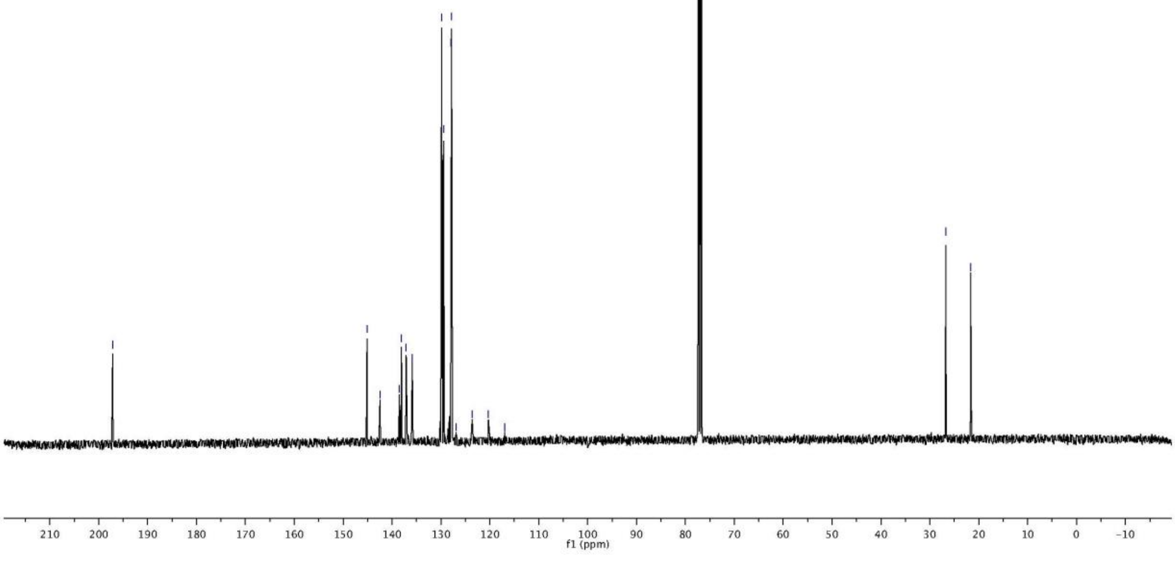
197.15
145.12
142.46
142.45
138.53
138.06
137.11
135.89
135.87
129.87
129.49
127.95
127.84
126.94
123.61
120.29
116.96
26.73
21.67
f1 (ppm)

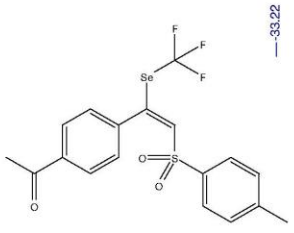

3g

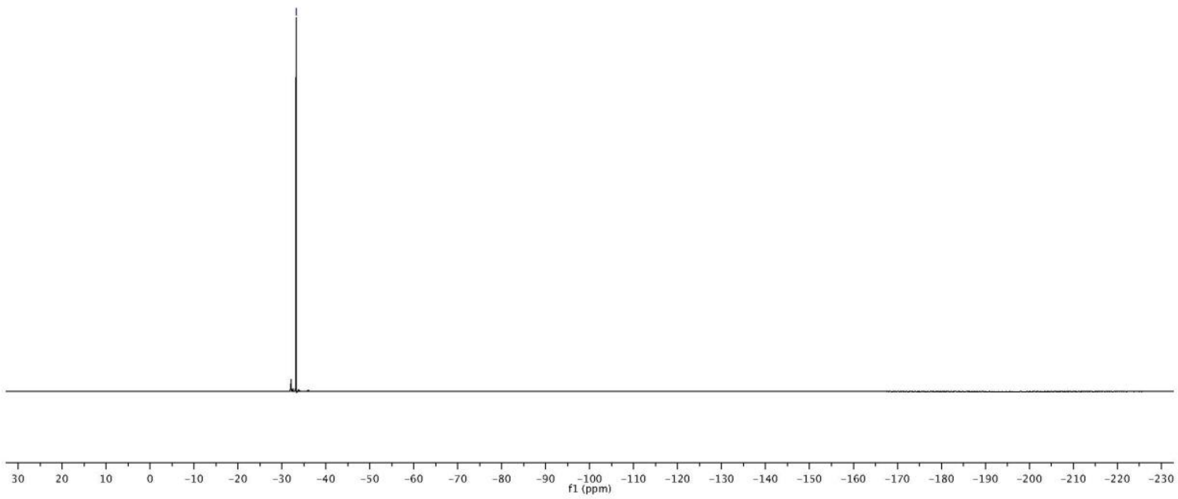

—33.22
30 20 10 0 −10 −20 −30 −40 −50 −60 −70 −80 −90 −100 −110 −120 −130 −140 −150 −160 −170 −180 −190 −200 −210 −220 −230
f1 (ppm)

```
File        :C:\msdchem\data\anis\CGVD02CARAC.D
Operator    :
Acquired    : 31 May 2017  15:36      using AcqMethod ANIS.M
Instrument :    GCMS
Sample Name: CGVD02CARAC
Misc Info   :
Vial Number: 2
```

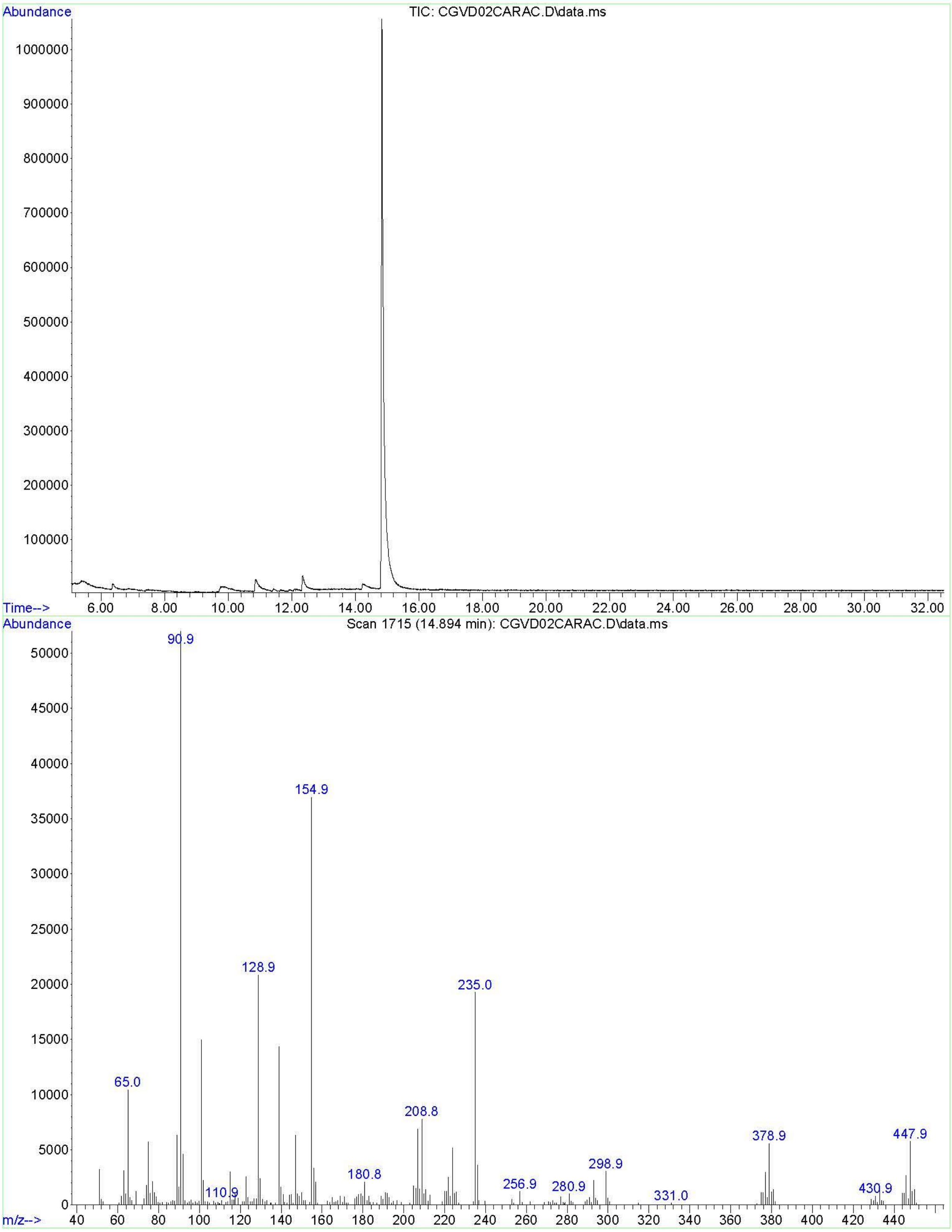

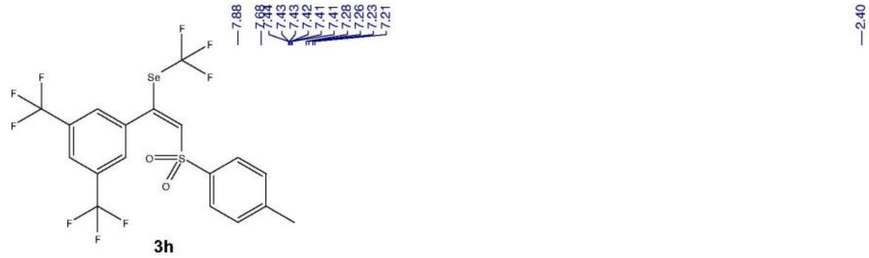

3h

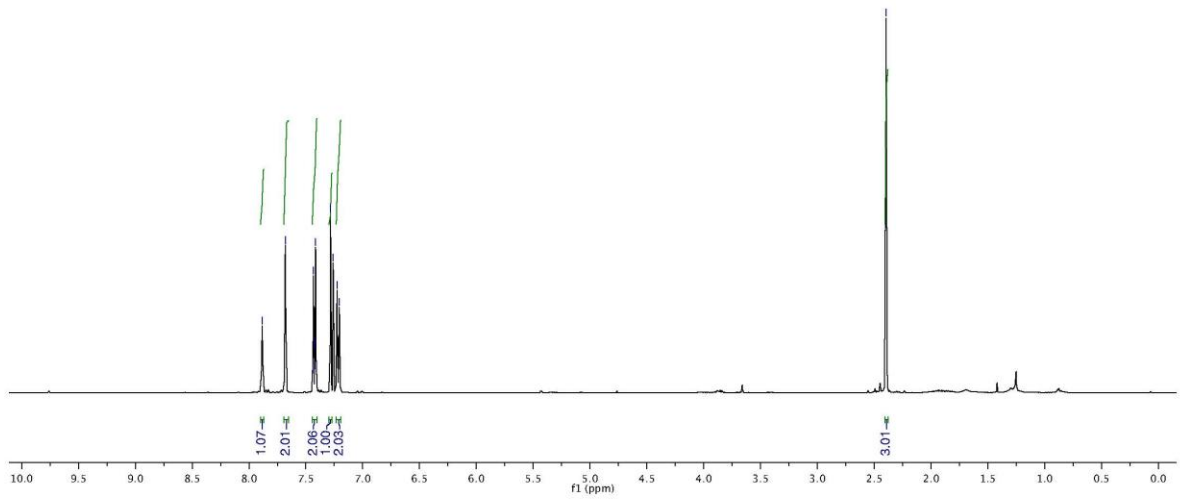

7.88
7.68
7.44
7.43
7.43
7.42
7.41
7.41
7.28
7.26
7.23
7.21
2.40
1.07
2.01
2.06
1.00
2.03
3.01
10.0 9.5 9.0 8.5 8.0 7.5 7.0 6.5 6.0 5.5 5.0 4.5 4.0 3.5 3.0 2.5 2.0 1.5 1.0 0.5 0.0
f1 (ppm)

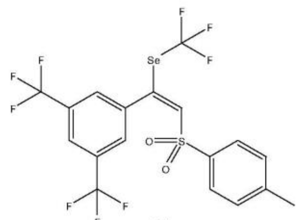

3h

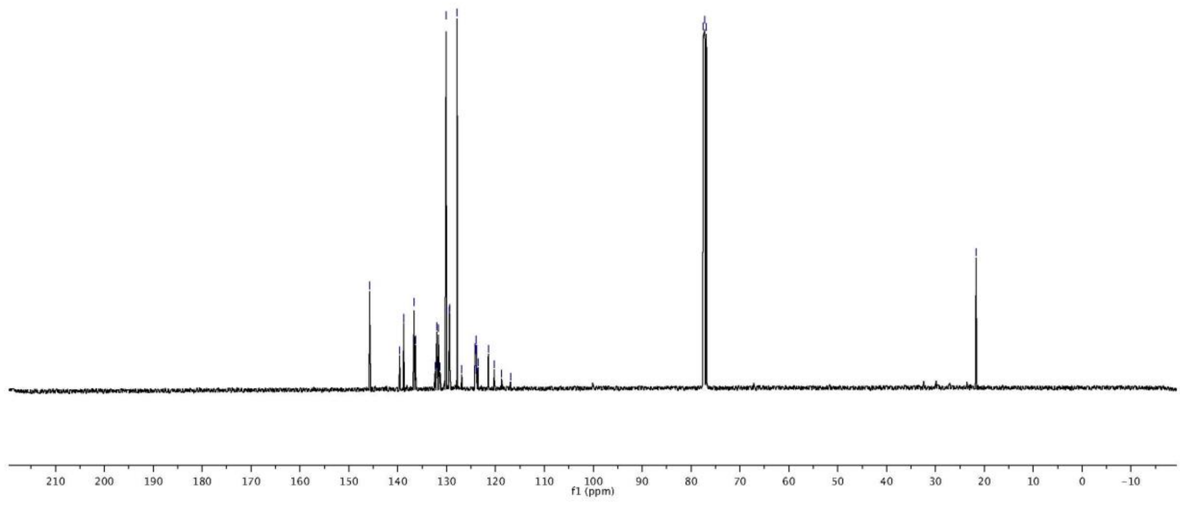

145.79
139.63
138.78
138.77
136.64
136.38
132.34
132.00
131.66
131.33
130.11
129.43
129.40
127.85
126.90
124.18
124.00
123.96
123.93
123.89
123.85
123.58
121.47
120.25
77.48
77.16
76.84
21.69
210 200 190 180 170 160 150 140 130 120 110 100 90 80 70 60 50 40 30 20 10 0 −10
f1 (ppm)

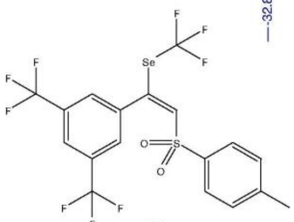
F
F
F
Se
F
F
F
O
S
O
F
F
F

3h

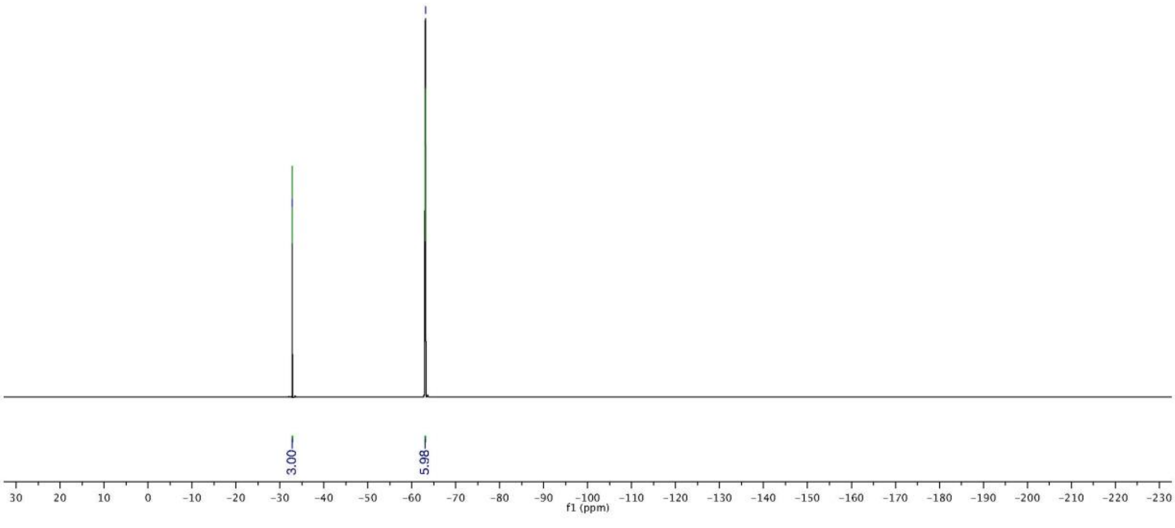
-32.84
-63.09
3.00
5.98
30
20
10
0
-10
-20
-30
-40
-50
-60
-70
-80
-90
-100
-110
-120
-130
-140
-150
-160
-170
-180
-190
-200
-210
-220
-230
f1 (ppm)

File :C:\msdchem\data\anis\CGTD115CARAC.D
Operator :
Acquired : 24 Jul 2017 10:40 using AcqMethod anis.M
Instrument : GCMS
Sample Name: cgtd115carac
Misc Info :
Vial Number: 2

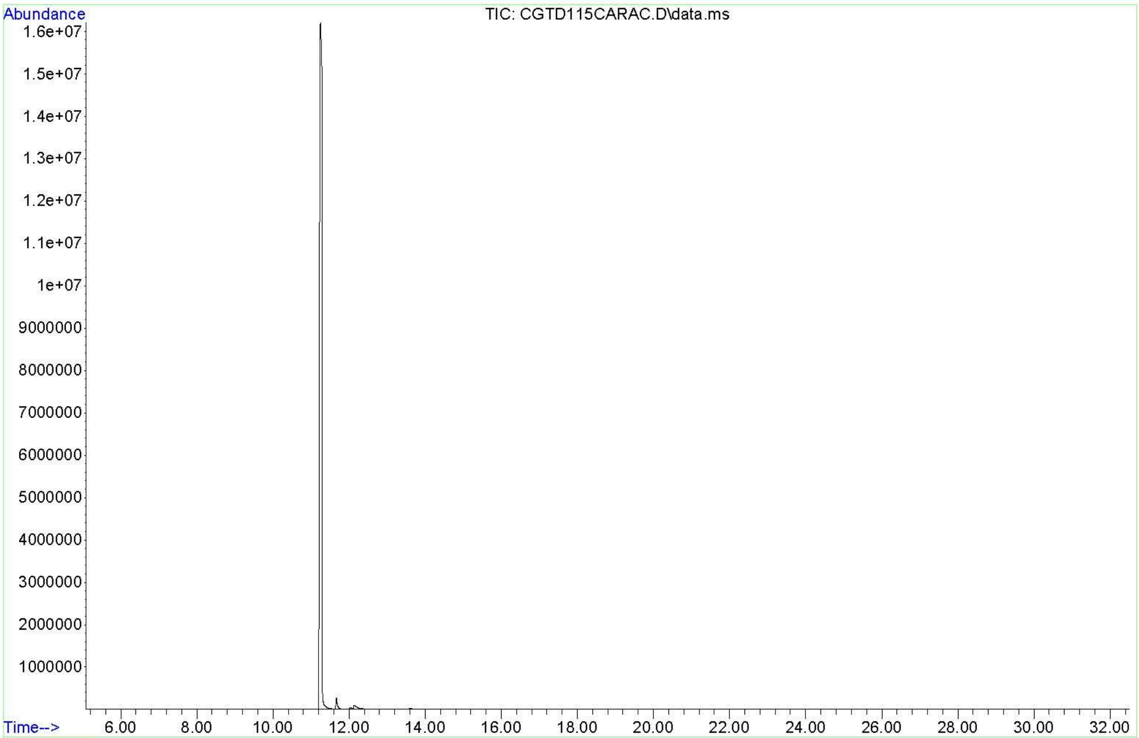

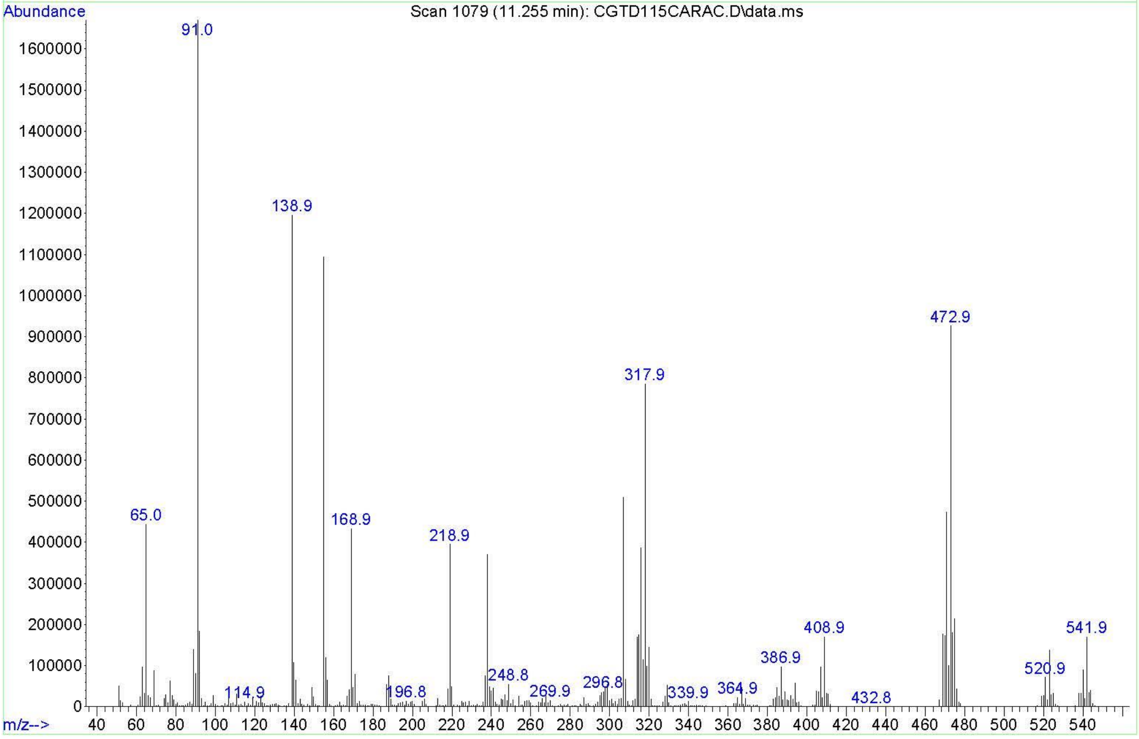

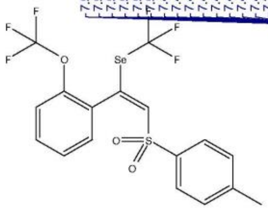

3i

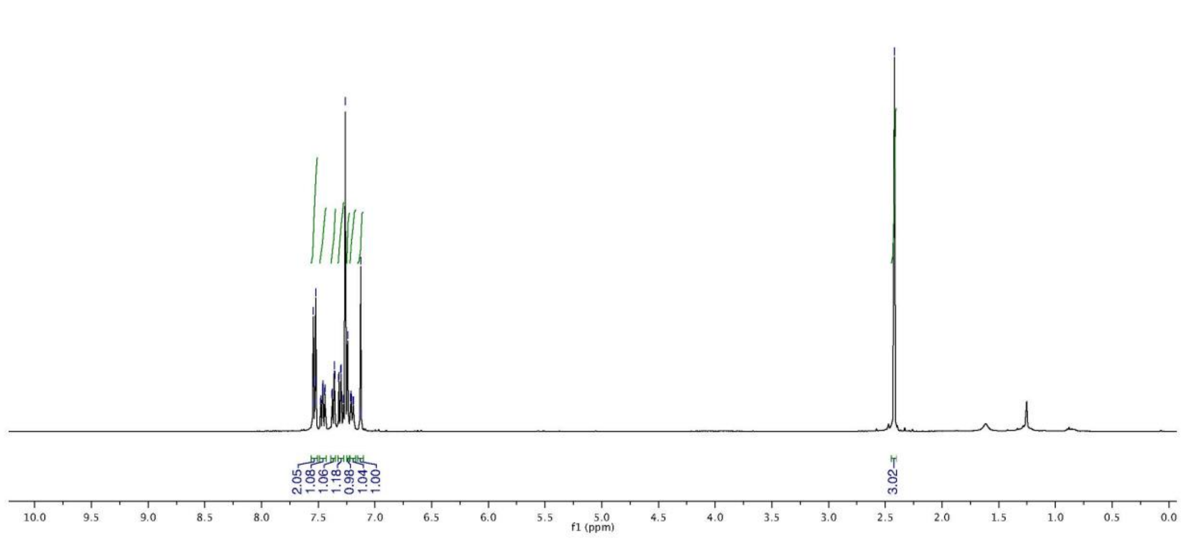

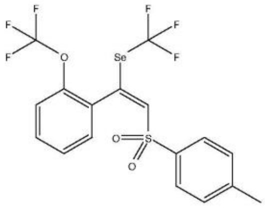

3i

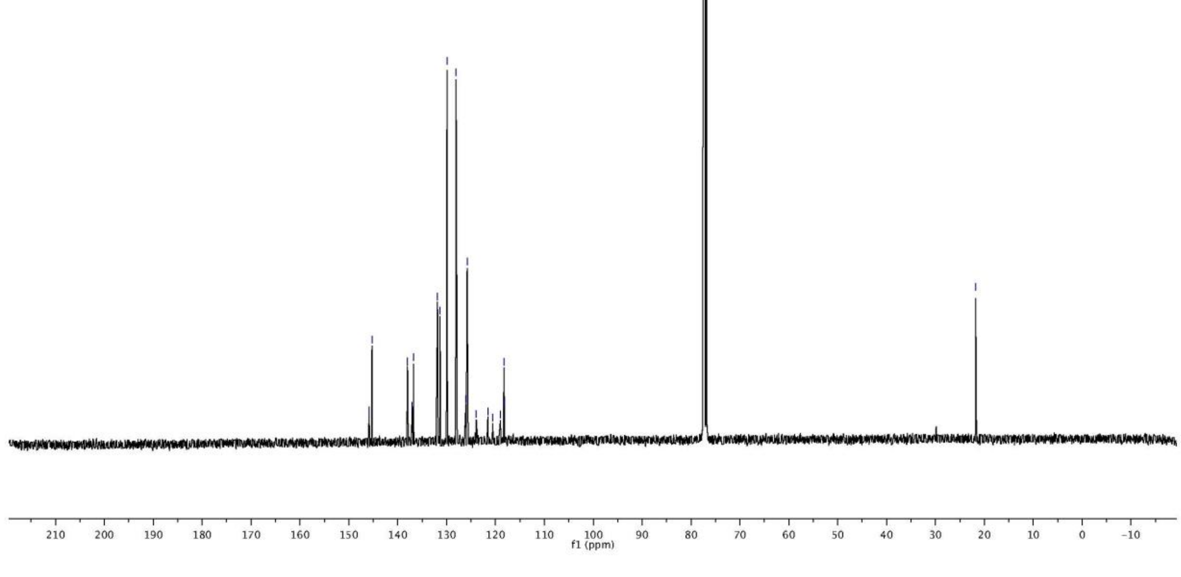

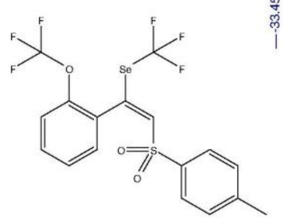

3i

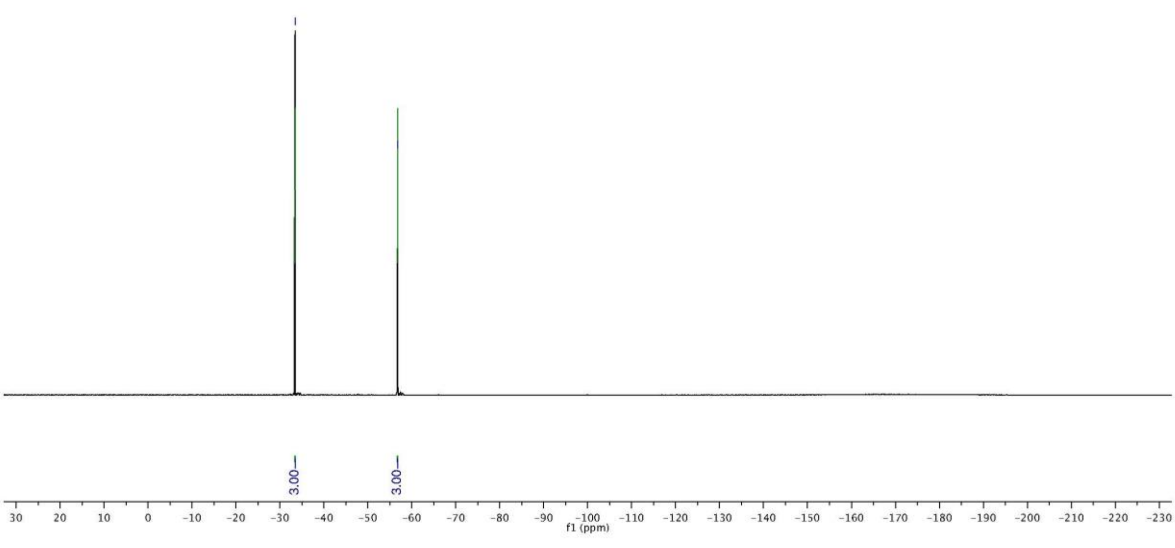
-33.45
-56.75
3.00
3.00
30 20 10 0 -10 -20 -30 -40 -50 -60 -70 -80 -90 -100 -110 -120 -130 -140 -150 -160 -170 -180 -190 -200 -210 -220 -230
f1 (ppm)

```
File        :C:\msdchem\data\anis\CG317CARAC.D
Operator    :
Acquired    : 24 Jul 2017  11:34      using AcqMethod anis.M
Instrument  :    GCMS
Sample Name: cg317carac
Misc Info   :
Vial Number: 2
```

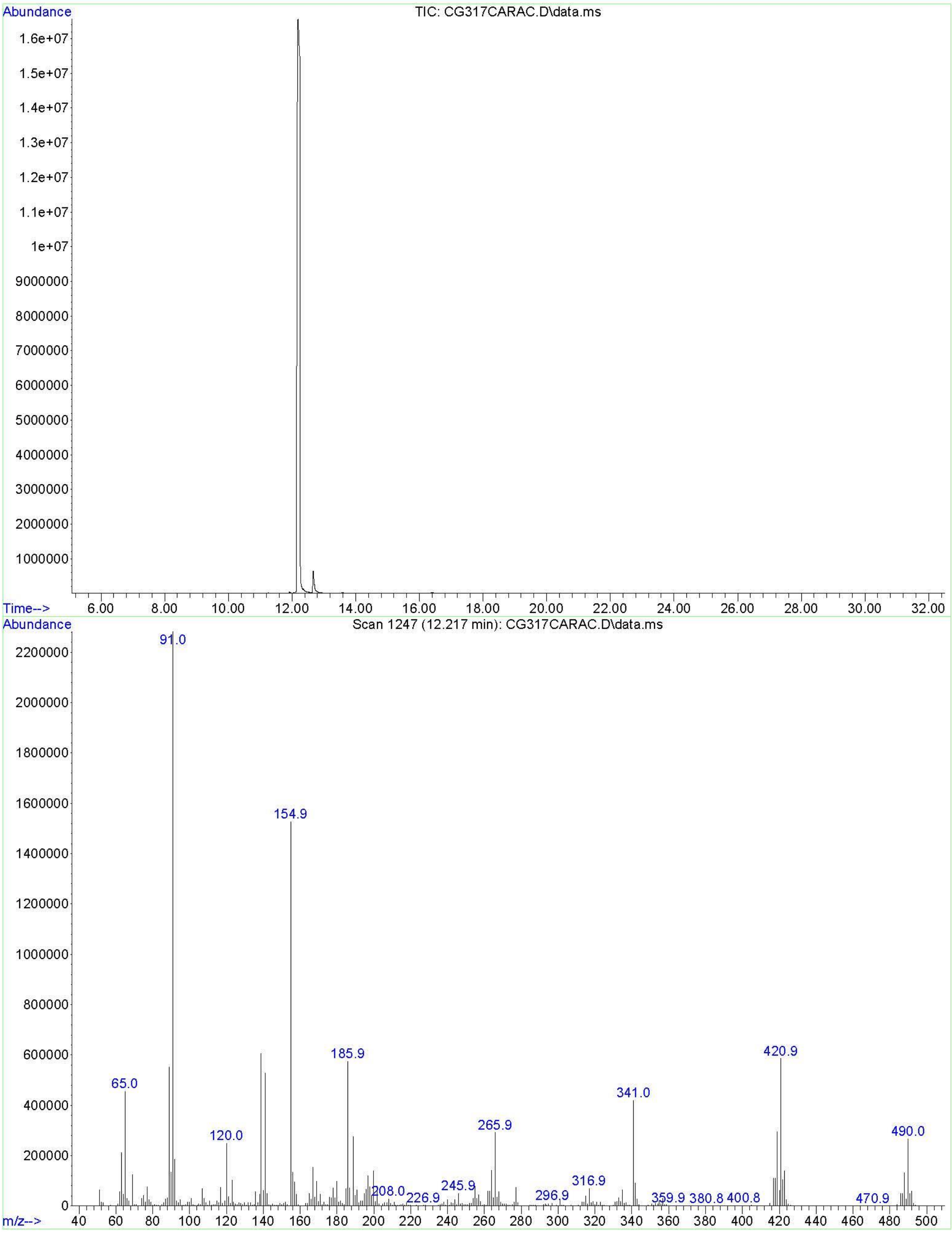

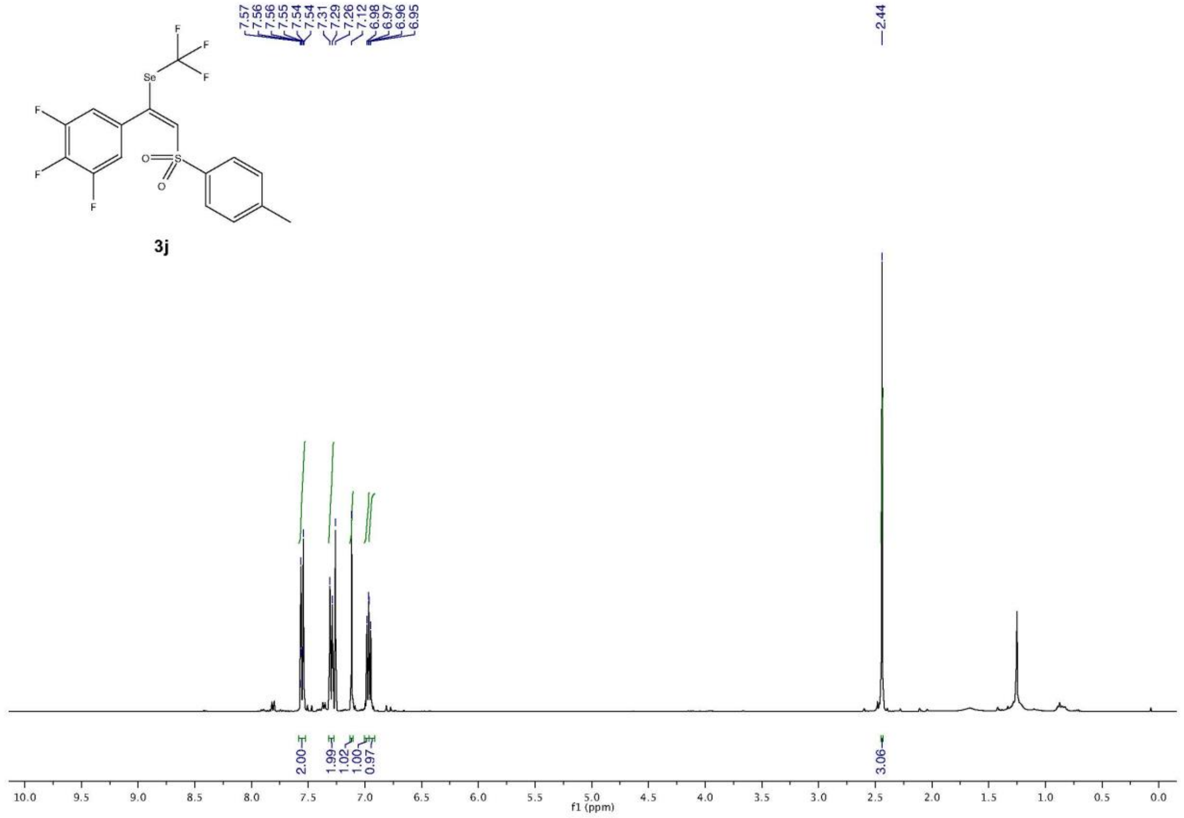
Se
F
F
F
O
S
O
3j
7.57
7.56
7.55
7.54
7.54
7.31
7.29
7.26
7.12
6.98
6.97
6.96
6.95
2.44
2.00
1.99
1.02
1.00
0.97
3.06
f1 (ppm)

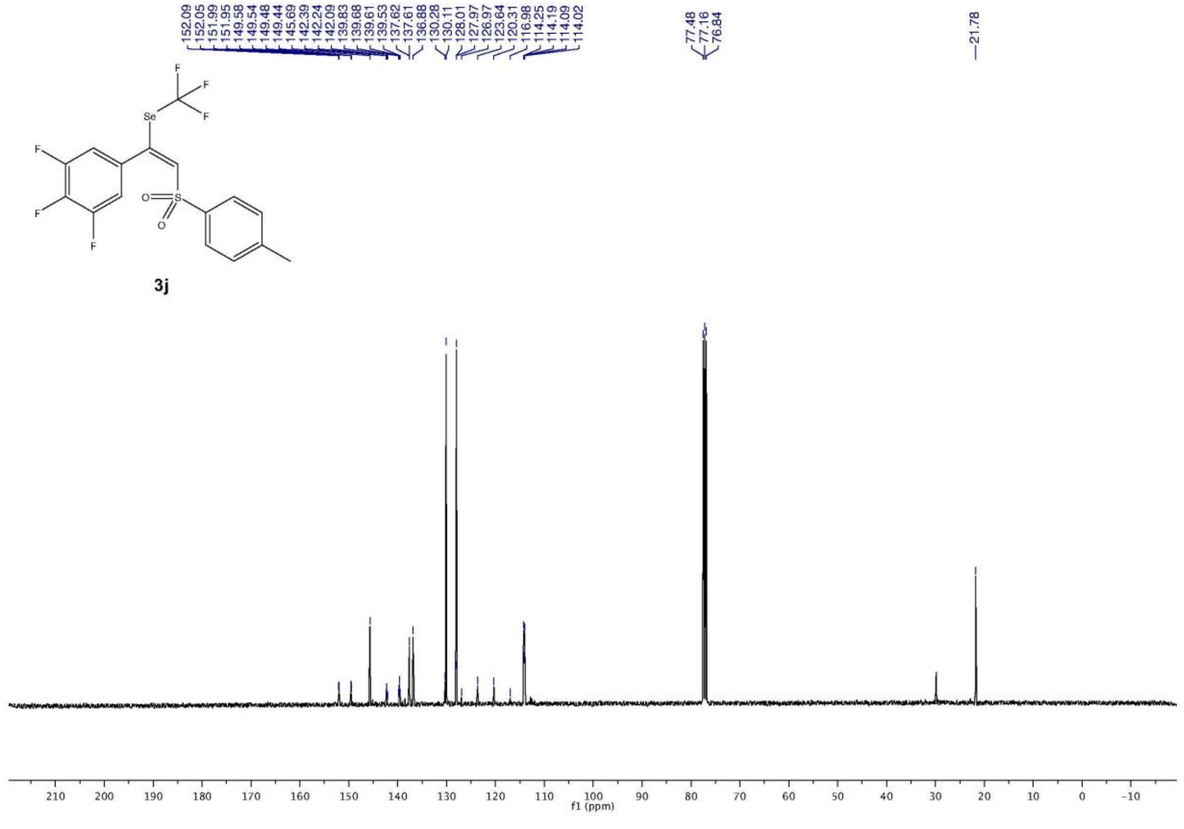
Se
F
F
F
O
S
O
3j
152.09
152.05
151.99
151.95
149.58
149.54
149.48
149.44
145.69
142.39
142.24
142.09
139.83
139.68
139.61
139.53
137.62
137.61
136.88
130.28
130.11
128.01
127.97
126.97
123.64
120.31
116.98
114.25
114.19
114.09
114.02
77.48
77.16
76.84
21.78
f1 (ppm)

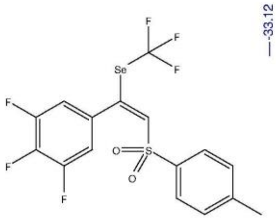

3j

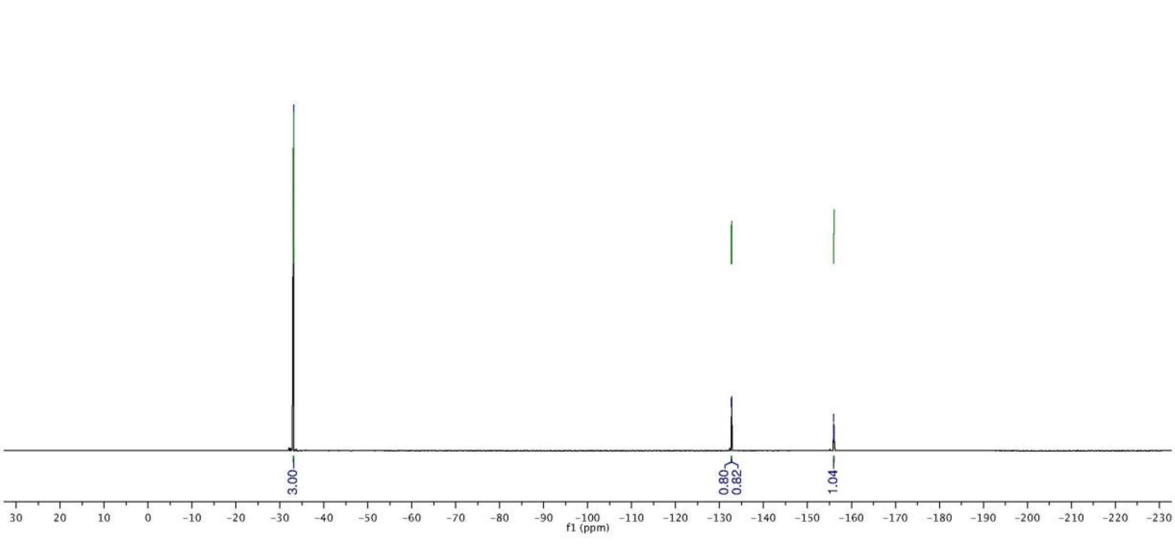
-33.12
-132.68
-132.70
-132.74
-132.76
-155.92
-155.94
-155.95
-155.97
-155.99
-156.01
-156.03
-156.04
-156.06
3.00
0.80
0.82
1.04
30 20 10 0 -10 -20 -30 -40 -50 -60 -70 -80 -90 -100 -110 -120 -130 -140 -150 -160 -170 -180 -190 -200 -210 -220 -230
f1 (ppm)

```
File        :C:\msdchem\data\anis\CG318CARAC.D
Operator    :
Acquired    : 21 Jul 2017  17:44     using AcqMethod anis.M
Instrument  :    GCMS
Sample Name: cg318carac
Misc Info   :
Vial Number: 2
```

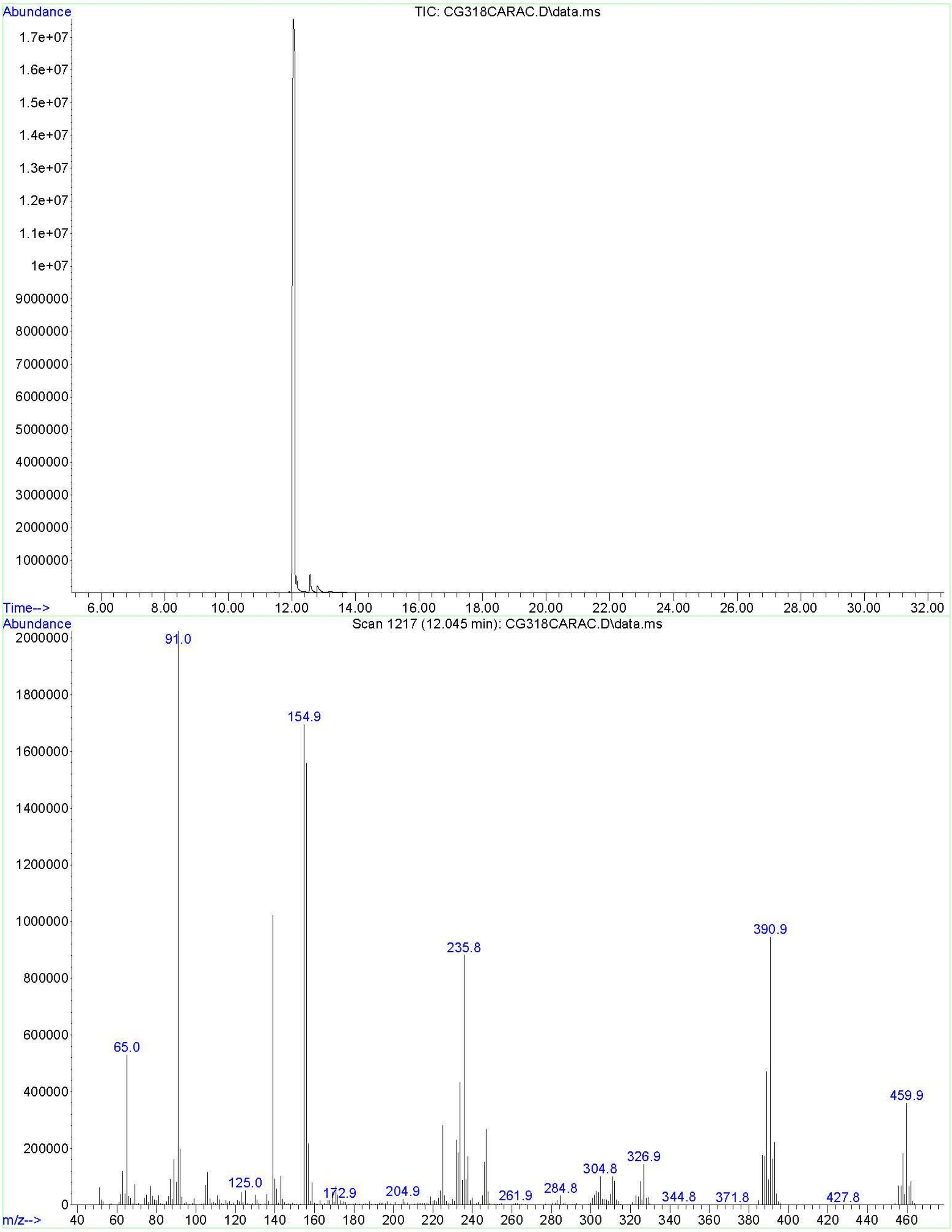

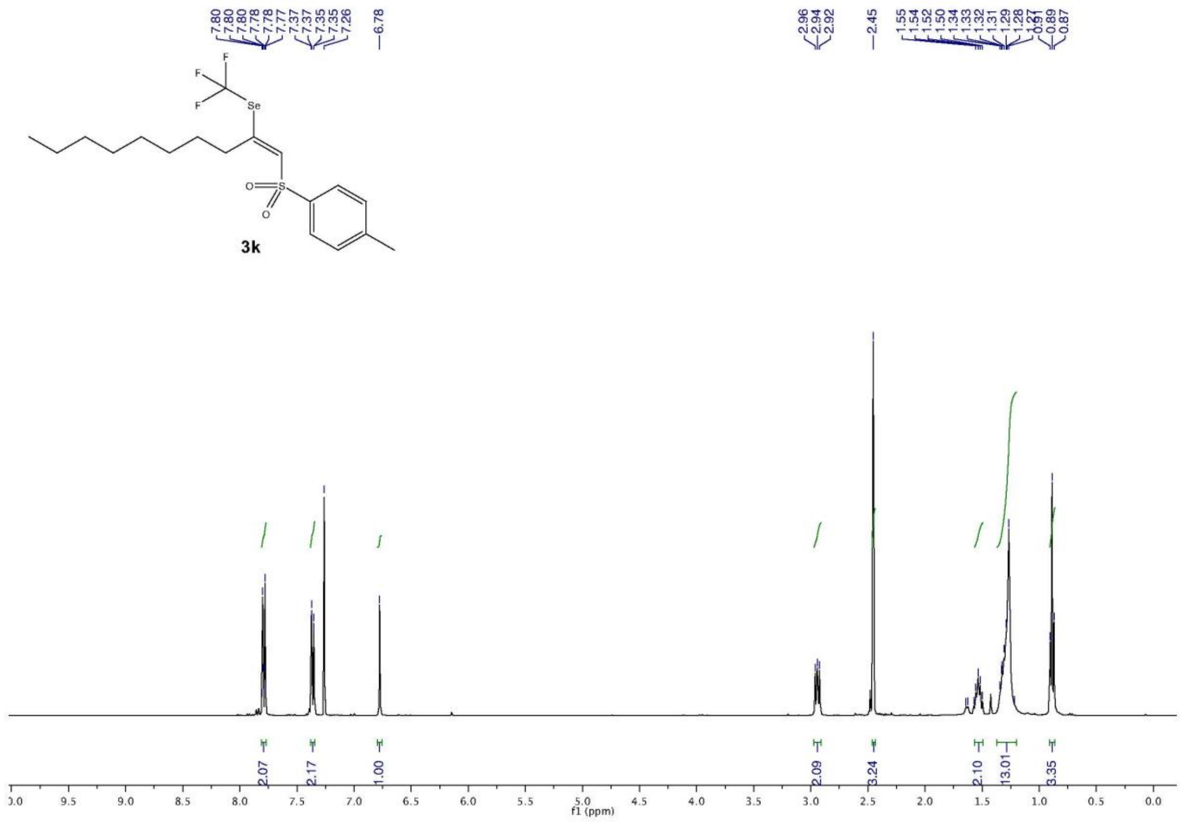
7.80
7.80
7.80
7.78
7.77
7.37
7.37
7.35
7.35
7.26
6.78
2.96
2.94
2.92
2.45
1.55
1.54
1.52
1.50
1.34
1.33
1.32
1.31
1.29
1.28
1.27
0.91
0.89
0.87
F
F
F
Se
O
S
O
3k
2.07
2.17
1.00
2.09
3.24
2.10
13.01
3.35
f1 (ppm)

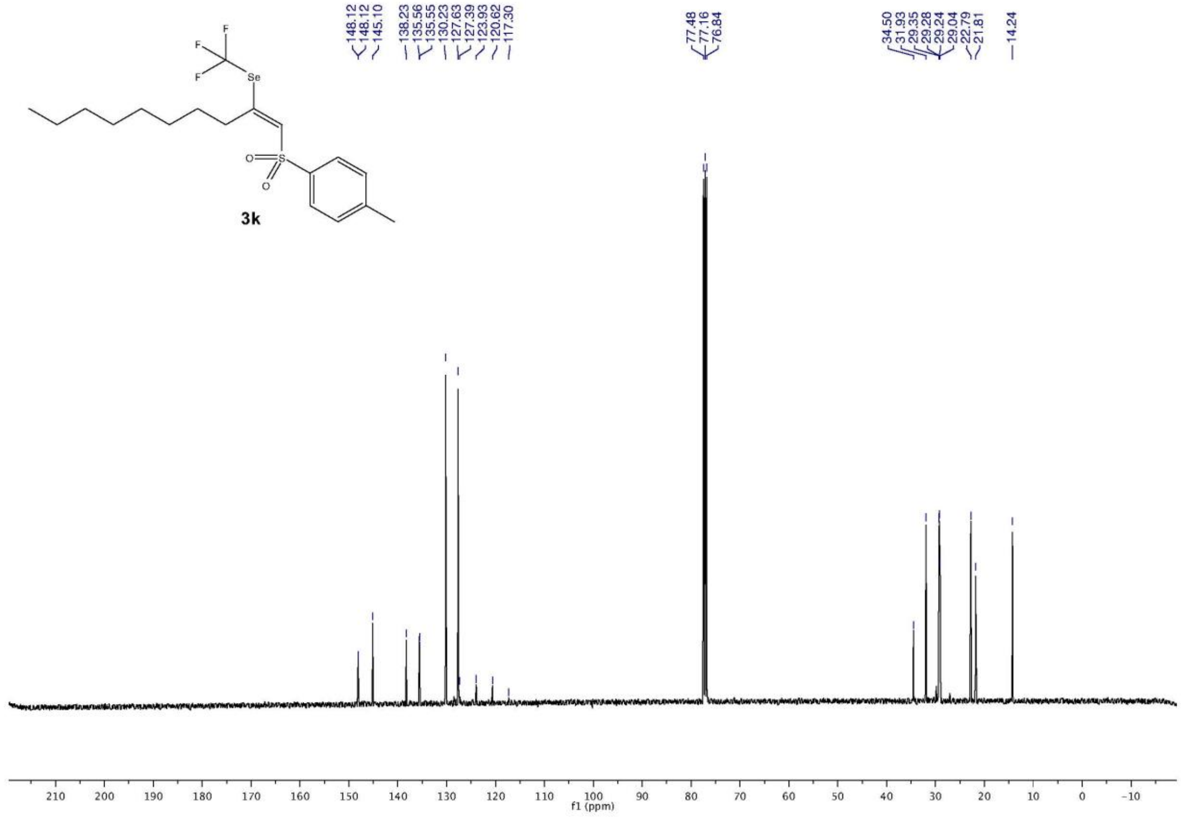
148.12
148.12
145.10
138.23
135.56
135.55
130.23
127.63
127.39
123.93
120.62
117.30
77.48
77.16
76.84
34.50
31.93
29.35
29.28
29.24
29.04
22.79
21.81
14.24
F
F
F
Se
O
S
O
3k
f1 (ppm)

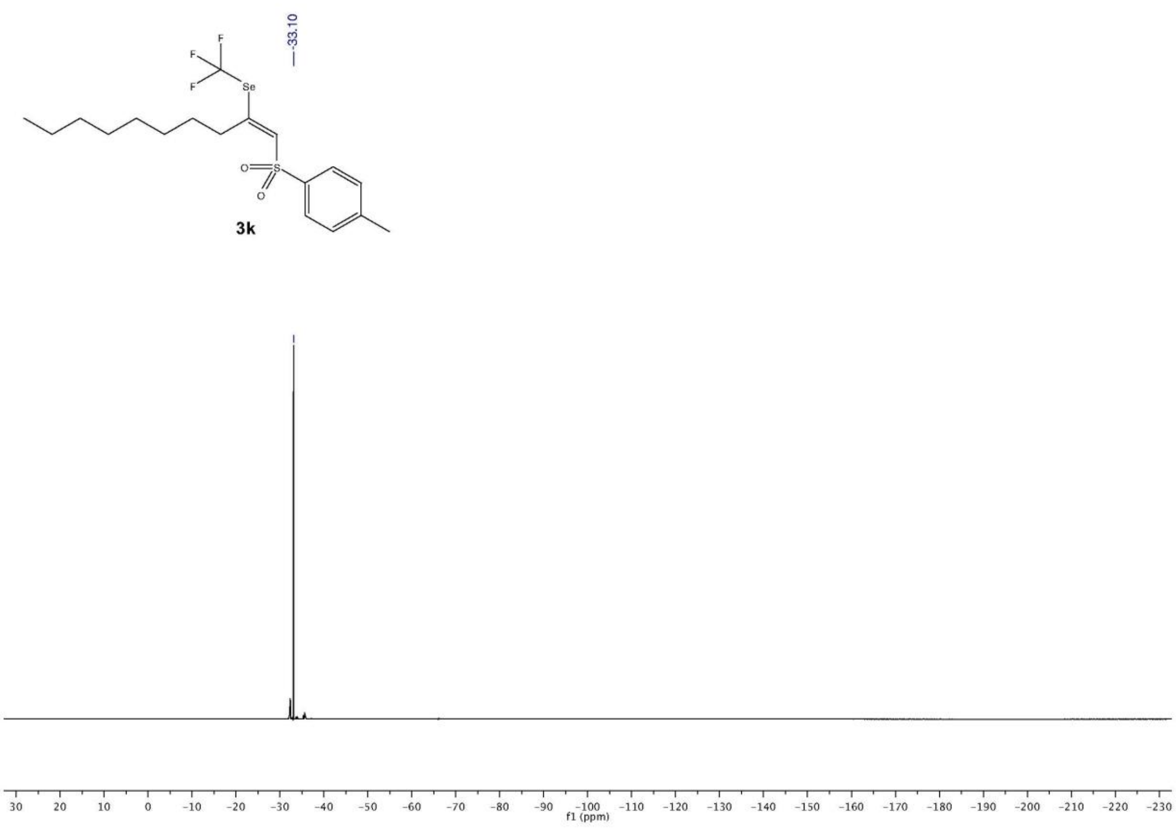

F
F
F
Se
O
S
O
3k
-33.10
30 20 10 0 -10 -20 -30 -40 -50 -60 -70 -80 -90 -100 -110 -120 -130 -140 -150 -160 -170 -180 -190 -200 -210 -220 -230
f1 (ppm)

```
File        :C:\msdchem\data\anis\CG291CARAC2.D
Operator    :
Acquired    : 28 Jul 2017  13:31      using AcqMethod anis.M
Instrument :     GCMS
Sample Name: cg291carac2
Misc Info   :
Vial Number: 2
```

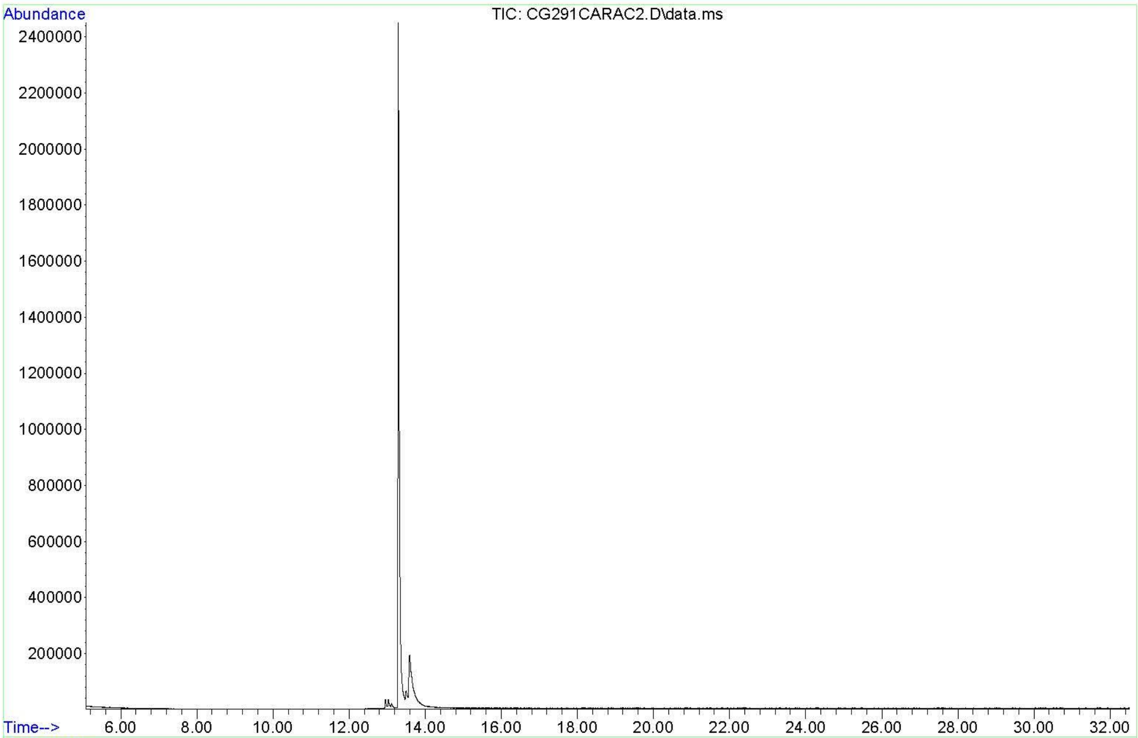

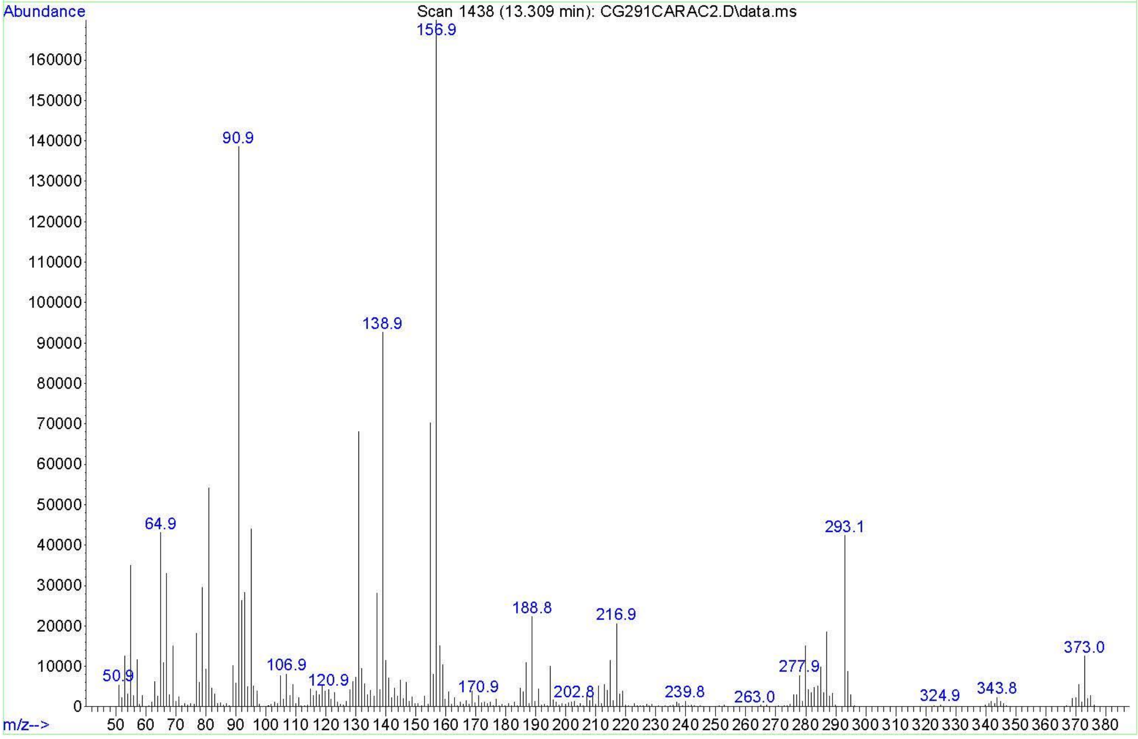

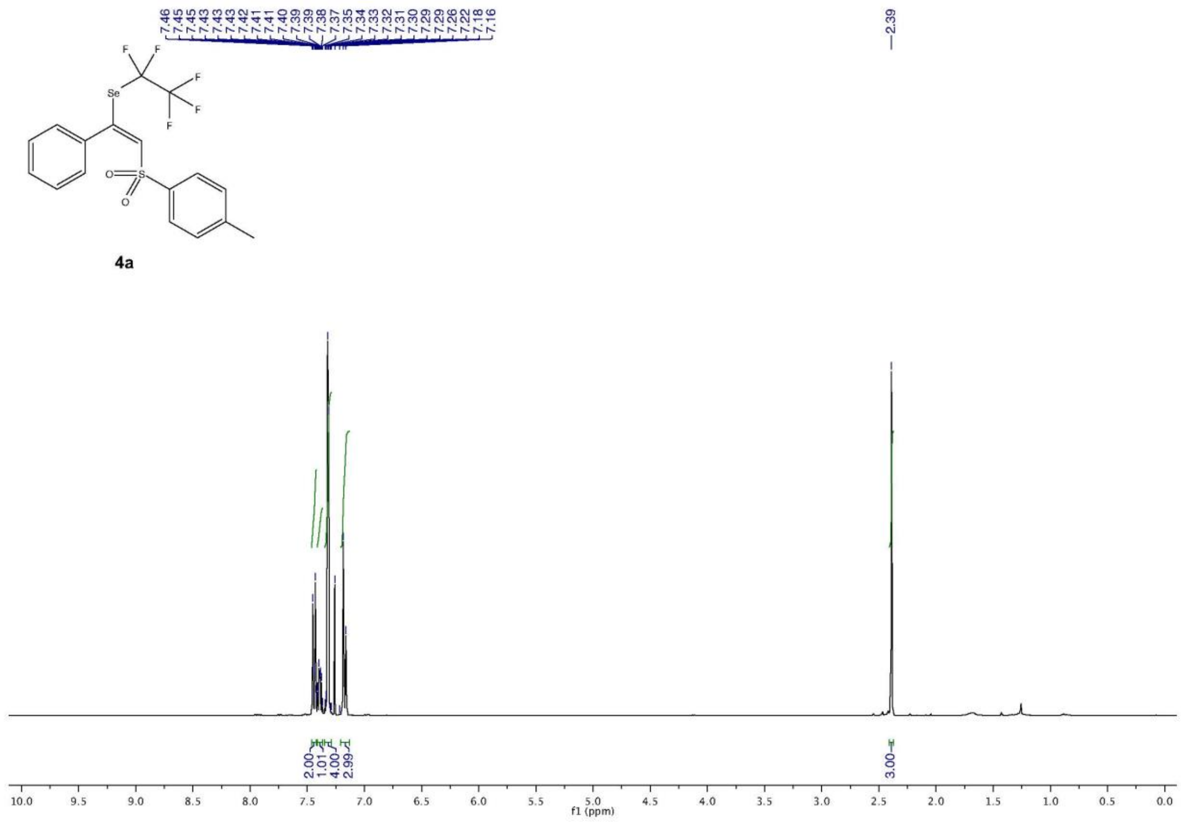

Se
F
4a
7.46
7.45
7.43
7.43
7.43
7.42
7.41
7.40
7.39
7.39
7.38
7.37
7.35
7.34
7.33
7.32
7.31
7.30
7.29
7.29
7.26
7.22
7.18
7.16
2.39
2.00
1.01
4.00
2.99
3.00
f1 (ppm)

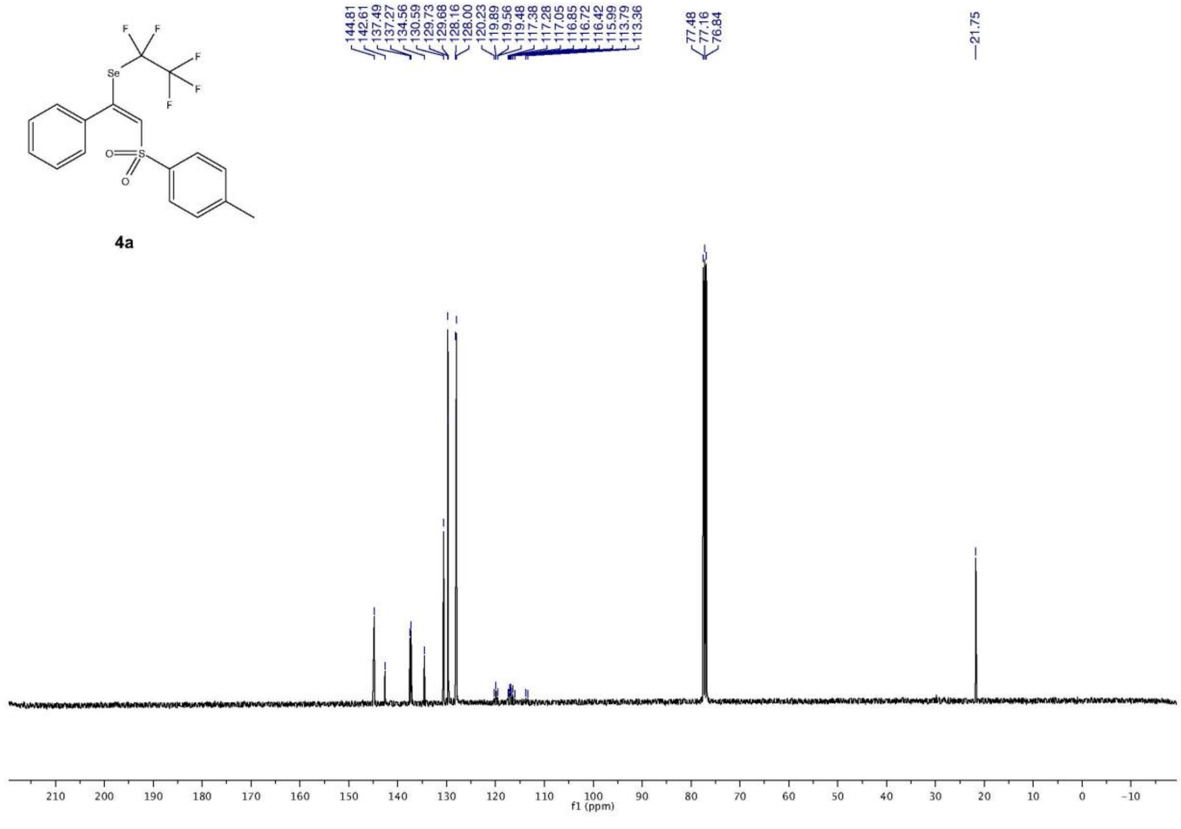

Se
F
4a
144.81
142.61
137.49
137.27
134.56
130.59
129.73
129.68
128.16
128.00
120.23
119.89
119.56
119.48
117.38
117.28
117.05
116.85
116.72
116.42
115.99
113.79
113.36
77.48
77.16
76.84
21.75
f1 (ppm)

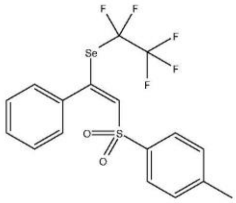

4a

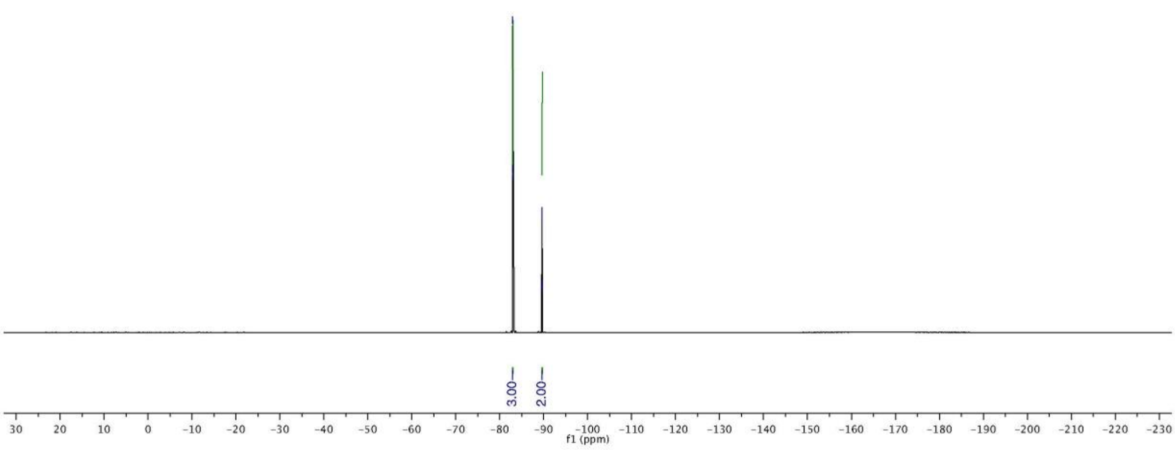
-82.94
-82.95
-82.96
-89.64
-89.65
-89.66
-89.67
3.00
2.00
30 20 10 0 -10 -20 -30 -40 -50 -60 -70 -80 -90 -100 -110 -120 -130 -140 -150 -160 -170 -180 -190 -200 -210 -220 -230
f1 (ppm)

```
File        :C:\msdchem\data\anis\CG325CARAC.D
Operator    :
Acquired    : 11 Jul 2017  12:35      using AcqMethod ANIS.M
Instrument  :     GCMS
Sample Name: CG325CARAC
Misc Info   :
Vial Number: 2
```

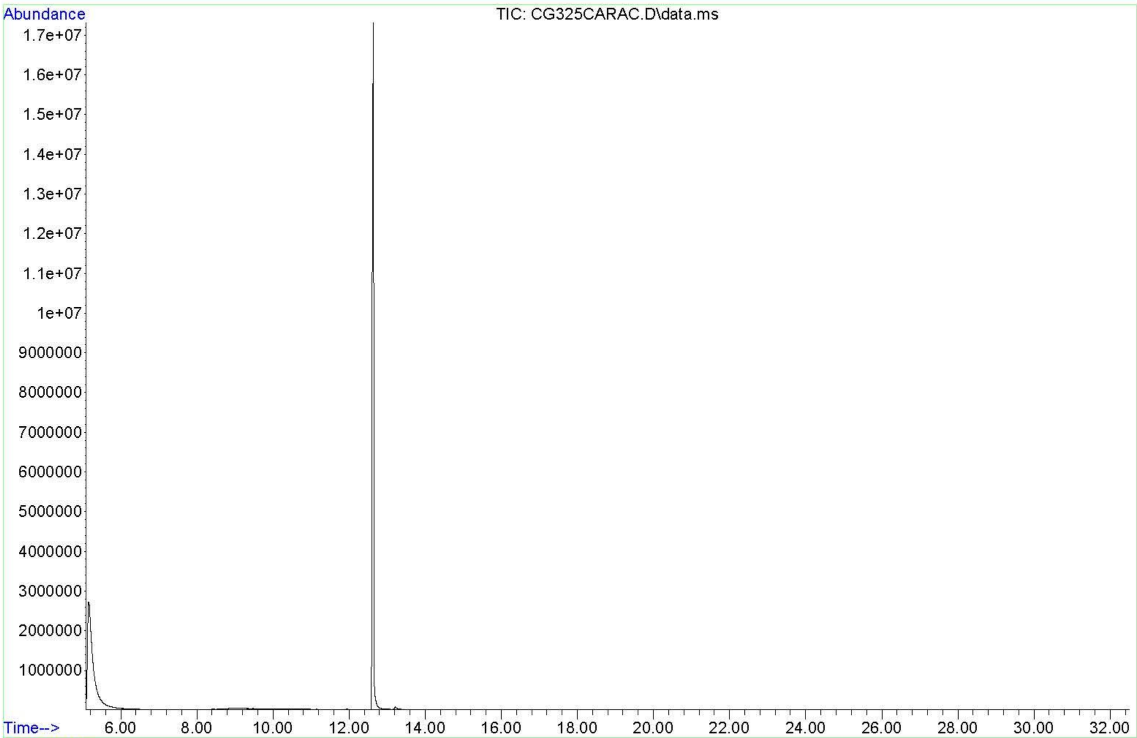

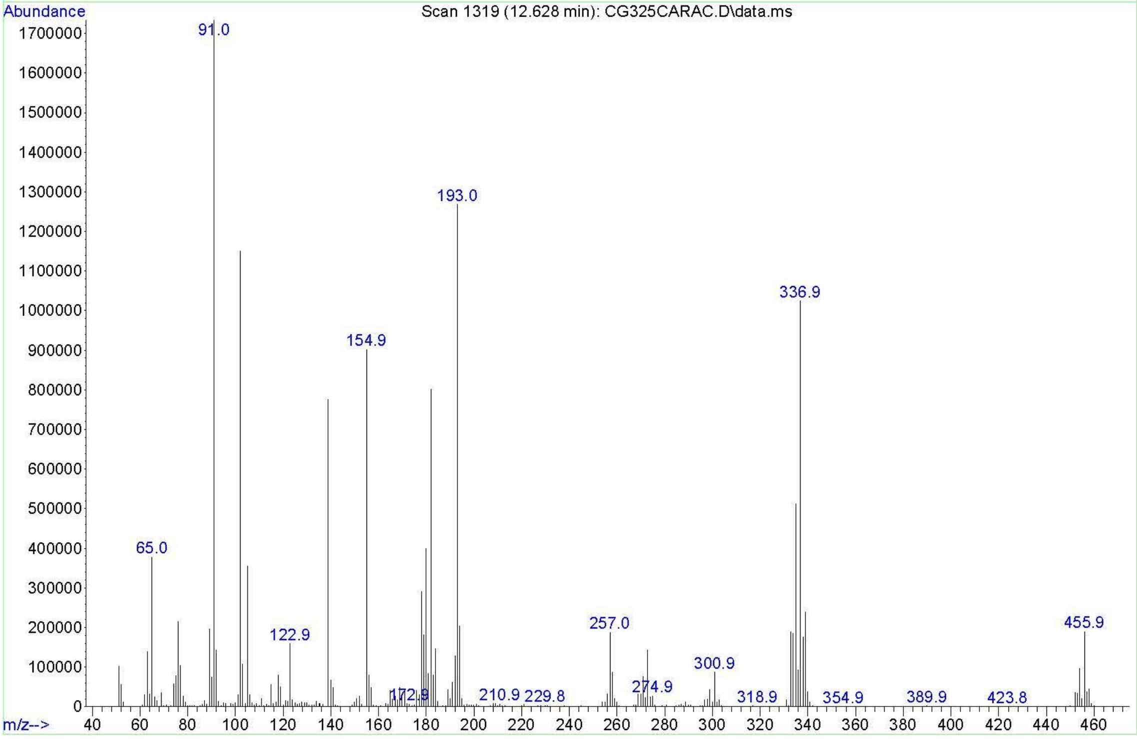

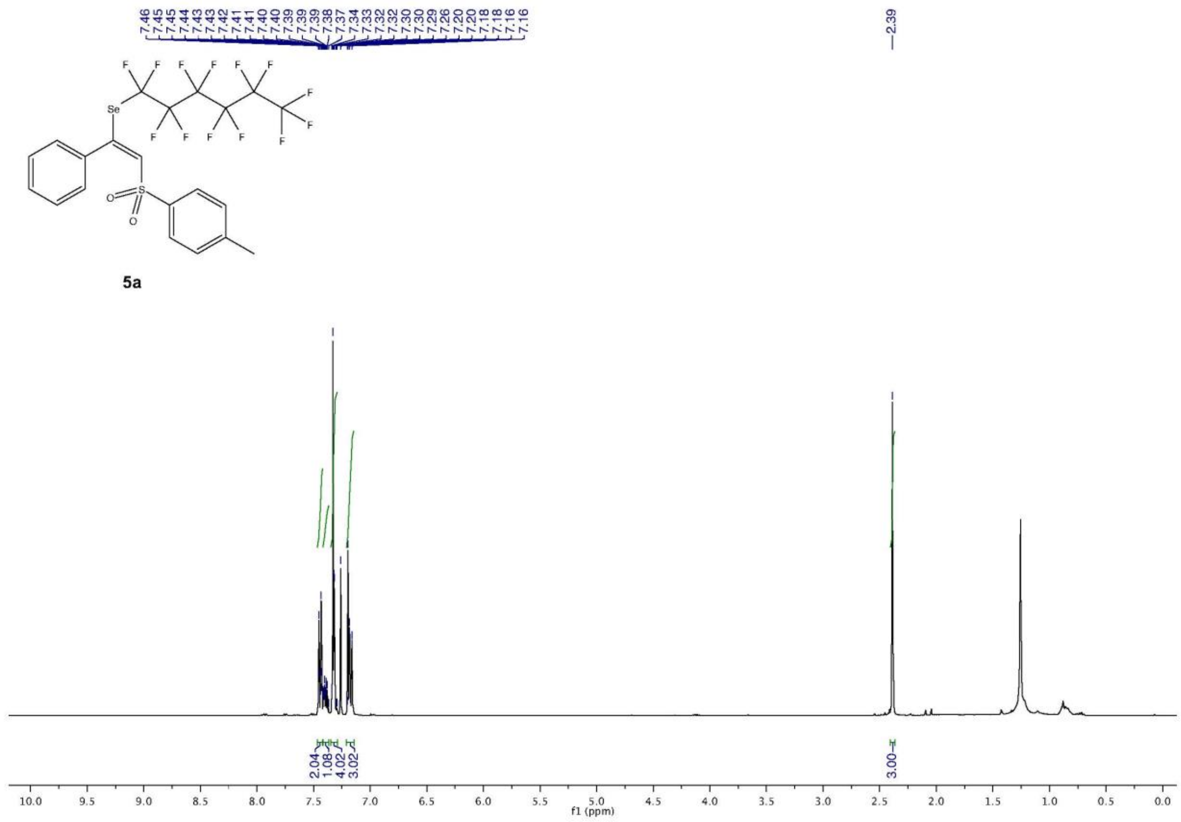
7.46
7.45
7.44
7.43
7.43
7.42
7.41
7.40
7.39
7.39
7.39
7.38
7.37
7.34
7.33
7.32
7.32
7.30
7.30
7.29
7.26
7.20
7.20
7.18
7.16
7.16
2.39
Se
5a
2.04
1.08
4.02
3.02
3.00
f1 (ppm)

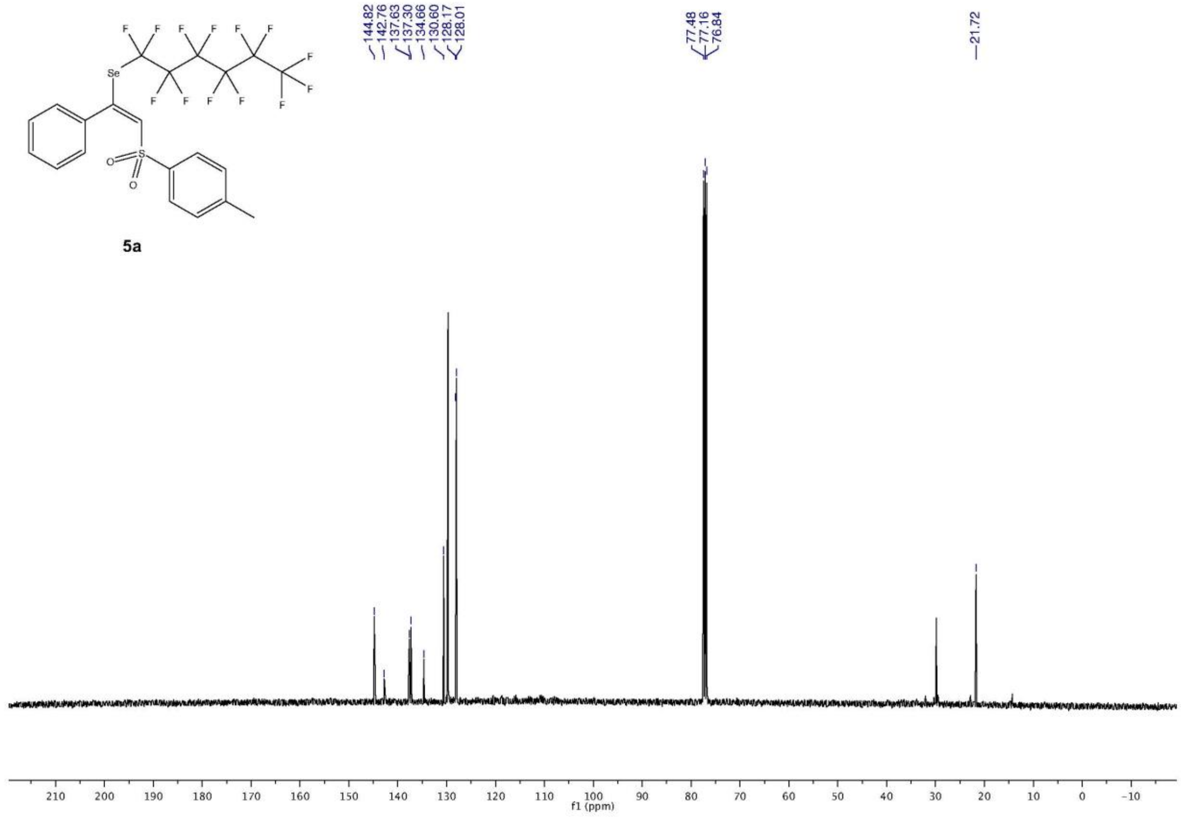
144.82
142.76
137.63
137.30
134.66
130.60
128.17
128.01
77.48
77.16
76.84
21.72
Se
5a
f1 (ppm)

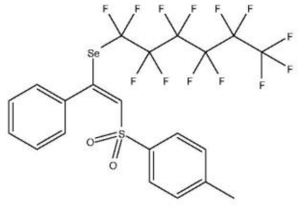

5a

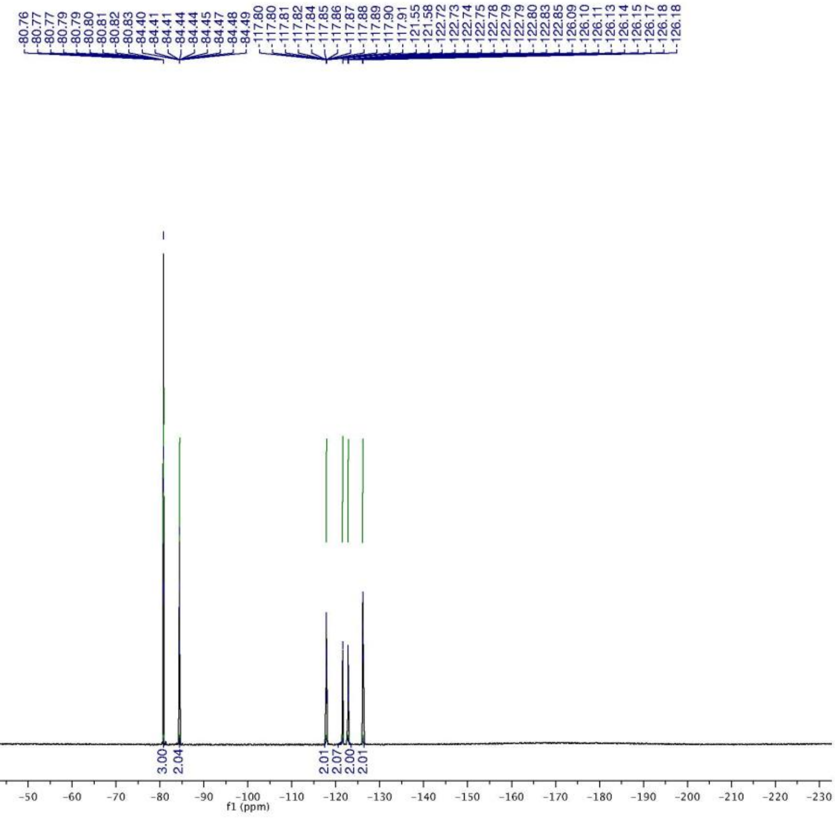

-80.76
-80.77
-80.77
-80.79
-80.79
-80.80
-80.81
-80.82
-80.83
-84.40
-84.41
-84.41
-84.44
-84.44
-84.45
-84.47
-84.48
-84.49
-117.80
-117.80
-117.81
-117.82
-117.84
-117.85
-117.86
-117.87
-117.88
-117.89
-117.90
-117.91
-121.55
-121.58
-122.72
-122.73
-122.74
-122.75
-122.78
-122.79
-122.79
-122.80
-122.83
-122.85
-126.09
-126.10
-126.11
-126.13
-126.14
-126.15
-126.17
-126.18
-126.18
3.00
2.04
2.01
2.07
2.00
2.01
f1 (ppm)

```
File        :C:\MSDCHEM\DATA\ANIS\Snapshot\CG441CARAC.D
Operator    :
Acquired    : 23 Aug 2017  13:35      using AcqMethod anis.M
Instrument  :    GCMS
Sample Name: cg441carac
Misc Info   :
Vial Number: 2
```

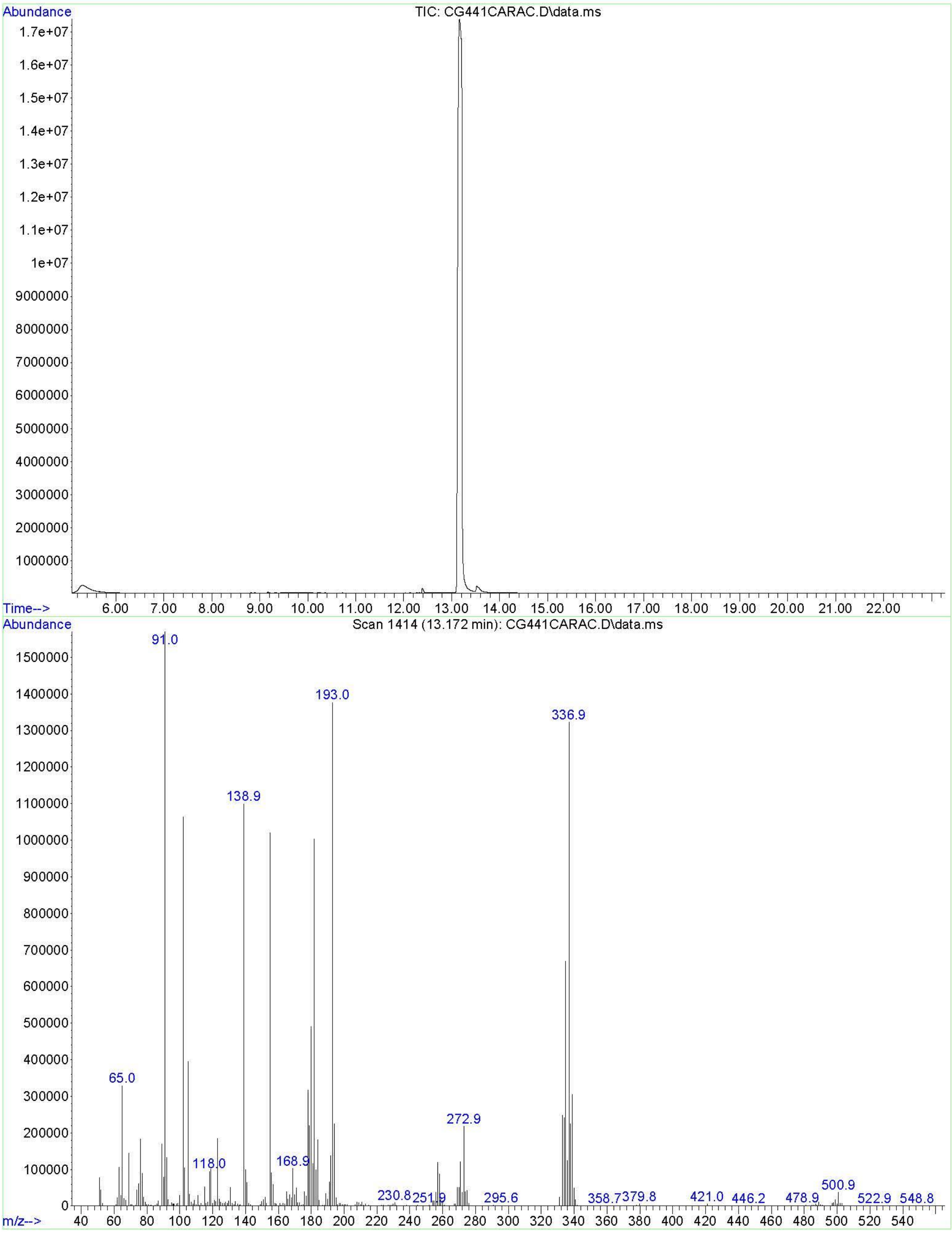

Supplement: File 1 — Additional experimental and analytical data and NMR spectra. [file Beilstein_J_Org_Chem-13-2626-s001.pdf]
